# Coronary Angiography, Intravascular Ultrasound, and Optical Coherence Tomography for Guiding of Percutaneous Coronary Intervention: A Systematic Review and Network Meta-Analysis

**DOI:** 10.1161/CIRCULATIONAHA.123.067583

**Published:** 2024-02-12

**Authors:** Daniele Giacoppo, Claudio Laudani, Giovanni Occhipinti, Marco Spagnolo, Antonio Greco, Carla Rochira, Federica Agnello, Davide Landolina, Maria Sara Mauro, Simone Finocchiaro, Placido Maria Mazzone, Nicola Ammirabile, Antonino Imbesi, Carmelo Raffo, Sergio Buccheri, Davide Capodanno

**Affiliations:** Division of Cardiology, Azienda Ospedaliero-Universitaria Policlinico “Rodolico – San Marco,” University of Catania, Italy.

**Keywords:** coronary angiography, coronary artery disease, drug-eluting stents, intravascular imaging, intravascular ultrasound, optical coherence tomography, percutaneous coronary intervention

## Abstract

**BACKGROUND:**

Results from multiple randomized clinical trials comparing outcomes after intravascular ultrasound (IVUS)– and optical coherence tomography (OCT)–guided percutaneous coronary intervention (PCI) with invasive coronary angiography (ICA)–guided PCI as well as a pivotal trial comparing the 2 intravascular imaging (IVI) techniques have provided mixed results.

**METHODS:**

Major electronic databases were searched to identify eligible trials evaluating at least 2 PCI guidance strategies among ICA, IVUS, and OCT. The 2 coprimary outcomes were target lesion revascularization and myocardial infarction. The secondary outcomes included ischemia-driven target lesion revascularization, target vessel myocardial infarction, death, cardiac death, target vessel revascularization, stent thrombosis, and major adverse cardiac events. Frequentist random-effects network meta-analyses were conducted. The results were replicated by Bayesian random-effects models. Pairwise meta-analyses of the direct components, multiple sensitivity analyses, and pairwise meta-analyses IVI versus ICA were supplemented.

**RESULTS:**

The results from 24 randomized trials (15 489 patients: IVUS versus ICA, 46.4%, 7189 patients; OCT versus ICA, 32.1%, 4976 patients; OCT versus IVUS, 21.4%, 3324 patients) were included in the network meta-analyses. IVUS was associated with reduced target lesion revascularization compared with ICA (odds ratio [OR], 0.69 [95% CI, 0.54–0.87]), whereas no significant differences were observed between OCT and ICA (OR, 0.83 [95% CI, 0.63–1.09]) and OCT and IVUS (OR, 1.21 [95% CI, 0.88–1.66]). Myocardial infarction did not significantly differ between guidance strategies (IVUS versus ICA: OR, 0.91 [95% CI, 0.70–1.19]; OCT versus ICA: OR, 0.87 [95% CI, 0.68–1.11]; OCT versus IVUS: OR, 0.96 [95% CI, 0.69–1.33]). These results were consistent with the secondary outcomes of ischemia-driven target lesion revascularization, target vessel myocardial infarction, and target vessel revascularization, and sensitivity analyses generally did not reveal inconsistency. OCT was associated with a significant reduction of stent thrombosis compared with ICA (OR, 0.49 [95% CI, 0.26–0.92]) but only in the frequentist analysis. Similarly, the results in terms of survival between IVUS or OCT and ICA were uncertain across analyses. A total of 25 randomized trials (17 128 patients) were included in the pairwise meta-analyses IVI versus ICA where IVI guidance was associated with reduced target lesion revascularization, cardiac death, and stent thrombosis.

**CONCLUSIONS:**

IVI-guided PCI was associated with a reduction in ischemia-driven target lesion revascularization compared with ICA-guided PCI, with the difference most evident for IVUS. In contrast, no significant differences in myocardial infarction were observed between guidance strategies.

**Clinical Perspective**

**What Is New?**

- This study showed that intravascular ultrasound was associated with lower target lesion revascularization, whereas there was no significant difference between optical coherence tomography and invasive coronary angiography. Analyses revealed significant network inconsistency, mainly attributable to the ILUMIEN IV trial (Optical Coherence Tomography [OCT] Guided Coronary Stent Implantation Compared With Angiography: A Multicenter Randomized Trial in PCI).
- Myocardial infarction was not significantly different between guidance strategies.
- Optical coherence tomography–guided percutaneous coronary intervention was associated with reduced cardiac death and stent thrombosis compared with invasive coronary angiography–guided percutaneous coronary intervention. Nevertheless, these results were driven by individual trials and showed inconsistency across analyses.
- In the pairwise comparisons of intravascular imaging (IVI)– versus invasive coronary angiography–guided percutaneous coronary intervention, IVI guidance was associated with lower target lesion revascularization, ischemia-driven target lesion revascularization, target vessel revascularization, cardiac death, and stent thrombosis. Target vessel myocardial infarction was reduced only in the frequentist analysis.

**What Are the Clinical Implications?**

- The use of IVI for guiding percutaneous coronary intervention improves long-term clinical outcomes, especially target lesion revascularization. However, the benefits of IVI seem to be predominantly driven by intravascular ultrasound guidance, whereas more uncertainty surrounds optical coherence tomography guidance.
- The strength of evidence on prognostically relevant end points, such as cardiac death, myocardial infarction, and stent thrombosis, warrants more data and analyses because inconsistent results were observed across trials, clinical settings, and statistical methods.
- The benefits of IVI may be driven by specific, complex coronary artery disease patterns that require proper delineation by appropriate individual patient data analyses.

**Editorial, see p 1087**

Invasive coronary angiography (ICA) is the ordinary guidance for percutaneous coronary intervention (PCI). Nevertheless, ICA provides only a global, 2-dimensional view of coronary artery structures that comes with inherent limitations to comprehensively assess atherosclerotic burden, discern plaque characteristics, define vessel diameter, ensure optimal stent expansion, and identify acute complications including stent edge dissections, stent mal-apposition, tissue protrusion, and endoluminal thrombosis.^1,2^ Against this background, intravascular imaging (IVI) techniques such as intravascular ultrasound (IVUS) and optical coherence tomography (OCT) have emerged as complementary diagnostic tools to overcome ICA shortcomings by serial cross-sectional images of the arteries.^1,2^

IVUS relies on mechanical or multielement phased array transducers mounted at the catheter tip, emitting and receiving the ultrasound reflection off the arterial structures.^1,2^ Although more than 3 decades have elapsed since the introduction of IVUS in clinical practice, large randomized trials comparing IVUS- with ICA-guided PCI have been conducted only in recent years.^3,4^ The results of these trials generally showed a lower incidence of target vessel failure in patients assigned to IVUS-guided PCI compared with ICA-guided PCI primarily attributable to a significant reduction in target lesion revascularization.^3,4^ OCT is a technique based on near-infrared light emission that gained popularity because of a spatial resolution (10–20 μm) about 10× higher than that of IVUS (≈150 μm).^1,2^ OCT capabilities make this technology potentially superior to IVUS in determining the appropriate stent size, assessing acute PCI outcomes, and guiding optimal stent expansion,^5,6^ yet there has been a lack of trial results supporting this hypothesis in terms of clinical outcomes.^5,6^

In light of the uncertainty surrounding the role of IVUS and OCT compared with ICA for guiding PCI and the substantial amount of additional evidence from recent randomized trials, it was decided to conduct a comprehensive and updated frequentist and Bayesian network meta-analysis comparing ICA-, IVUS-, and OCT-guided PCI. The network meta-analyses were complemented with secondary pairwise meta-analyses comparing IVI- with ICA-guided PCI to provide a more general research question and enhance the statistical power for the assessment of key individual outcomes.

## METHODS

This study follows the recommendations of Preferred Reporting for network (PRISMA-NMA) and pairwise meta-analyses (PRISMA) of randomized clinical trials (Tables S1 and S2) and Cochrane Collaboration.^7–9^ The protocol was registered with PROSPERO (CRD42023455920) (Supplemental Material, Protocol Registration). No institutional review board approval was required for this type of study.

### Eligibility Criteria

Trials could be included in the network meta-analyses when the following criteria were satisfied: (1) patients from any clinical setting and with any coronary artery disease pattern undergoing PCI; (2) implantation of drug-eluting stents; (3) random allocation to at least 2 PCI guidance strategies among ICA, IVUS, and OCT; and (4) clinical follow-up >6 months. Trials comparing IVI- (OCT and IVUS) versus ICA-guided PCI that met all the other inclusion criteria were included in the secondary pairwise meta-analyses.

### Search, Data Extraction, and Qualitative Assessment

Search strategy, data extraction, and the methods used to assess the risk of bias and the reliability of results are reported in the Supplemental Material (Supplemental Methods; Table S3; Figure S1).^10,11^

### Outcomes

The prespecified primary and coprimary outcomes were target lesion revascularization and myocardial infarction, respectively. Secondary outcomes included ischemia-driven target lesion revascularization, target vessel myocardial infarction, all-cause death, cardiac death, target vessel myocardial infarction, stent thrombosis, and major adverse cardiac events. The preferential follow-up time was 24 months. More details on the outcomes and the criteria used to solve inconsistencies are provided in the Supplemental Material (Supplemental Methods; Tables S4 through S6).

### Statistical Analyses

In the Supplemental Methods, a brief description of the differences between frequentist and Bayesian analyses and network and pairwise meta-analyses is provided.

Outcomes between PCI guidance strategies, as defined in the original intention-to-treat analyses, were combined by hierarchical frequentist and Bayesian random-effects consistency models and reported by odds ratios (ORs) or incidence rate ratio patient-years of follow-up and 95% CIs or credible intervals, as appropriate.^9^ The network of evidence was visually and numerically assessed in terms of weights, comparisons, and individual trial influence for each outcome.^12,13^ In Bayesian analyses, overdispersed vague priors for a common distribution mean effect and uniform between-trial heterogeneity random-effects parameters were given.^14^ Models were computed by Markov chain Monte Carlo simulations, using 4 chains with overdispersed initial values, and Gibbs sampling was based on 100 000 iterations after a burn-in phase of 50 000 iterations. Convergence was evaluated according to Brooks-Gelman-Rubin.^14^ PCI guidance strategies were ranked according to their probability to have a certain rank and the surface under the cumulative ranking curve.^15^ The consistency between direct and indirect evidence was assessed locally by node split (ie, split of the contributions to each comparisons into direct and indirect evidence and assessment of the contrast between the 2 components of the evidence) as well as visual and numerical assessment of the effects attributable to each component of the evidence within and between comparisons, and globally by inconsistency models.^12,16,17^ Direct comparisons between PCI guidance strategies within the networks were also assessed by pairwise meta-analyses.^12,16,17^ Results were displayed by using forest plots illustrating the relative contribution of individual trials.^9^ Within-comparison between-trial heterogeneity was assessed according to *τ*^2^ and I^2^ statistics, prediction intervals, visual exploration of individual trial heterogeneity against individual trial impact, prediction intervals for the expected treatment effect of a new trial testing the PCI guidance strategies, and influence analyses targeting potential outlier effects.^9,18^ Prespecified sensitivity analyses were conducted by outcomes definition, using an estimator accounting for between-trial heterogeneity in follow-up length (ie, incidence rate ratios), comparing outcomes at the longest available follow-up, excluding trials that were not intended to assess clinical outcomes at mid- to long-term follow-up, excluding trials with a high risk of bias in one or more components of the Risk of Bias (RoB) 2 tool, excluding small trials, excluding trials using IVI only for stent optimization, and exploring difference between regions. Other prespecified sensitivity analyses included meta-analyses in specific subgroups and meta-regressions to explore the influence of diabetes, acute coronary syndrome, complex coronary artery disease, chronic total occlusions, and bifurcation lesions.

Pairwise meta-analyses were also conducted to assess the effect of IVI, regardless of the technique used, compared with ICA. Frequentist fixed- and random-effects model estimates were complemented by frequentist random-effects model estimates with 95% CI according to the Hartung–Knapp method and Bayesian random-effects model estimates.^9^

Finally, the impact of small-study effects and publication bias was inspected by contour-enhanced comparison-adjusted (ie, network meta-analyses) and standard funnel plots (ie, pairwise IVI versus ICA analyses) and formally assessed by Egger’s test.^9,12^ Statistical analysis was conducted by using R 4.3.1 and STATA 18.

## RESULTS

**Figure 1.**
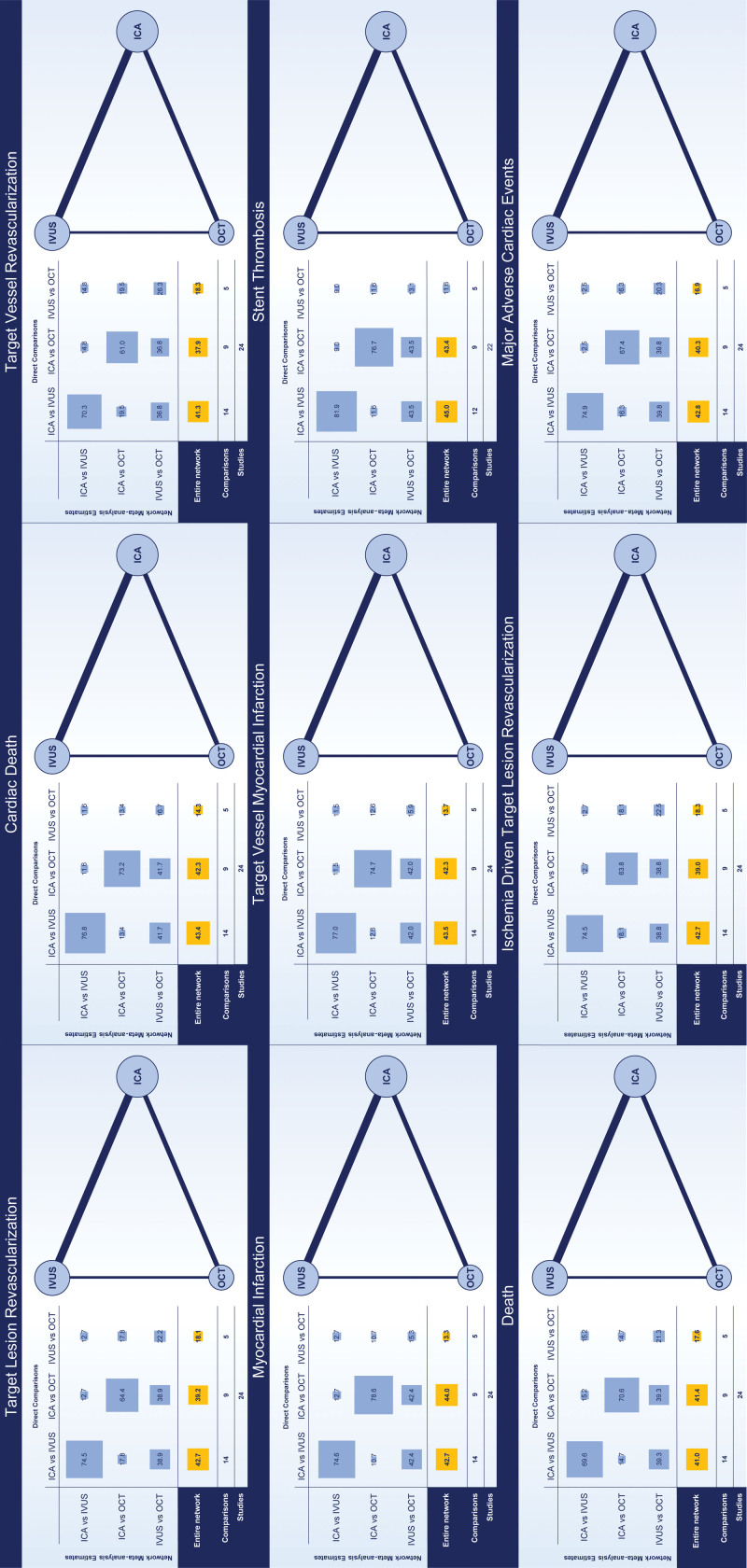
**Networks of the evidence.** The network of the evidence for each outcome is illustrated. The size of nodes (ie, guidance strategies) is proportional to the number of patients pooled. The thickness of the connection between nodes is proportional to the number of comparisons. The distribution of the evidence across the comparison and the entire network is illustrated in the Table. ICA indicates invasive coronary angiography; IVUS, intravascular ultrasound; and OCT, optical coherence tomography.

The search and study selection processes are illustrated in Table S3 and Figure S1. A total of 24 randomized trials (15 489 patients: IVUS versus ICA, 46.4%, 7189 patients; OCT versus ICA, 32.1%, 4976 patients; OCT versus IVUS, 21.4%, 3324 patients) were included in the network meta-analyses (Table 1).^3,4,6,19–47^ The RENOVATE-COMPLEX-PCI trial (Randomized Controlled Trial of Intravascular Imaging Guidance Versus Angiography-Guidance on Clinical Outcomes After Complex Percutaneous Coronary Intervention; 1639 patients) was deemed eligible only for the secondary pairwise meta-analyses because patients randomized to IVI could receive IVUS or OCT at the physician’s discretion.^48^ No trial including ICA guidance systematically used quantitative coronary angiography and stent enhancement techniques for PCI optimization. The network of evidence is illustrated for each outcome in Figure 1. The design of trials was predominantly 2-arm, except for the ILUMIEN III (OPTIMIZE PCI: Multicenter Randomized Trial of OCT Compared to IVUS and Angiography to Guide Coronary Stent Implantation) and iSIGHT (Optical Coherence Tomography Versus Intravascular Ultrasound and Angiography to Guide Percutaneous Coronary Interventions) trials, which were 3-arm (ie, ICA versus IVUS versus OCT).^3,4,6,19–48^ The individual sample size ranged from 80 to 2487 patients, 17 trials were multicenter, 16 trials were conducted exclusively in East Asia, and 16 trials intended to primarily assess mid- to long-term clinical outcomes.^3,4,6,19–48^ Overall, median follow-up ranged from approximately 6 to 30 months for a total of ≈20 500 patient-years.^3,4,6,19–48^ Main clinical and angiographic characteristics, inclusion and exclusion criteria, and reported outcomes across trials are summarized in Tables S7 through S9. Mean age ranged from 55.0 to 76.1 years (weighted mean, 64.4 years), female sex ranged from 15.1% to 46.9% (mean prevalence, 25.9%), and diabetes prevalence ranged from 13.0% to 100% (mean prevalence, 33.5%).^3,4,6,19–48^ Despite variable prevalences of multivessel disease across trials, target lesions per patient were not >1.6 (weighted mean, 1.2).^3,4,6,19–48^ Bifurcation disease was an exclusion criterion in some trials and a mandatory inclusion criterion in the OCTOBER trial (European Trial on Optical Coherence Tomography Optimized Bifurcation Event Reduction).^3,4,6,19–48^ Similarly, left main disease was an exclusion criterion in some trials and a mandatory inclusion criterion in the trial by Liu et al.^3,4,6,19–48^ Two trials comparing IVUS versus ICA focused only on chronic total occlusion.^3,4,6,19–48^ The qualitative assessment of trials is reported in Figures S2 and S3.

**Table 1.**
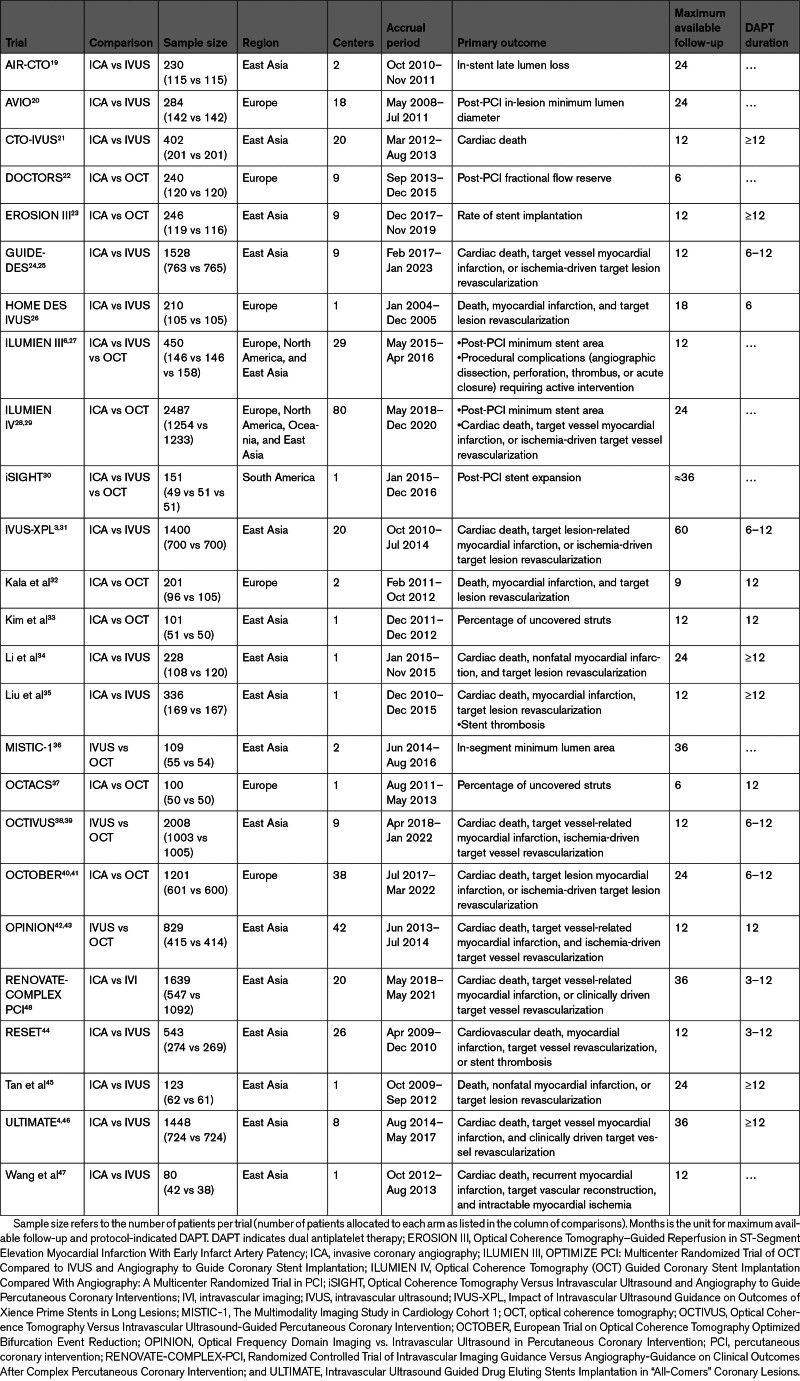
Main Characteristics of the Trials

### Primary and Coprimary Outcomes

**Figure 2.**
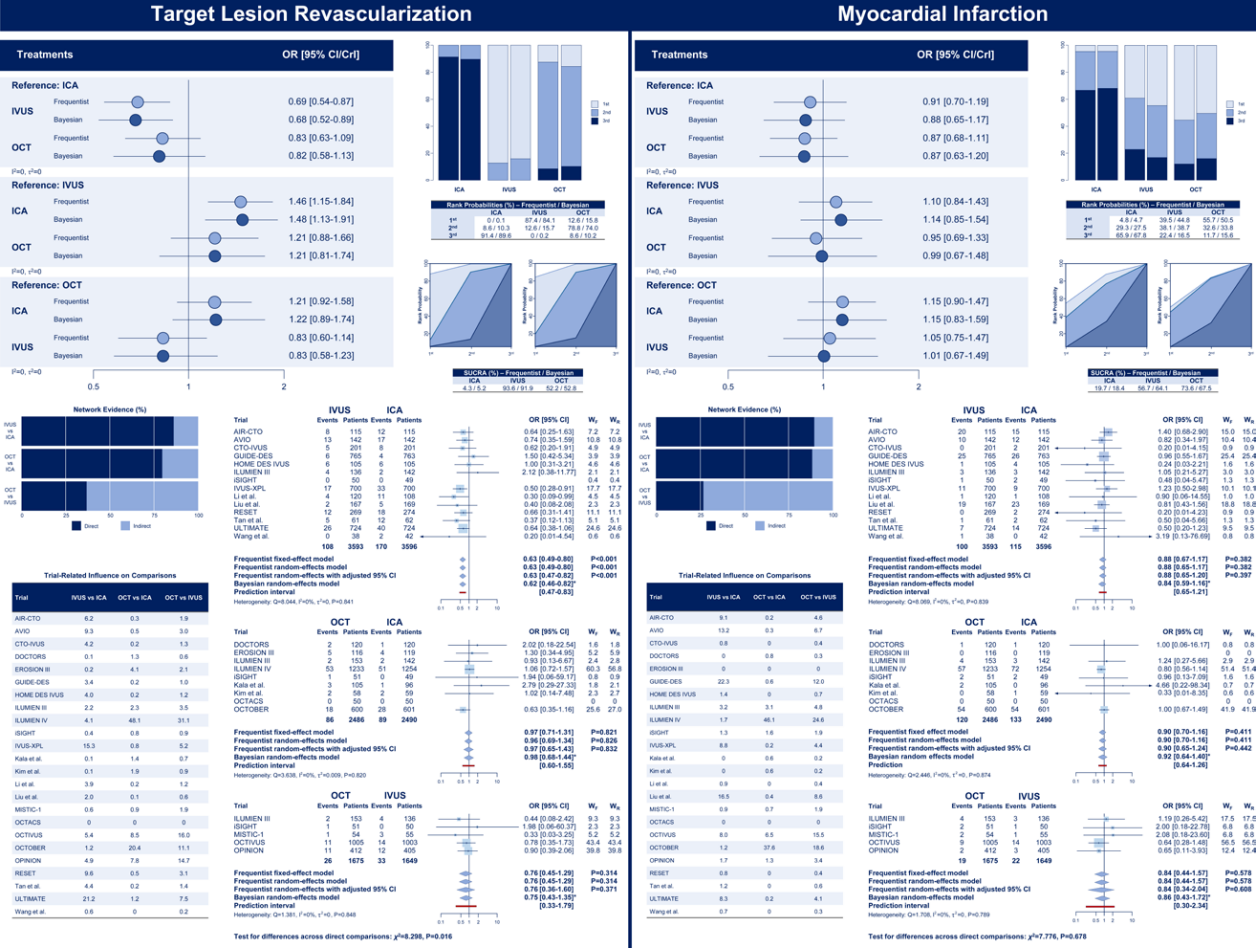
**Target lesion revascularization and myocardial infarction: network comparisons. Left**, Target lesion revascularization. **Right**, Myocardial infarction. The results of the frequentist (light blue) and network meta-analyses (dark blue) are illustrated in the forest plot located in the **top** portion of each of the 2 panels along with the ranking probabilities (ie, bar plots with the corresponding table) and the SUCRA (ie, surface plots with the corresponding table). The distributions of the direct and indirect components of the evidence between comparisons and trials are respectively illustrated in the bar plots and the table at **bottom left** portion of each panel. Pairwise meta-analyses of the direct component of evidence by comparison are illustrated at **bottom right** portion of each panel. Myocardial infarction estimates in the IVUS-XPL trial (Impact of Intravascular Ultrasound Guidance on Outcomes of Xience Prime Stents in Long Lesions) include periprocedural events. *Credible interval. CrI indicates credible interval; ICA, invasive coronary angiography; IVUS, intravascular ultrasound; OCT, optical coherence tomography; OR, odds ratio; SUCRA, surface under the cumulative ranking curve; W_F_, weights by fixed-effect model; and W_R_, weights by random-effects model.

With respect to the primary outcome of target lesion revascularization, in the frequentist analysis, IVUS was associated with reduced target lesion revascularization compared with ICA (OR, 0.69 [95% CI, 0.54–0.87]), whereas no significant differences were observed between OCT and ICA (OR, 0.83 [95% CI, 0.63–1.09]) and OCT versus IVUS (OR, 1.21 [95% CI, 0.88–1.66]; Table 2; Figure 2). IVUS showed the highest probability of being ranked as the best strategy (rank first: 87.4%; surface under the cumulative ranking curve, 93.6%) (Table 3; Figure 2). The ILUMIEN IV trial (Optical Coherence Tomography [OCT] Guided Coronary Stent Implantation Compared With Angiography: A Multicenter Randomized Trial in PCI) had a substantial direct influence (48.1%) on the comparison OCT versus ICA and a significant indirect influence on the comparison OCT versus IVUS (31.1%; Figure 2). In direct comparisons as assessed by random-effects models with CI correction, IVUS was associated with reduced target lesion revascularization compared with ICA (OR, 0.63 [adjusted 95% CI, 0.47–0.82]) and no significant differences in the comparisons OCT versus ICA (OR, 0.97 [adjusted 95% CI, 0.65–1.43]) and OCT versus IVUS (OR, 0.76 [adjusted 95% CI, 0.36–1.60]). The relative weights of trials within the IVUS versus ICA comparison were reasonably balanced. In contrast, direct evidence in the comparison OCT versus ICA mainly relied on the ILUMIEN IV trial (56.8%) followed by the OCTOBER trial (27.0%), whereas direct evidence in the comparison OCT versus IVUS was primarily driven by the OCTIVUS (Optical Coherence Tomography Versus Intravascular Ultrasound-Guided Percutaneous Coronary Intervention; 43.4%) and OPINION (Optical Frequency Domain Imaging vs. Intravascular Ultrasound in Percutaneous Coronary Intervention) trials (39.8%; Figure 2). The cumulative effects across the direct comparisons did not reveal significant inconsistency over time (Figure S4). The results by Bayesian random-effects models yielded consistent results (Tables 2 and 3; Figure 2).

**Table 2.**
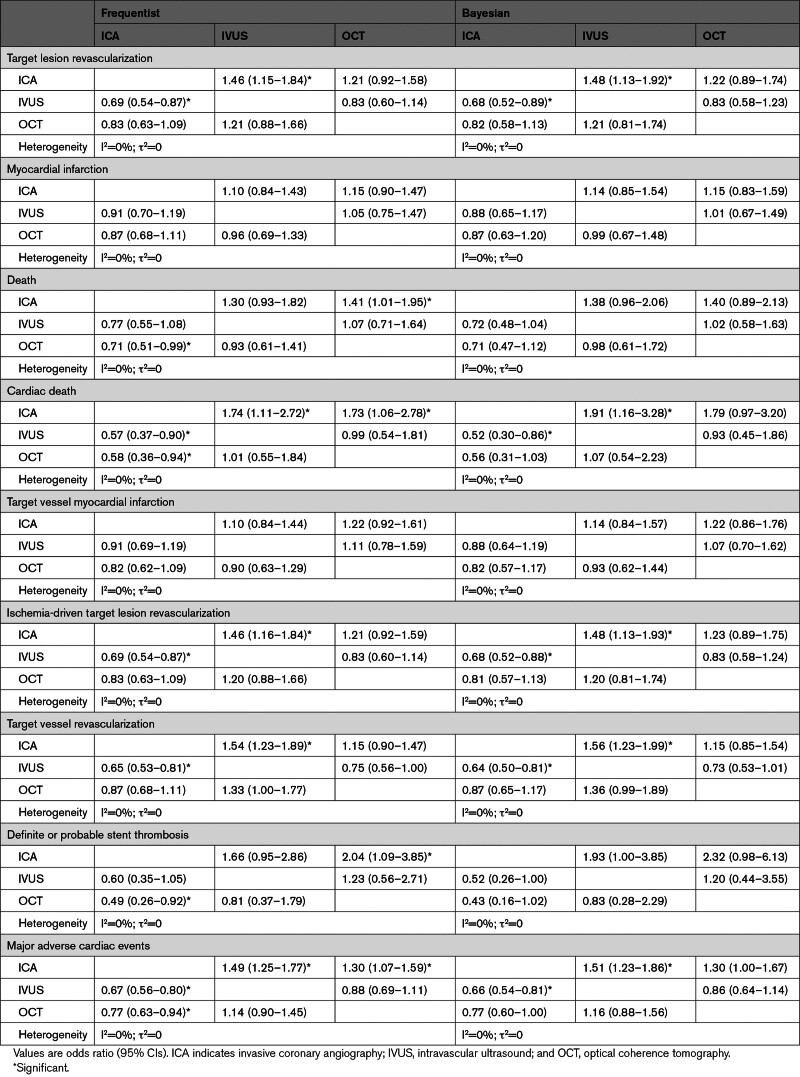
Frequentist Random-Effects Network Meta-Analyses

**Table 3.**
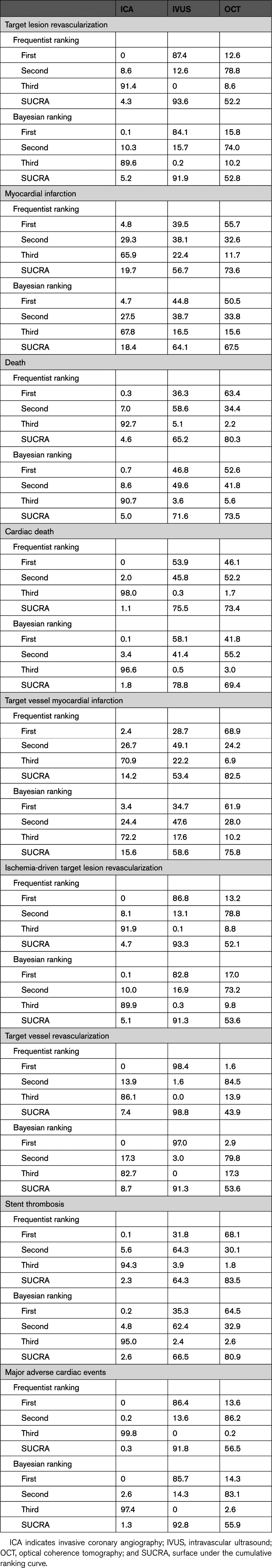
Frequentist and Bayesian Rank Probabilities and SUCRA Values

**Figure 3.**
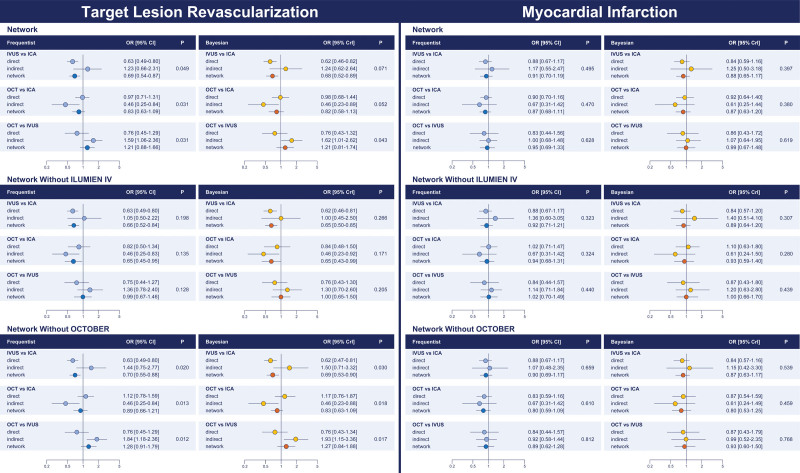
**Target lesion revascularization and myocardial infarction: network node split. Left**, Target lesion revascularization. **Right**, Myocardial infarction. The results of the frequentist (**left**) and Bayesian (**right**) node split in the entire pool of trials (**top**), after removal of ILUMIEN IV from the entire pool of trials (**middle**), and after removal of OCTOBER from the entire pool of trials (**bottom**) are illustrated. The direct and indirect components of the evidence (frequentist: light blue; Bayesian: yellow) are reported along with the combined evidence (ie, network meta-analysis; frequentist: dark blue; Bayesian: orange) and the *P* for inconsistency. CrI indicates credible interval; ICA, invasive coronary angiography; ILUMIEN IV, Optical Coherence Tomography (OCT) Guided Coronary Stent Implantation Compared With Angiography: A Multicenter Randomized Trial in PCI; IVUS, intravascular ultrasound; OCT, optical coherence tomography; OCTOBER, European Trial on Optical Coherence Tomography Optimized Bifurcation Event Reduction; and OR, odds ratio.

Frequentist node split revealed significant inconsistency, especially in the comparisons involving OCT (IVUS versus ICA, *P*_inconsistency_=0.049; OCT versus ICA, *P*_inconsistency_=0.031; OCT versus IVUS, *P*_inconsistency_=0.031) (Table 4; Figure 3). The Bayesian analysis showed a slight mitigation of the effects (Table 4; Figure 3). The assessment of global network coherence by inconsistency model revealed a higher deviance information criterion (Table S10). After excluding ILUMIEN IV from the entire set of trials, significant inconsistency was no longer detectable (OCT versus ICA, *P*_inconsistency_=0.198; IVUS versus ICA, *P*_inconsistency_=0.135; OCT versus IVUS, *P*_inconsistency_=0.128; Figure 3; Tables S11 and S12). After excluding the OCTOBER trial from the entire set of trials, significant inconsistency became more evident (OCT versus ICA, *P*_inconsistency_=0.020; IVUS versus ICA, *P*_inconsistency_=0.013; OCT versus IVUS, *P*_inconsistency_=0.012; Figure 3; Tables S13 and S14). The results were consistent in the Bayesian analysis (Figure 3; Tables S15 through S18).

**Table 4.**
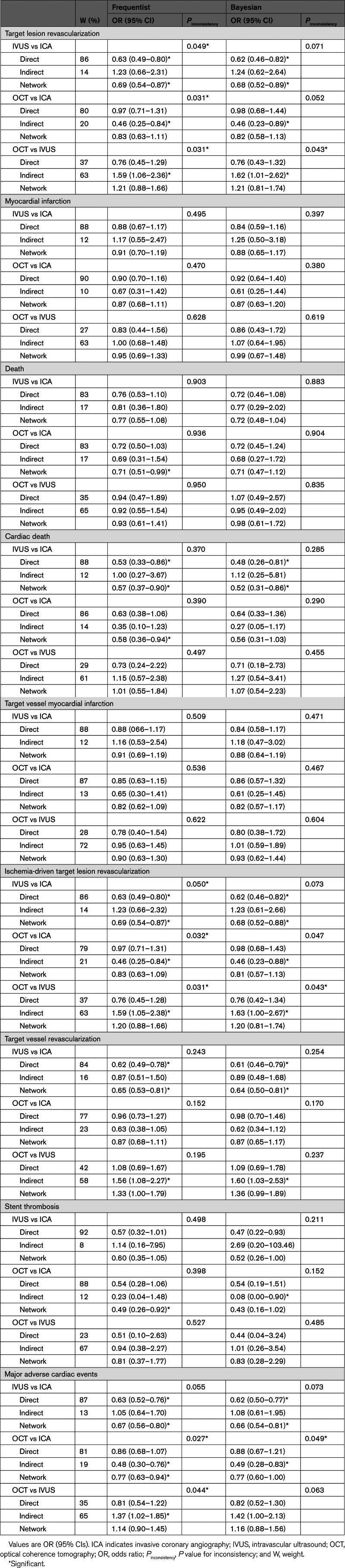
Frequentist and Bayesian Network Node Split

**Figure 4.**
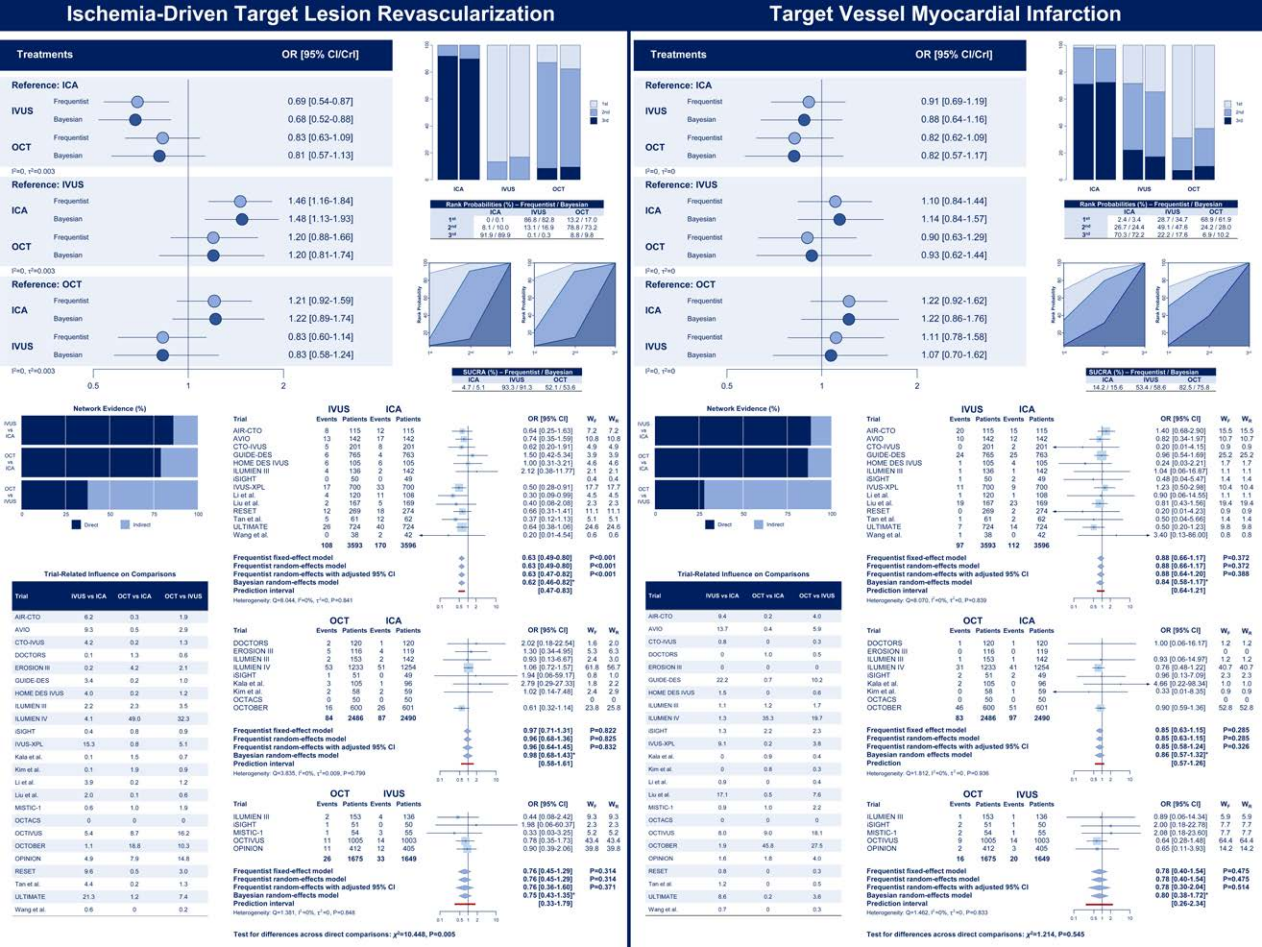
**Ischemia-driven target lesion revascularization and target vessel myocardial infarction: network comparisons. Left**, Ischemia-driven target lesion revascularization. **Right**, Target vessel myocardial infarction. The results of the frequentist (light blue) and network meta-analyses (dark blue) are illustrated in the forest plot located in the **top** portion of each of the 2 panels along with the ranking probabilities (ie, bar plots with the corresponding table) and the SUCRA (ie, surface plots with the corresponding table). The distribution of the direct and indirect components of the evidence between comparisons and trials are respectively illustrated in the bar plots and the table in the **bottom left** portion of each panel. Pairwise meta-analyses of the direct component of evidence by comparison are illustrated in the **bottom right** portion of each panel. Myocardial infarction estimates in the IVUS-XPL trial (Impact of Intravascular Ultrasound Guidance on Outcomes of Xience Prime Stents in Long Lesions) include periprocedural events. *Credible interval. CrI indicates credible interval; ICA, invasive coronary angiography; IVUS, intravascular ultrasound; OCT, optical coherence tomography; OR, odds ratio; SUCRA, surface under the cumulative ranking curve; W_F_, weights by fixed-effect model; and W_R_, weights by random-effects model.

With respect to the coprimary outcome of myocardial infarction, there were no differences between guidance strategies (Tables 2 and 3; Figure 2). Rank probabilities tended to favor OCT in both frequentist and Bayesian analyses (Table 3; Figure 2). The relative weights of trials across direct comparisons encompassed the distributions for target lesion revascularization (Figure 2). The cumulative effects across the direct comparisons did not reveal significant inconsistency over time (Figure S4). The assessment of network coherence did not reveal significant inconsistency, and the removal of the ILUMIEN IV and OCTOBER trials each at a turn was associated with consistent results, regardless of the statistical method used (Figure 4; Tables S10 through S18).

### Secondary Outcomes

The secondary outcomes are illustrated in Tables 2 through 4, Figures 4 through 6, and Figures S5 and S6. Consistently with target lesion revascularization, IVUS-guided PCI was associated with reduced ischemia-driven target lesion revascularization and target vessel revascularization compared with ICA, and significant inconsistency was detected by node split and inconsistency model for ischemia-driven target lesion revascularization (Tables 2 through 4; Figures 4 through 6; Table S10). Rank probabilities and surface under the cumulative ranking curve values associated with IVUS and OCT were higher than those associated with ICA in terms of cardiac death (Table 3). The cumulative meta-analyses of the direct components of the evidence showed overall consistent trends over time (Figure S7).

**Figure 5.**
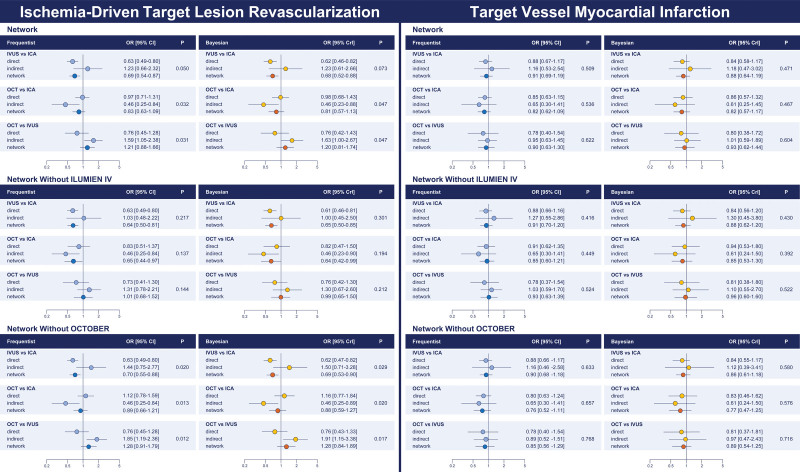
**Ischemia-driven target lesion revascularization and target vessel myocardial infarction: network node split. Left**, Ischemia-driven target lesion revascularization. **Right**, Target vessel myocardial infarction. The results of the frequentist (**left**) and Bayesian (**right**) node split in the entire pool of trials (**top**), after removal of ILUMIEN IV from the entire pool of trials (**middle**), and after removal of OCTOBER from the entire pool of trials (**bottom**) are illustrated. The direct and indirect components of the evidence (frequentist: light blue; Bayesian: yellow) are reported along with the combined evidence (ie, network meta-analysis; frequentist: dark blue; Bayesian: orange) and the *P* for inconsistency. CrI indicates credible interval; ICA, invasive coronary angiography; ILUMIEN IV, Optical Coherence Tomography (OCT) Guided Coronary Stent Implantation Compared With Angiography: A Multicenter Randomized Trial in PCI; IVUS, intravascular ultrasound; OCT, optical coherence tomography; OCTOBER, European Trial on Optical Coherence Tomography Optimized Bifurcation Event Reduction; and OR, odds ratio.

**Figure 6.**
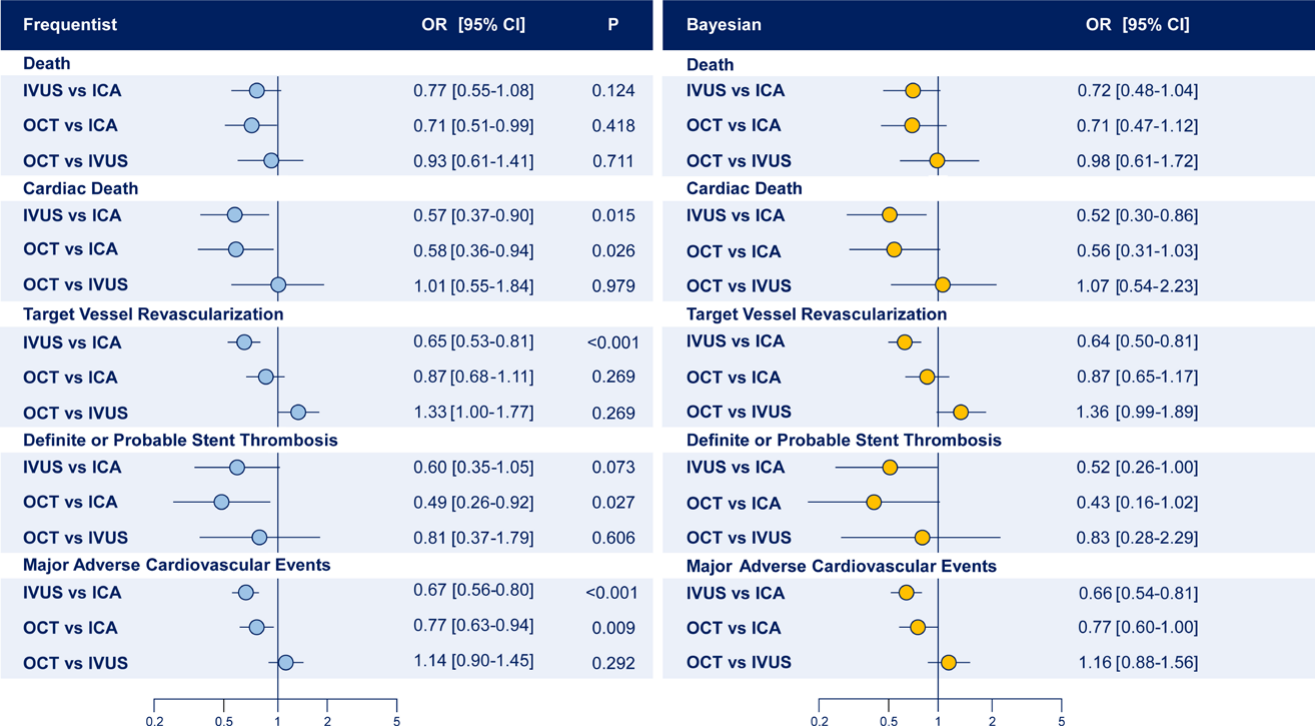
**Secondary outcomes: network meta-analyses.** Secondary outcomes across comparisons as assessed by frequentist (**left**, light blue) and Bayesian (**right**, yellow) network meta-analyses. CrI indicates credible interval; ICA, invasive coronary angiography; IVUS, intravascular ultrasound; OCT, optical coherence tomography; and OR, odds ratio.

In the frequentist analyses, IVUS-guided PCI was associated with a significant reduction in all-cause death compared with ICA-guided PCI, whereas both IVUS- and OCT-guided PCI were associated with a significant reduction in cardiac death compared with ICA-guided PCI (OR, 0.57 [95% CI, 0.37–0.90] and OR, 0.58 [95% CI, 0.36–0.94]). OCT-related effects were significantly mitigated in the Bayesian analyses (Tables 2 through 4; Figure 6). The assessment of direct evidence showed that cardiac death was not significantly different between OCT and ICA in both the frequentist and Bayesian analyses (Figure S5). Rank probabilities and surface under the cumulative ranking curve values associated with IVUS and OCT were higher than those associated with ICA in terms of cardiac death (Table 3). The networks were consistent (Table 4; Table S10).

In the frequentist analysis, OCT-guided PCI was associated with reduced definite or probable stent thrombosis compared with ICA (OR, 0.49 [95% CI, 0.26–0.92]), whereas in the Bayesian analysis, this effect was significantly mitigated (OR, 0.43 [95% CI, 0.16–1.02]; Tables 2 through 4; Figure 6; Figure S6). Moreover, compared with ICA, OCT-related effects on stent thrombosis were no longer detectable after excluding ILUMIEN IV from the entire set of trials, whereas results remained unchanged after excluding the OCTOBER trial (Tables S11 through S18).

Finally, IVUS-guided PCI was associated with lower major adverse cardiac events compared with ICA-guided PCI (Tables 2 and 3; Figure 5). However, node split and inconsistency model revealed inconsistency (Table 4; Table S10).

### Sensitivity Analyses

The replication of analyses by outcomes definition produced overall consistent results except for a mitigation of the IVUS-related reduction observed in ischemia-driven target lesion revascularization (Tables S19 and S20). The use of an estimator accounting for between-trial differences in follow-up length (ie, incidence rate ratio patient-years of follow-up) produced similar results (Tables S21 and S22). The combination of data at the longest available follow-up (Tables S23 and S24), the exclusion of trials presenting a high-risk of bias in one of the domains of RoB 2 (Tables S25 and S26), the exclusion of trials not designed to assess mid- to long-term clinical outcomes (Tables S27 and S28), the exclusion of smaller trials (Tables S29 and S30), and the exclusion of trials using IVI only for stent optimization (Tables S31 and S32) showed overall consistent results with some variations across analyses primarily influencing death and cardiac death (Tables S19 through S32). In addition, a significant reduction in definite or probable stent thrombosis associated with OCT compared with ICA and a possible improvement in target vessel revascularization associated with IVUS over OCT were detected when using the longest available follow-up but only in the frequentist analysis (Tables S23 and S24). The exclusion of the EROSION III trial (Optical Coherence Tomography–Guided Reperfusion in ST-Segment Elevation Myocardial Infarction With Early Infarct Artery Patency) was not associated with different results (Tables S33 through S36).

The inspection of the association between outcomes and key clinical settings (diabetes and acute coronary syndrome) and coronary artery disease patterns (stent length per patient, bifurcation lesions, and chronic total occlusion) by Bayesian meta-regression did not reveal significant associations, and results were generally unchanged (Tables S37 through S41).

Finally, the subgroup of trials exclusively conducted in East Asia (9604 patients) drove the benefits associated with IVI and sensitivity analyses, whereas pooling trials conducted exclusively or predominantly at European and American centers (5304 patients) did not result in significant long-term clinical differences between strategies across the assessed outcomes (Tables S42 through S45).

### IVI- Versus ICA-Guided PCI

**Figure 7.**
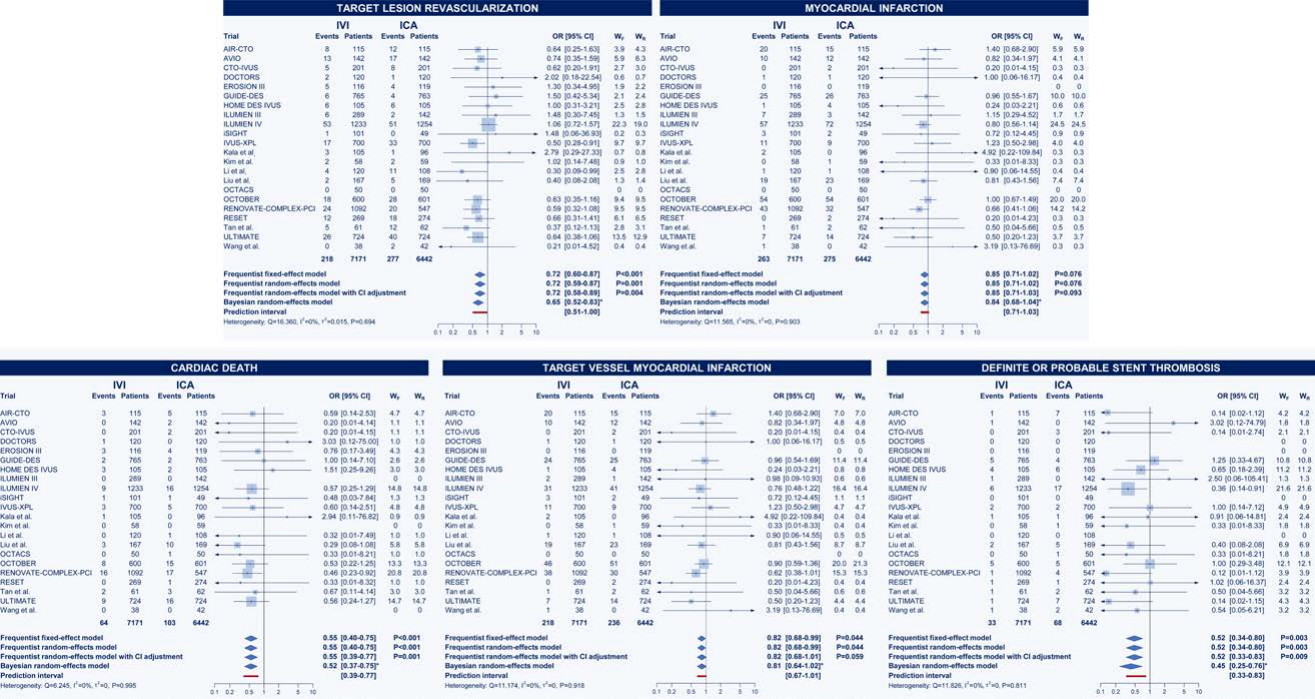
**IVI- vs ICA-guided PCI: pairwise meta-analyses.** Pairwise meta-analyses of trials comparing IVI- vs ICA-guided PCI by frequentist (fixed-effect, random-effects, and random-effects with adjusted CIs by the Hartung-Knapp method) and Bayesian (random-effects) models. The prediction interval is illustrated. *Credible interval. ICA indicates invasive coronary angiography; IVI, intravascular imaging; OR, odds ratio; PCI, percutaneous coronary intervention; W_F_, fixed-effects model weights; and W_R_, random-effects model weights.

**Figure 8.**
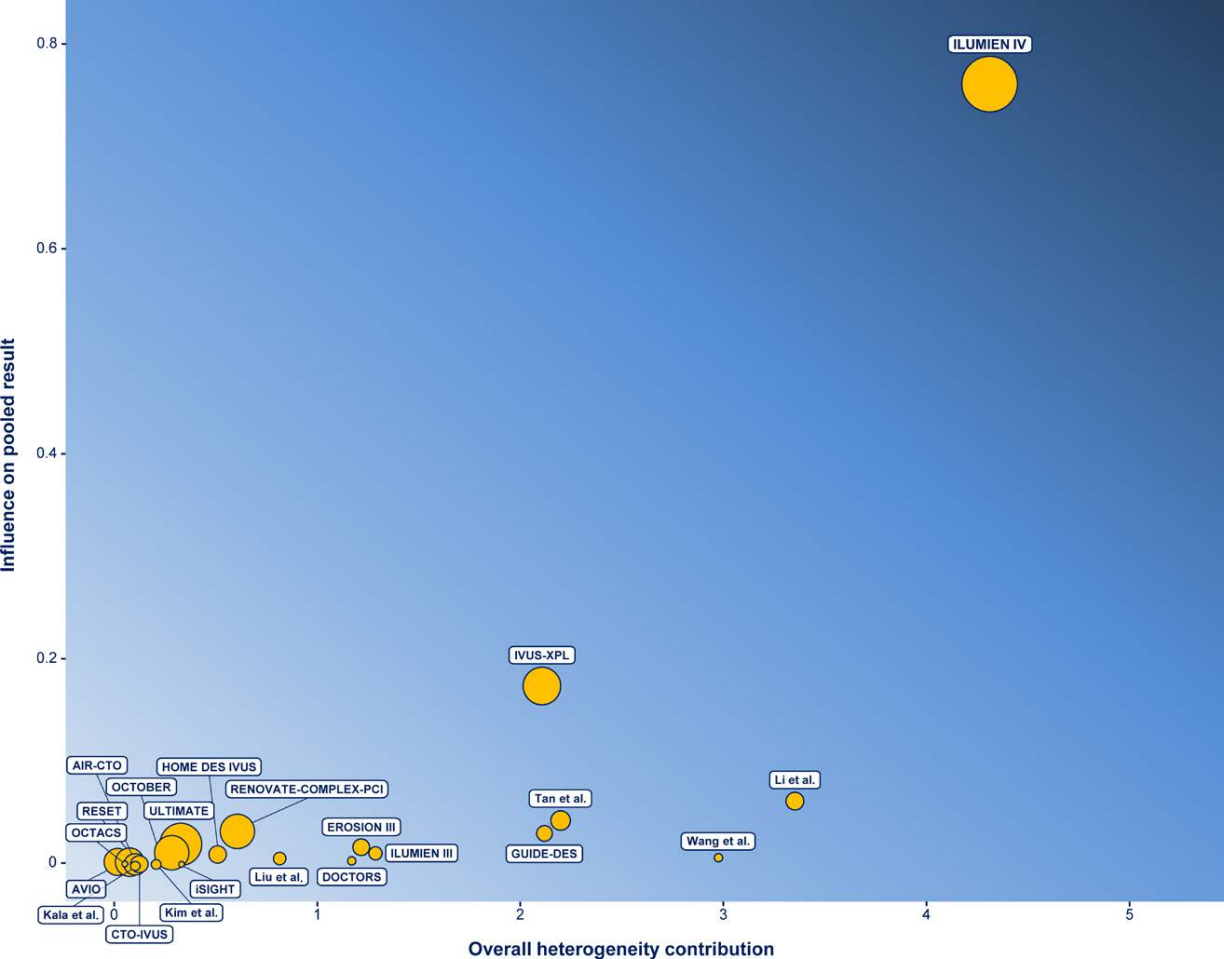
**Influence on summary estimates and heterogeneity of individual trials: target lesion revascularization.** The assessment of the contrast between individual trial influence and heterogeneity for target lesion revascularization in a pairwise meta-analysis intravascular imaging– vs invasive coronary angiography–guided percutaneous coronary intervention revealed that inconsistency was driven by the ILUMIEN IV trial (Optical Coherence Tomography [OCT] Guided Coronary Stent Implantation Compared with Angiography: A Multicenter Randomized Trial in PCI).

Trials comparing only OCT versus IVUS (ie, MISTIC-1 [The Multimodality Imaging Study in Cardiology Cohort 1], OCTIVUS, and OPINION) were excluded. In the frequentist random-effects meta-analyses with 95% CI adjustment, the use of IVI was associated with reduced target lesion revascularization (OR, 0.72 [95% CI, 0.58–0.89]), cardiac death (OR, 0.55 [95% CI, 0.39–0.77]), and stent thrombosis (OR, 0.52 [95% CI, 0.33–0.83]; Table 5; Figure 7). Results were unchanged in the Bayesian analyses. The assessment of the relationship between influence and heterogeneity across trials revealed the inconsistency of ILUMIEN IV in terms of target lesion revascularization (Figure 8). There was a numerical reduction in myocardial infarction that did not result in a significant difference regardless of the statistical method used (Table 5; Figure 7). However, when primarily considering target vessel myocardial infarction, the difference between IVI- and ICA-guided PCI became significant in the frequentist analysis when the 95% CI was not corrected according to the Hartung-Knapp method (OR, 0.82 [95% CI, 0.68–0.99]; Table 5; Figure 7). Sensitivity analyses were generally consistent (Tables S46–S53). The cumulative meta-analyses of target lesion revascularization, myocardial infarction, ischemia-driven target lesion revascularization, and target vessel myocardial infarction between IVI and ICA showed overall consistent trends over time (Figures S8 and S9).

**Table 5.**
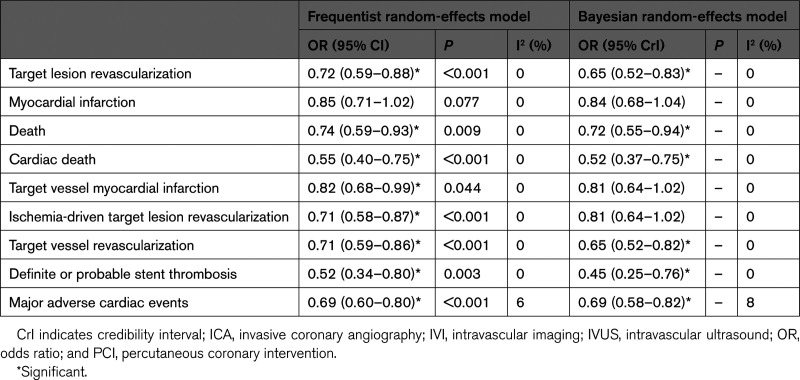
Frequentist and Bayesian Random-Effects Pairwise Meta-Analyses of Trials Comparing IVI- vs ICA-Guided PCI

### Publication Bias and Grading of Evidence

In comparison-adjusted funnel plots, there was evidence of a mild-to-moderate asymmetry for the comparison OCT versus ICA in terms of target lesion revascularization, ischemia-driven target lesion revascularization, and target vessel revascularization, and the comparison OCT versus IVUS in terms of death and target vessel revascularization (Figure S9). However, the Egger’s test was not significant for each outcome (Figure S9). In the assessment of pairwise contour-enhanced funnel plots (ie, IVI versus ICA), for each outcome, there was no apparent evidence of significant publication bias and small-study effects (Figure S9). Most of the individual trial effects across outcomes fell in the area of nonsignificance (Figure S9). The Egger’s test was not significant for each outcome (Figure S10). A Grading of Recommendations, Assessment, Development, and Evaluations (GRADE) summary of evidence is provided in Table S54. In general, the major concerns were associated to the inconsistency affecting some comparisons for the outcomes of target lesion revascularization, ischemia-driven target lesion revascularization, and major adverse cardiac events, the heterogeneity in some outcome definitions, and the dissimilar composition of major adverse cardiac events across trials.

## DISCUSSION

The analysis of available evidence from randomized trials indicates that IVUS-guided PCI was associated with reduced any-type and ischemia-driven target lesion revascularization as well as target vessel revascularization compared with ICA-guided PCI, whereas no significant differences were observed between OCT-guided and ICA-guided PCIs for the same outcomes. However, neither IVUS- nor OCT-guided PCI was associated with reduced myocardial infarction and target vessel myocardial infarction compared with ICA-guided PCI. Although some analyses indicated that IVUS- and OCT-guided PCI were associated with lower mortality and stent thrombosis compared with ICA-guided PCI, these results were significantly influenced by individual trials and the statistical methodology used. When pooling trials comparing IVI- versus ICA-guided PCI, the use of IVI was associated with significant reductions in target lesion revascularization, cardiac death, target vessel myocardial infarction, ischemia-driven target lesion revascularization, target vessel revascularization, and stent thrombosis in the frequentist analyses; the effects in terms of target vessel myocardial infarction and ischemia-driven revascularization were mitigated in the Bayesian analyses.

The results of the RENOVATE-COMPLEX-PCI trial, including 1639 patients randomly assigned to IVI- (IVUS or OCT at the physician’s discretion) or ICA-guided PCI, showed that IVI-guided PCI was associated with decreased target vessel failure because of a significantly lower incidence of cardiac death and numerical reductions in target vessel myocardial infarction and target vessel revascularization.^48^ These findings were considered as a prelude to the upcoming conclusive results of the large-scale, long-awaited ILUMIEN IV and OCTOBER trials, and secondarily as a background for the confirmatory evidence from the OCTIVUS and GUIDE-DES trials.^24,25,28,29,38–41^ However, these trials yielded controversial results. The ILUMIEN IV trial, including a total of 2487 patients with clinical and angiographic high-risk criteria randomly assigned to OCT- or ICA-guided PCI, showed no significant difference in 2-year target lesion failure between guidance strategies.^29^ In contrast, the OCTOBER trial including 1201 patients with bifurcation disease randomly assigned to OCT- versus ICA-guided PCI showed a significant reduction in major adverse cardiac events at 2 years associated with OCT guidance.^41^ The OCTIVUS trial, including 2008 patients randomly assigned to OCT- or IVUS-guided PCI, showed the noninferiority of OCT guidance in terms of 1-year target vessel failure.^39^ Finally, the GUIDE-DES trial (Quantitative Coronary Angiography Versus Intravascular Ultrasound Guidance for Drug-Eluting Stent Implantation) added further uncertainty by showing no significant differences between IVUS- and ICA-guided PCI for all the outcomes.^25^

To the best of our knowledge, there is currently no comprehensive and up-to-date network meta-analysis available on this topic. Previous meta-analyses predated the reporting of numerous large-scale trials, focused upon a single IVI modality, pooled a substantial number of observational studies and historical trials using outdated devices, and used simpler meta-analytic methodology, relying generally only on frequentist statistics, pairwise comparisons, and a very limited number of sensitivity analyses.^49–51^ The present study intends to critically analyze available evidence on ICA, IVUS-guided PCI, and OCT-guided PCI beyond subjective considerations. In a network meta-analysis, indirect comparisons of treatment effects are built on the assumption that studies making different comparisons are similar and exchangeable (ie, transitivity).^7–13^ Consistency or coherence, in this context, refers to the statistical measure of transitivity.^7–13^ In a network meta-analysis, the validation of results relies on the global and local assessment of network consistency.^7–13^

In contrast, significant inconsistency was detected in the present network meta-analyses in terms of target lesion revascularization, ischemia-driven target lesion revascularization, and major adverse cardiac events. In these conditions, direct evidence holds greater reliability than network evidence (ie, the combination of direct and indirect evidence) for these outcomes, and sensitivity analyses showed that the conflict between direct and indirect evidence primarily stems from the ILUMIEN IV trial.^29^ In the comparison OCT versus ICA, the potential advantage of OCT over ICA as promoted by the OCTOBER trial was attenuated by the substantial influence of the ILUMIEN IV trial in terms of relative weight and effect heterogeneity.^29,41^ Consequently, the comparison OCT versus ICA yielded neutral and inconsistent results when set against the comparisons OCT versus IVUS and IVUS versus ICA. Specifically, the comparison OCT versus IVUS did not show significant differences, with a mild numerical advantage toward OCT driven by the OCTIVUS trial, whereas the comparison IVUS versus ICA portrayed a distinctly favorable effect of IVUS across various analytic approaches.^39^ The transitivity assumption is violated because if IVUS and OCT are deemed comparable (OCT versus IVUS comparison) and IVUS is superior to ICA (IVUS versus ICA comparison), it follows that OCT should also be superior to ICA. Yet, as detailed above, this effect was not observed because of the ILUMIEN IV trial, which is the largest trial on the topic.^29^ GUIDE-DES was the other trial providing results on target lesion and vessel revascularization that were not in line with the IVUS-XPL and ULTIMATE (Intravascular Ultrasound Guided Drug Eluting Stents Implantation in “All-Comers” Coronary Lesions) trials.^3,4,25^ Nevertheless, although GUIDE-DES was the largest and most recent trial comparing IVUS- versus ICA-guided PCI, its impact in the meta-analyses was generally negligible because of very low event rates.^25^

Interpreting accumulated evidence is challenging, especially when the granularity of information is variable and individual patient data are not available. The observed differences in the direction and magnitude of effects among available trials demonstrate some heterogeneous patterns across outcomes, frequently complicating the construction of clear explanations. Nevertheless, it is plausible that variations among individual trial outcomes are, to some extent, influenced by diverse clinical conditions and coronary artery disease patterns.^52,53^ In general, the prevalence of diabetes and acute coronary syndrome was heterogeneous across trials. Although diabetes is a major ischemic risk factor and is frequently associated with worse outcomes after revascularization, this condition per se is not synonymous with complex coronary artery disease. In the ILUMIEN IV trial, the inclusion of diabetes among the key inclusion criteria may have produced a study population that was dissimilar to that of other trials.^29^ Similarly, some trials did not include particularly long lesions, and high-risk patterns such as left main disease and chronic total occlusions were generally more represented in IVUS-based trials. Of note, although in general the average number of target lesions across available trials was limited, in ILUMIEN IV, patients had predominatly single-target lesion coronary artery disease.^29^ The OCTOBER trial exclusively included patients with bifurcation disease who in 64.1% of cases required a 2-stent strategy.^41^ In contrast, in the ILUMIEN IV trial, only 3.3% of patients underwent a 2-stent strategy for the treatment of bifurcation disease.^29^ Although this substantial difference may partially explain the different conclusions of the 2 trials, it is worth also noting that in the OCTOBER trial, an explorative subgroup analysis revealed that the main effect was numerically driven by patients who underwent 1-stent strategy PCI.^41^ Against this background, it should also be acknowledged that the recent trials showed lower-than-expected incidences of the primary outcome (ILUMIEN IV control: 8.2% observed versus 12.0% expected; OCTIVUS control: 3.1% observed versus 8.0% expected; GUIDE-DES control: 3.8% observed versus 8.0% expected; OCTOBER control: 14.1% instead of 16.0%).^25,29,39,41^ The coronavirus disease 2019 (COVID-19) pandemic may have had an influence on the observation of incidences lower than anticipated, and it was also involved in the discussion on the heterogeneous results observed across trials, especially the dissimilar trend in target lesion revascularization between ILUMIEN IV and OCTOBER.^29,41^ However, these Western country–based trials were conducted during a similar period, and it is plausible to expect a similar detrimental effect on cardiovascular outcomes reporting and occurrence.^29,41^ Regardless of the possible explanation for the low incidences of events, these findings not only translate into a reduced statistical power to detect differences between the 2 groups but also highlight that the angiographic features used to define coronary artery disease complexity were likely overestimated. Despite these considerations, the multiple sensitivity analyses conducted in the present study did not reveal evident associations with specific high-risk clinical features and coronary artery disease patterns, although this may be a result of the limited flexibility of aggregate-level data and the inherent low statistical power of meta-regression and subgroup analyses. The uncertain findings raise questions about whether the results of certain trials were affected by the inclusion of noncomplex lesions or, conversely, were influenced by the selection of a specific pattern of coronary artery disease that significantly benefited from IVUS or OCT guidance. Nevertheless, differences between exclusively East Asian and primarily non–East Asian trials may provide additional explanations. Indeed, beyond the possible advantages of IVI in treating smaller mean reference vessel diameters in East Asian patients compared with those who are non–East Asian, East Asian operators traditionally have larger experience with IVUS and OCT, and this condition has been linked to improved outcomes.^54^ On the one hand, it is reasonable to hypothesize that operator expertise in East Asian trials may have explained the difference with some non–East Asian trials such as ILUMIEN IV.^29^ On the other hand, it should be acknowledged that acute angiographic, imaging, and clinical outcomes in ILUMIEN IV and other primarily Western country–based trials such as OCTOBER were excellent, potentially indicating a limited impact of the operators’ expertise in the different conclusions of available trials.^29,41^

In contrast with revascularization outcomes, the network was consistent for death and cardiac death. Of note, some sensitivity analyses revealed a possible benefit from IVUS and OCT in terms of death and cardiac death, especially in the frequentist framework. This relevant result was in line with the RENOVATE-COMPLEX-PCI trial (included only in the pairwise meta-analyses IVI versus ICA), in which cardiac death was significantly reduced whereas target vessel myocardial infarction and target vessel revascularization did not reach the statistical significance, and derived from the individual effects of large trials using IVUS (ie, EXCELLENT and IVUS-XPL) and OCT (ie, ILUMIEN IV and OCTOBER) showing consistent direction and magnitude.^3,4,29,41,48^ It is important to note that the combination of data in the pairwise meta-analyses comparing IVI with ICA clearly indicated that IVI is associated with lower cardiac death, but in some sensitivity analyses this result was mitigated and the differences observed across trials may indicate a multifactorial explanation. In this regard, in the network meta-analyses assessing myocardial infarction and target vessel myocardial infarction, significant reductions associated with IVUS- or OCT-guided PCI compared with ICA-guided PCI were not detected. The combination of data in the pairwise meta-analyses IVI versus ICA showed a borderline decrease in target vessel myocardial infarction associated with IVI, only in the frequentist computation. In light of these results, the decrease in stent thrombosis observed after IVI-guided PCI compared with ICA-guided PCI may have contributed to the reduction in cardiac death associated with IVI. Indeed, in clinical trials, sudden death is generally adjudicated as having a cardiac cause, and stent thrombosis is among the established factors leading to sudden death. However, this hypothesis remains largely unconfirmed, and the effects of IVUS and OCT in terms of stent thrombosis were unstable across analyses and between statistical methods. Moreover, the association between reduced stent thrombosis and OCT guidance essentially relied on the ILUMIEN IV trial, whereas in the OCTOBER trial, OCT- and ICA-guided PCI experienced the same number of stent thromboses.^29,41^

In summary, the present study highlights that IVI guidance for PCI improves clinical outcomes, primarily target lesion revascularization, cardiac death, and stent thrombosis. These results are driven by the trials using IVUS. However, accrued evidence is still insufficient, especially for the crucial outcomes of target vessel myocardial infarction and stent thrombosis, and more analysis is warranted to elucidate the reasons for the inconsistent spectrum of outcome improvements between trials favoring IVUS or OCT compared with ICA and understand whether the prognostic advantages of IVI are linked to specific patterns of coronary artery disease. In addition, although OCT provides more valuable and informative images compared with IVUS, there remains uncertainty about whether these advantages translate into improved outcomes after OCT-based stent optimization and acute assessment of PCI results. In comparison with IVUS, the technical advantages of OCT may be more valuable for assessing the pattern of coronary artery disease and less relevant for improving the results of PCI.

### Limitations

First, the absence of access to individual patient data hindered the capability to discern the factors contributing to dissimilar conclusions across trials. Nevertheless, multiple sensitivity analyses were conducted to identify the clinical settings and coronary artery disease patterns that would gain more benefits from IVI guidance during PCI. Second, there was inconsistency in outcomes definition and reporting across trials. Yet this limitation would be challenging to address even with individual patient data because it would require a new independent retrospective adjudication of events. In any case, the sensitivity analyses by using the restricted pool of trials reporting outcomes with more consistent definitions did not change the main conclusions of the study. Moreover, the present study intentionally avoided focusing on major adverse events because of the extreme, unmanageable heterogeneity across trials. Finally, follow-up length differed across trials. However, in the primary analyses, almost all trials exhibited a median follow-up ranging from 12 to 24 months, and final follow-up data from the ULTIMATE (ie, 3 years) and IVUS-XPL (ie, 5 years) trials were deliberately not used to reduce heterogeneity in follow-up length. It is important to note that the sensitivity analysis accounting for differences in follow-up length by incidence rate ratios computed from approximated incident rate patient-years of follow-up between groups did not reveal overall significant inconsistency.^3,4,6,19–43,45–48^ Nevertheless, the sensitivity analysis including data at the longest available follow-up generally aligned with the main results, except for secondary variations observed only in the frequentist computations.

### Conclusions

IVI-guided PCI was associated with reduced any-type and ischemia-driven target lesion revascularization compared with ICA-guided PCI, with the difference most evident for IVUS. In contrast, no significant differences in myocardial infarction and target vessel myocardial infarction were observed.

## ARTICLE INFORMATION

### Sources of Funding

This study was supported by the Department of General Surgery and Surgical-Medical Specialties of the University of Catania, Italy.

### Disclosures

D.C. reports speaker or consulting fees from Amgen, Arena, Daiichi Sankyo, Sanofi, and Terumo; and institutional fees from Medtronic. The other authors report no conflicts.

### Supplemental Material

Supplemental Methods

Tables S1–S54

Figures S1–S11

## Supplementary Material


