# Supplementary material for: Coronary Angiography, Intravascular Ultrasound, and Optical Coherence Tomography for Guiding of Percutaneous Coronary Intervention: A Systematic Review and Network Meta-Analysis
Source: Circulation. 2024 Feb 12;149(14):1065–86. doi: 10.1161/CIRCULATIONAHA.123.067583 (PMC10980178; doi:10.1161/CIRCULATIONAHA.123.067583)
Supplement: Supplementary file 1 [file cir-149-1065-s001.pdf]

SUPPLEMENTARY APPENDIX

Coronary Angiography, Intravascular-Ultrasound, and Optical Coherence Tomography in the Guidance of Percutaneous Coronary Intervention: A Systematic Review and Network Meta-Analysis.

Daniele Giacoppo, MD, MSc, PhD;* Claudio Laudani, MD, MSc;* Giovanni Occhipinti, MD; Marco Spagnolo, MD; Antonio Greco, MD; Carla Rochira, MD; Federica Agnello, MD, Davide Landolina, MD; Maria Sara Mauro, MD; Simone Finocchiaro, MD; Placido Mazzone, MD; Nicola Ammirabile, MD; Antonino Imbesi, MD; Carmelo Raffo, MD; Sergio Buccheri, MD; Davide Capodanno, MD, PhD.

* DG and CL contributed equally.

Division of Cardiology, Azienda Ospedaliero-Universitaria Policlinico "Rodolico – San Marco", University of Catania, Catania, Italy

TABLE OF CONTENTS

PROTOCOL REGISTRATION 6

LIST OF INCLUDED TRIALS 11

SUPPLEMENTARY METHODS 18

Frequentist and Bayesian Frameworks, Network and Pairwise Meta-Analyses 18

Search, Data Extraction, and Qualitative Assessment 18

Endpoints 20

SUPPLEMENTARY TABLES 22

Online Table 1. PRISMA-NMA. 22

Online Table 2. PRISMA-Pairwise. 26

Online Table 3. Search Strategy. 29

Online Table 4. Endpoints Across Trials. 32

Online Table 5. Sensitivity Analysis by Definition. 34

Online Table 6. Major Adverse Cardiac Events Definitions Across Included Studies. 35

Online Table 7. Main Clinical Characteristics Across Trials. 36

Online Table 8. Main Procedural Characteristics Across Trials. 39

Online Table 9. Inclusion and Exclusion Criteria Across Trials. 41

Online Table 10. Bayesian Random-Effects Network Meta-Analysis by Inconsistency Model and Comparison with Consistency Model. 55

Online Table 11. Frequentist Random-Effects Network Meta-Analysis After Excluding ILUMIEN IV. 56

Online Table 12. Bayesian Random-Effects Network Meta-Analysis After Excluding ILUMIEN IV. 58

Online Table 13. Frequentist and Bayesian Rank Probabilities and SUCRA Values After Excluding ILUMIEN IV. 60

Online Table 14. Frequentist and Bayesian Network Node Split After Excluding ILUMIEN IV. 62

Online Table 15. Frequentist Random-Effects Network Meta-Analysis After Excluding OCTOBER. 65

Online Table 16. Bayesian Random-Effects Network Meta-Analysis After Excluding OCTOBER. 67

Online Table 17. Frequentist and Bayesian Rank Probabilities and SUCRA Values After Excluding OCTOBER. 69

Online Table 18. Frequentist and Bayesian Network Node Split After Excluding OCTOBER. 71

Online Table 19. Frequentist Random-Effects Network Meta-Analysis by Definition. 74

Online Table 20. Bayesian Random-Effects Network Meta-Analysis by Definition. 76

Online Table 21. Frequentist Random-Effects Network Meta-Analysis with Outcomes Reported by IRR. 78

Online Table 22. Bayesian Random-Effects Network Meta-Analysis with Outcomes Reported by IRR. 80

Online Table 23. Frequentist Random-Effects Network Meta-Analysis at the Longest Available Follow-Up. 82

Online Table 24. Bayesian Random-Effects Network Meta-Analysis at the Longest Available Follow-Up. 84

Online Table 25. Frequentist Random-Effects Network Meta-Analysis After Excluding Trials with Higher Risk of Bias. 86

Online Table 26. Bayesian Random-Effects Network Meta-Analysis After Excluding Trials with Higher Risk of Bias. 88

Online Table 27. Frequentist Random-Effects Network Meta-Analysis After Excluding Trials Without Primary Clinical Endpoints to be Assessed at Mid- or Long-Term Follow-Up. 90

Online Table 28. Bayesian Random-Effects Network Meta-Analysis After Excluding Trials Without Primary Clinical Endpoints to be Assessed at Mid- or Long-Term Follow-Up. 92

Online Table 29. Frequentist Random-Effects Network Meta-Analysis After Excluding Trials with a Sample Size <100 Patients. 94

Online Table 30. Bayesian Random-Effects Network Meta-Analysis After Excluding Trials with a Sample Size <100 Patients. 96

Online Table 31. Frequentist Random-Effects Network Meta-Analysis After Excluding Trials Employing IVI only for Stent Optimization. 98

Online Table 32. Bayesian Random-Effects Network Meta-Analysis After Excluding Trials Employing IVI only for Stent Optimization. 100

Online Table 33. Frequentist Random-Effects Network Meta-Analysis After Excluding EROSION III. 102

Online Table 34. Frequentist and Rank Probabilities and SUCRA Values After Excluding EROSION III. 104

Online Table 35. Bayesian Random-Effects Network Meta-Analysis After Excluding EROSION III. 106

Online Table 36. Bayesian and Rank Probabilities and SUCRA Values After Excluding EROSION III. 108

Online Table 37. Bayesian Random-Effects Network Meta-Regression Analysis by Diabetes. 110

Online Table 38. Bayesian Random-Effects Network Meta-Regression Analysis by Acute Coronary Syndrome. 112

Online Table 39. Bayesian Random-Effects Network Meta-Regression Analysis by Stent Length. 114

Online Table 40. Bayesian Random-Effects Network Meta-Regression Analysis by Bifurcation Disease. 116

Online Table 41. Bayesian Random-Effects Network Meta-Regression Analysis by Chronic Total Occlusion. 118

Online Table 42. Frequentist Random-Effects Network Meta-Analysis in nonEast Asian Trials. 120

Online Table 43. Bayesian Random-Effects Network Meta-Analysis in nonEast Asian Trials. 122

Online Table 44. Frequentist Random-Effects Network Meta-Analysis in East Asian Trials. 124

Online Table 45. Bayesian Random-Effects Network Meta-Analysis in East Asian Trials. .. 126

Online Table 46. Frequentist and Bayesian Random-Effects Pairwise Meta-Analysis of Trials Comparing IVI- vs ICA-guided PCI by IRR. 128

Online Table 47. Frequentist and Bayesian Random-Effects Pairwise Meta-Analysis of Trials Comparing IVI- vs ICA-guided PCI by Definition. 129

Online Table 48. Frequentist and Bayesian Random-Effects Pairwise Meta-Analysis of Trials Comparing IVI- vs ICA-guided PCI at the Longest Available Follow-Up. 130

Online Table 49. Frequentist and Bayesian Random-Effects Pairwise Meta-Analysis of Trials Comparing IVI- vs ICA-guided PCI After Excluding Trials with Higher Risk of Bias. 131

Online Table 50. Frequentist and Bayesian Random-Effects Pairwise Meta-Analysis of Trials Comparing IVI- vs ICA-guided PCI After Excluding Trials Without Primary Clinical Endpoints to be Assessed at Mid- or Long-Term Follow-Up. 132

Online Table 51. Meta-Regression Analysis of Trials Comparing IVI- vs ICA-guided PCI. 133

Online Table 52. Frequentist and Bayesian Random-Effects Network Meta-Analysis of Trials comparing IVI- vs ICA-guided PCI in nonEast Asian Trials. 135

Online Table 53. Frequentist and Bayesian Random-Effects Network Meta-Analysis of Trials comparing IVI- vs ICA-guided PCI in East Asian Trials. 136

Online Table 54. Assessment of the Results According to GRADE. 137

SUPPLEMENTARY FIGURES 139

Online Figure 1. Flow Diagram. 139

Online Figure 2. Risk of Bias by Individual Trials. 140

Online Figure 3. Risk of Bias Across Trials. 141

Online Figure 4. Chronologic Cumulative Meta-Analyses Across Direct Comparisons for Target Lesion Revascularization and Myocardial Infarction. 142

Online Figure 5. Pairwise Direct Comparisons for Cardiac Death. 144

Online Figure 6. Pairwise Direct Comparisons for Stent Thrombosis. 145

Online Figure 7. Chronologic Cumulative Meta-Analyses Across Direct Comparisons for Ischemia-Driven Target Lesion Revascularization and Target Vessel Myocardial Infarction. 146

Online Figure 8. Chronologic Cumulative Meta-Analyses Between IVI and ICA for Target Lesion Revascularization and Myocardial Infarction. 148

Online Figure 10. Comparison-Adjusted Funnel Plots – Network Meta-Analyses. 150

Online Figure 11. Contour-Enhanced Funnel Plots – Pairwise Meta-Analyses IVI- vs ICA-guided PCI. 151

PROTOCOL REGISTRATION

NIHR | National Institute for Health Research

PROSPERO
International prospective register of systematic reviews

Coronary Angiography, Intravascular-Ultrasound, and Optical Coherence Tomography in the Guidance of Percutaneous Coronary Intervention: A Systematic Review and Network Meta-Analysis

To enable PROSPERO to focus on COVID-19 submissions, this registration record has undergone basic automated checks for eligibility and is published exactly as submitted. PROSPERO has never provided peer review, and usual checking by the PROSPERO team does not endorse content. Therefore, automatically published records should be treated as any other PROSPERO registration. Further detail is provided here.

Citation

Claudio Laudani, Davide Capodanno, Daniele Giacoppo, Antonino Imbesi, Antonio Greco, Carla Rochira, Carmelo Raffo, Davide Landolina, Giovanni Occhipinti, Marco Spagnolo, Maria Sara Mauro, Nicola Ammirabile, Placido Mazzone, Simone Finocchiaro, Sergio Buccheri. Coronary Angiography, Intravascular-Ultrasound, and Optical Coherence Tomography in the Guidance of Percutaneous Coronary Intervention: A Systematic Review and Network Meta-Analysis. PROSPERO 2023 CRD42023455920 Available from: https://www.crd.york.ac.uk/prospero/display_record.php?ID=CRD42023455920

Review question

In patients with coronary artery disease (CAD) undergoing percutaneous coronary intervention (PCI), whether systematic intravascular imaging guidance improves clinical outcomes compared with invasive coronary angiography (ICA) alone is still unclear. In addition, differences in resolution and tissue penetration, between the two main intravascular imaging technologies – intravascular ultrasound (IVUS) and optical coherence tomography (OCT) – may influence long-term clinical outcomes.

Searches

We searched available trials on ICA alone vs IVUS and/or OCT and IVUS vs OCT through major electronic databases (PubMed/MEDLINE, Scopus, Web of Science, and Cochrane Library). The search was conducted from the database inception to the search string launch date and no language restrictions were imposed. In addition, tangential exploration of the websites of the most important congresses in the field of cardiovascular medicine were screened and data from the official conference proceedings were reviewed. A final update of the search will be conducted after completing the statistical analysis.

Types of study to be included

The present review will include clinical studies comparing at least two of the prespecified strategies among ICA, IVUS, and OCT. We searched only for clinical studies satisfying the following requirements: random allocation of patients to different strategies, drug-eluting stent implantation, and long-term clinical follow-up availability. Patients of any age, gender, and clinical setting could be included.

Condition or domain being studied

Angiographically-defined obstructive CAD leading to myocardial ischaemia (ischaemic heart disease) based on clinical symptoms, electrocardiogram, echocardiography, noninvasive functional and nonfunctional test, and laboratory assessment.

Participants/population

Patients presenting with coronary artery disease requiring treatment with PCI with or without intravascular imaging guidance.

Intervention(s), exposure(s)

IVUS and OCT, defined as invasive intravascular imaging techniques based on catheters placed inside the coronary artery segments to define the morphology and compositions of coronary plaques, indicate the mechanism leading to plaque destabilisation and significant lumen obstruction, and provide guidance in the selection of the optimal device size and length. IVUS is based on ultrasound (40-mm wavelength at 40 MHz), whereas OCT uses infrared light (1.3-mm wavelength). After stenting, intravascular imaging techniques can also guide the achievement of optimal stent expansion and identify acute complications (e.g., edge dissection, stent malapposition, tissue protrusion, etc.).

Comparator(s)/control

ICA, defined as the use of catheters to engage the left and right coronary arteries and selectively infuse contrast dye to evaluate the presence of coronary stenoses. Visual information of ICA can be implemented by quantitative coronary angiography, a software-based assessment that quantifies coronary diameter stenosis and length in relation to a known reference diameter, usually the guiding catheter.

Context

The present study will summarize available evidence of revascularization through PCI using ICA, IVUS or OCT to guide or optimize stent implantation.

Main outcome(s)

The main outcome of the present study will be target vessel revascularization. The co-primary outcome will be myocardial infarction.

Measures of effect

Comparisons between PCI guidance strategies will be reported by odds ratios and 95% confidence intervals.

Additional outcome(s)

- All-cause death

- Cardiac death

- Target vessel myocardial infarction

- Target vessel revascularization

-Composite endpoint of major cardiovascular events, primarily cardiac death, target vessel myocardial infarction, or target lesion revascularization followed by a composite of cardiac death, target vessel myocardial infarction, or target vessel revascularization

Measures of effect

Comparisons between PCI guidance strategies will be reported by odds ratios and 95% confidence intervals.

Data extraction (selection and coding)

After running the search queries, titles and abstracts will be independently screened by six reviewers to identify trials meeting the inclusion criteria. After removing duplicate entries identified through searches across various electronic databases, the retrieved reports will undergo an independent full-text screening process by six reviewers to confirm compliance with the eligibility criteria. Different reports pertaining to the same trial (e.g., analyses at a different follow-up time) were combined. After the conclusion of each independent review process, the results will collegially be reviewed to define the final pool of includable trials. Data on the outcomes of interest and main clinical and procedural characteristics will be extracted at arm level and included in the dedicated electronic spreadsheets. Trial-level information, including the main characteristics of the design, follow-up duration, definitions, as well as inclusion and inclusion criteria will be summarized.

Risk of bias (quality) assessment

After including the trials in the database, three authors independently evaluated the individual risk of bias by using the second version of the Cochrane risk-of-bias tool for randomized trials (RoB 2). Disagreements were solved by consensus under the supervision of two senior authors. Publication bias will be complemented by the graphical assessment of comparison-adjusted funnel plots and Egger's regression test.

Strategy for data synthesis

The statistical analysis will be based on random-effects frequentist network meta-analysis to compute odds ratios and 95% confidence intervals for each outcome of interest. The primary analysis will be focused on long-term outcomes, whereas further analyses will be performed to evaluate the effects of each strategy on both short- and very long-term events. The network metanalyses will be replicated by Bayesian random-effect models. Pairwise comparisons (i.e., direct evidence) between strategies will be provided as well as a comprehensive pairwise random-effects meta-analysis comparing ICA vs intravascular imaging, regardless of the technique employed (IVUS or OCT). A leave-one-out analysis will be performed for further evaluation of each trial weight on the overall effect-size estimation for each endpoint. The heterogeneity will be assessed by Cochrane's Q statistic, $\tau 2$ and I^2 statistics. Inconsistency will be evaluated by node-split analyses. All the analyses will be performed with R (version 4.0.5) and STATA (version 13.2).

Analysis of subgroups or subsets

The analyses of the prespecified endpoints will be repeated in the sequent subgroups:

· After the exclusion of trials focusing on acute coronary syndrome patients

· Including only trials focusing on acute coronary syndrome

· After exclusion of trials focusing on complex lesions (i.e., bifurcation, coronary total occlusion, left main)

· Analyses restricted to studies with >100 patients

· After the exclusion of trials focusing on Asian patients

· Including only trials focusing on Asian patients

Contact details for further information

Claudio Laudani

claudani313@gmail.com

Organisational affiliation of the review

Università degli studi di Catania

Review team members and their organisational affiliations

Dr Claudio Laudani. UniversitÃ degli studi di Catania

Professor Davide Capodanno. Università degli studi di Catania

Dr Daniele Giacoppo. Università degli studi di Catania

Dr Antonino Imbesi. Unversità degli studi di Catania

Dr Antonio Greco. Università degli studi di Catania

Dr Carla Rochira. Università degli studi di Catania

Dr Carmelo Raffo. Università degli studi di Catania

Dr Davide Landolina. Università degli studi di Catania

Dr Giovanni Occhipinti. Università degli studi di Catania

Dr Marco Spagnolo. Università degli studi di Catania

Dr Maria Sara Mauro. Università degli studi di Catania

Dr Nicola Ammirabile. Università degli studi di Catania

Dr Placido Mazzone. Università degli studi di Catania

Dr Simone Finocchiaro. Università degli studi di Catania

Dr Sergio Buccheri. Uppsala University

Type and method of review

Meta-analysis, Network meta-analysis, Prognostic, Systematic review

Anticipated or actual start date

01 July 2023

Anticipated completion date

06 November 2023

Funding sources/sponsors

None.

Conflicts of interest

Language

English

Country

Italy

Stage of review

Review Ongoing

Subject index terms status

Subject indexing assigned by CRD

Subject index terms

MeSH headings have not been applied to this record

Date of registration in PROSPERO

30 August 2023

Date of first submission

19 August 2023

Stage of review at time of this submission

Stage	Started	Completed
Preliminary searches	Yes	Yes
Piloting of the study selection process	Yes	Yes
Formal screening of search results against eligibility criteria	Yes	No
Data extraction	No	No
Risk of bias (quality) assessment	No	No
Data analysis	No	No

The record owner confirms that the information they have supplied for this submission is accurate and complete and they understand that deliberate provision of inaccurate information or omission of data may be construed as scientific misconduct.

The record owner confirms that they will update the status of the review when it is completed and will add publication details in due course.

Versions

30 August 2023

LIST OF INCLUDED TRIALS

- **AIR-CTO (2015)**

Nai-Liang Tian, Sandeep-Kumar Gami, Fei Ye, Jun-Jie Zhang, Zhi-Zhong Liu, Song Lin, Zhen Ge, Shou-Jie Shan, Wei You, Liang Chen, Yao-Jun Zhang, Gary Mintz, Shao-Liang Chen.

Angiographic and clinical comparisons of intravascular ultrasound- versus angiography-guided drug-eluting stent implantation for patients with chronic total occlusion lesions: two-year results from a randomised AIR-CTO study.

EuroIntervention 2015;10:1409-17. doi:10.4244/EIJV10I12A245.

- **AVIO (2013)**

Alaide Chieffo, Azeem Latib, Christophe Caussin, Patrizia Presbitero, Stefano Galli, Alberto Menozzi, Ferdinando Varbella, Fina Mauri, Marco Valgimigli, Chourmouzios Arampatzis, Manuel Sabate, Andrejs Erglis, Bernhard Reimers, Flavio Airoldi, Mika Laine, Ramon Lopez Palop, Ghada Mikhail, Philip Maccarthy, Francesco Romeo, Antonio Colombo.

A prospective, randomized trial of intravascular-ultrasound guided compared to angiography guided stent implantation in complex coronary lesions: the AVIO trial.

Am Heart J 2013;165:65-72. doi:10.1016/j.ahj.2012.09.017.

- **CTO-IVUS (2015)**

Byeong-Keuk Kim, Dong-Ho Shin, Myeong-Ki Hong, Hun Sik Park, Seung-Woon Rha, Gary S Mintz, Jung-Sun Kim, Je Sang Kim, Seung-Jin Lee, Hee-Yeol Kim, Bum-Kee Hong, Woong-Chol Kang, Jin-Ho Choi, Yangsoo Jang.

Clinical Impact of Intravascular Ultrasound-Guided Chronic Total Occlusion Intervention With Zotarolimus-Eluting Versus Biolimus-Eluting Stent Implantation: Randomized Study.

Circ Cardiovasc Interv 2015;8:e002592. doi:10.1161/CIRCINTERVENTIONS.115.002592.

- **DOCTORS (2016)**

Nicolas Meneveau, Geraud Souteyrand, Pascal Motreff, Christophe Caussin, Nicolas Amabile, Patrick Ohlmann, Olivier Morel, Yoann Lefrançois, Vincent Descotes-Genon, Johanne Silvain, Nassim Braik, Romain Chopard, Marion Chatot, Fiona Ecarnot, Hélène Tauzin, Eric Van Belle, Loïc Belle, François Schiele.

Optical Coherence Tomography to Optimize Results of Percutaneous Coronary Intervention in Patients with Non-ST-Elevation Acute Coronary Syndrome: Results of the Multicenter, Randomized DOCTORS Study (Does Optical Coherence Tomography Optimize Results of Stenting).

Circulation 2016;134:906-17. doi:10.1161/CIRCULATIONAHA.116.024393.

- **EROSION III (2022)**

Haibo Jia, Jiannan Dai, Luping He, Yishuo Xu, Yongfeng Shi, Lei Zhao, Zhiqi Sun, Yin Liu, Ziqian Weng, Xue Feng, Dirui Zhang, Tao Chen, Xiling Zhang, Lulu Li, Yousheng Xu, Yanqing Wu, Yining Yang, Chunmei Wang, Lang Li, Jianping Li, Jingbo Hou, Bin Liu0, Gary S Mintz, Bo Yu.

EROSION III: A Multicenter RCT of OCT-Guided Reperfusion in STEMI With Early Infarct Artery Patency.

JACC Cardiovasc Interv 2022;15:846-856. doi:10.1016/j.jcin.2022.01.298.

- **GUIDE-DES (2023)**

Pil Hyung Lee, Soon Jun Hong, Hyun-Sook Kim, Young Won Yoon, Jong-Young Lee, Seung-Jin Oh, Soo-Jin Kang, Young-Hak Kim, Seong-Wook Park, Seung-Whan Lee, Cheol Whan Lee.

Quantitative coronary angiography versus intravascular ultrasound guidance for drug-eluting stent implantation (GUIDE-DES): study protocol for a randomised controlled non-inferiority trial.

BMJ Open 2022;12:e052215. doi:10.1136/bmjopen-2021-052215.

- **HOME DES IVUS (2010)**

Jozef Jakabcin, Radim Spacek, Marian Bystron, Martin Kvasnák, Jiri Jager, Josef Veselka, Petr Kala, Pavel Cervinka.

Long-term health outcome and mortality evaluation after invasive coronary treatment using drug eluting stents with or without the IVUS guidance. Randomized control trial. HOME DES IVUS.

Catheter Cardiovasc Interv 2010;75:578-83. doi:10.1002/ccd.22244.

- **ILUMIEN III (2016)**

Ziad A Ali, Akiko Maehara, Philippe Généreux, Richard A Shlofmitz, Franco Fabbiocchi, Tamim M Nazif, Giulio Guagliumi, Perwaiz M Meraj 6, Fernando Alfonso, Habib Samady, Takashi Akasaka, Eric B Carlson, Massoud A Leesar, Mitsuaki Matsumura, Melek Ozgu Ozan, Gary S Mintz, Ori Ben-Yehuda, Gregg W Stone.

Optical coherence tomography compared with intravascular ultrasound and with angiography to guide coronary stent implantation (ILUMIEN III: OPTIMIZE PCI): a randomised controlled trial.

Lancet 2016;388:2618-2628. doi:10.1016/S0140-6736(16)31922-5.

Ziad A Ali, Keyvan Karimi Galougahi, Akiko Maehara, Richard A Shlofmitz, Franco Fabbiocchi, Giulio Guagliumi, Fernando Alfonso, Takashi Akasaka, Mitsuaki Matsumura, Gary S Mintz, Ori Ben-Yehuda, Zhen Zhang, Richard R Rapoza, Nick E J West, Gregg W Stone.

Outcomes of optical coherence tomography compared with intravascular ultrasound and with angiography to guide coronary stent implantation: one-year results from the ILUMIEN III: OPTIMIZE PCI trial.

EuroIntervention 2021;16:1085-1091. doi:10.4244/EIJ-D-20-00498.

- **ILUMIEN IV (2023)**

Ziad Ali, Ulf Landmesser, Keyvan Karimi Galougahi, Akiko Maehara, Mitsuaki Matsumura, Richard A Shlofmitz, Giulio Guagliumi, Matthew J Price, Jonathan M Hill, Takashi Akasaka, Francesco Prati, Hiram G Bezerra, William Wijns, Gary S Mintz, Ori Ben-Yehuda, Robert J McGreevy, Zhen Zhang, Richard R Rapoza, Nick E J West, Gregg W Stone.

Optical coherence tomography-guided coronary stent implantation compared to angiography: a multicentre randomised trial in PCI - design and rationale of ILUMIEN IV: OPTIMAL PCI.

EuroIntervention 2021;16:1092-1099. doi:10.4244/EIJ-D-20-00501.

Ziad A Ali, Ulf Landmesser, Akiko Maehara, Mitsuaki Matsumura, Richard A Shlofmitz, Giulio Guagliumi, Matthew J Price, Jonathan M Hill, Takashi Akasaka, Francesco Prati, Hiram G Bezerra, William Wijns, David Leistner, Paolo Canova, Fernando Alfonso, Franco Fabbiocchi, Ozgen Dogan, Robert J McGreevy, Robert W McNutt, Hong Nie, Jana Buccola, Nick E J West, Gregg W Stone.

Optical Coherence Tomography-Guided versus Angiography-Guided PCI.

N Engl J Med 2023;389:1466-1476. doi:10.1056/NEJMoa2305861.

• iSIGHT (2021)

Daniel Chamié, J Ribamar Costa Jr, Lucas P Damiani, Dimytri Siqueira, Sérgio Braga, Ricardo Costa, Henry Seligman, Freddy Brito, Guilherme Barreto, Rodolfo Staico, Fausto Feres, Ricardo Petraco , Alexandre Abizaid.

Optical Coherence Tomography Versus Intravascular Ultrasound and Angiography to Guide Percutaneous Coronary Interventions: The iSIGHT Randomized Trial.

Circ Cardiovasc Interv 2021;14:e009452. doi:10.1161/CIRCINTERVENTIONS.120.009452.

• IVUS-XPL (2015)

Sung-Jin Hong, Byeong-Keuk Kim, Dong-Ho Shin, Chung-Mo Nam, Jung-Sun Kim, Young-Guk Ko, Donghoon Choi, Tae-Soo Kang, Woong-Chol Kang, Ae-Young Her, Yong Hoon Kim, Seung-Ho Hur, Bum-Kee Hong, Hyuckmoon Kwon, Yangsoo Jang, Myeong-Ki Hong.

Effect of Intravascular Ultrasound-Guided vs Angiography-Guided Everolimus-Eluting Stent Implantation: The IVUS-XPL Randomized Clinical Trial.

JAMA 2015;314:2155-63. doi:10.1001/jama.2015.15454.

Sung-Jin Hong, Gary S Mintz, Chul-Min Ahn, Jung-Sun Kim, Byeong-Keuk Kim, Young-Guk Ko, Tae-Soo Kang, Woong-Chol Kang, Yong Hoon Kim, Seung-Ho Hur, Bum-Kee Hong, Donghoon Choi, Hyuckmoon Kwon, Yangsoo Jang, Myeong-Ki Hong.

Effect of Intravascular Ultrasound-Guided Drug-Eluting Stent Implantation: 5-Year Follow-Up of the IVUS-XPL Randomized Trial.

JACC Cardiovasc Interv 2020;13:62-71. doi:10.1016/j.jcin.2019.09.033.

- **Kala et al. (2018)**

  Petr Kala, Pavel Cervinka, Martin Jakl, Jan Kanovsky, Andrej Kupec, Radim Spacek, Martin Kvasnak, Martin Poloczek, Michaela Cervinkova, Hiram Bezerra, Zdenek Valenta, Guilherme F Attizzani, Audrey Schnell, Lu Hong, Marco A Costa.

  OCT guidance during stent implantation in primary PCI: A randomized multicenter study with nine months of optical coherence tomography follow-up.

  Int J Cardiol 2018;250:98-103. doi:10.1016/j.ijcard.2017.10.059.

- **Kim et al. (2015)**

  Jung-Sun Kim, Dong-Ho Shin, Byeong-Keuk Kim, Young-Guk Ko, Donghoon Choi, Yangsoo Jang, Myeong-Ki Hong.

  Randomized comparison of stent strut coverage following angiography- or optical coherence tomography-guided percutaneous coronary intervention.

  Rev Esp Cardiol 2015;68:190-7. doi:10.1016/j.rec.2014.07.025.

- **Li et al. (2019)**

  Lin Li, Li Wang, Chun-Juan Zhai, Ya-Ru Mou, Jian-Hong Wang, Lian-Qun Cui.

  Clinical utility of intravascular ultrasonography-guided therapy in a small-vessel coronary lesion associated with Type 2 diabetes mellitus.

  Anatol J Cardiol 2019;22:68-76. doi:10.14744/AnatolJCardiol.2019.77009.

- **Liu et al. (2019)**

  Xiao Ming Liu, Zuo Ming Yang, Xiao Kun Liu, Qi Zhang, Chang Qing Liu, Quan Le Han, Jian Hua Sun.

  Intravascular ultrasound-guided drug-eluting stent implantation for patients with unprotected left main coronary artery lesions: A single-center randomized trial.

  Anatol J Cardiol 2019;21:83-90. doi:10.14744/AnatolJCardiol.2018.21447.

- **MISTIC-1 (2020)**

  Takashi Muramatsu, Yukio Ozaki, Mamoru Nanasato, Masato Ishikawa, Ryo Nagasaka, Masaya Ohota, Yosuke Hashimoto, Yu Yoshiki, Hidemaro Takatsu, Katsuyoshi Ito, Hiroki Kamiya, Yukihiko Yoshida, Toyoaki Murohara, Hideo Izawa.

  Comparison Between Optical Frequency Domain Imaging and Intravascular Ultrasound for Percutaneous Coronary Intervention Guidance in Biolimus A9-Eluting Stent Implantation: A Randomized MISTIC-1 Non-Inferiority Trial.

  Circ Cardiovasc Interv 2020;13:e009314. doi:10.1161/CIRCINTERVENTIONS.120.009314.

- **OCTACS (2015)**

Lisbeth Antonsen, Per Thayssen, Akiko Maehara, Henrik Steen Hansen, Anders Junker, Karsten Tange Veien, Knud Nørregaard Hansen, Mikkel Hougaard, Gary S Mintz, Lisette Okkels Jensen.

Optical Coherence Tomography Guided Percutaneous Coronary Intervention With Nobori Stent Implantation in Patients With Non-ST-Segment-Elevation Myocardial Infarction (OCTACS) Trial: Difference in Strut Coverage and Dynamic Malapposition Patterns at 6 Months.

Circ Cardiovasc Interv 2015;8:e002446. doi:10.1161/CIRCINTERVENTIONS.114.002446.

- **OCTIVUS (2023)**

Do-Yoon Kang, Jung-Min Ahn, Hanbit Park, Pil Hyung Lee, Soo-Jin Kang, Seung-Whan Lee, Young-Hak Kim, Seong-Wook Park, Sang-Wook Kim, Seung-Ho Hur, Yun-Kyeong Cho, Cheol Hyun Lee, Soon Jun Hong, Young Joon Hong, Young Won Yoon, Soo-Joong Kim, Jang-Ho Bae, Jun-Hyok Oh, Duk-Woo Park, Seung-Jung Park.

Comparison of optical coherence tomography-guided versus intravascular ultrasound-guided percutaneous coronary intervention: Rationale and design of a randomized, controlled OCTIVUS trial.

Am Heart J 2020:228:72-80. doi:10.1016/j.ahj.2020.08.003.

Do-Yoon Kang, Jung-Min Ahn, Sung-Cheol Yun, Seung Ho Hur, Yun-Kyeong Cho, Cheol Hyun Lee, Soon Jun Hong, Subin Lim, Sang-Wook Kim, Hoyoun Won, Jun-Hyok Oh, Jeong Cheon Choe, Young Joon Hong, Yong-Hoon Yoon, Hoyun Kim, Yeonwoo Choi, Jinho Lee, Young Won Yoon, Soo-Joong Kim, Jang Ho Bae, Duk-Woo Park, Seung-Jung Park.

Optical Coherence Tomography-Guided or Intravascular Ultrasound Guided Percutaneous Coronary Intervention: The OCTIVUS Randomized Clinical Trial.

Circulation 2023;148:1195-1206. doi:10.1161/CIRCULATIONAHA.123.066429.

- **OCTOBER (2023)**

Niels Ramsing Holm, Lene Nyhus Andreasen, Simon Walsh, Olli A Kajander, Nils Witt, Christian Eek, Paul Knaapen, Lukasz Koltowski, Juan Luis Gutiérrez-Chico, Francesco Burzotta, Janusz Kockman, John Ormiston, Irene Santos-Pardo, Peep Laanmets, Darren Mylotte, Morten Madsen, Jakob Hjort, Indulis Kumsars, Truls Råmunddal, Evald Høj Christiansen.

Rational and design of the European randomized Optical Coherence Tomography Optimized Bifurcation Event Reduction Trial (OCTOBER).

Am Heart J 2018:205:97-109. doi:10.1016/j.ahj.2018.08.003.

Niels R Holm, Lene N Andreasen, Omeed Neghabat, Peep Laanmets, Indulis Kumsars, Johan Bennett, Niels T Olsen, Jacob Odenstedt, Pavel Hoffmann, Jo Dens, Saqib Chowdhary, Peter O'Kane, Søren-Haldur Bülow Rasmussen, Matthias Heigert, Ole Havndrup, Jan P Van Kuijk, Simone Biscaglia, Lone J H Mogensen, Loghman Henareh, Francesco Burzotta, Christian H Eek, Darren Mylotte, Miquel S Llinas, Lukasz Koltowski, Paul Knaapen, Slobodan Calic, Nils Witt, Irene Santos-Pardo, Stuart Watkins, Jacob Lønborg, Andreas T Kristensen, Lisette O

Jensen, Fredrik Calais, James Cockburn, Andrew McNeice, Olli A Kajander, Ton Heestermans, Stephan Kische, Ashkan Eftekhari, James C Spratt, Evald H Christiansen.

OCT or Angiography Guidance for PCI in Complex Bifurcation Lesions.

N Engl J Med 2023;389:1477-1487. doi:10.1056/NEJMoa2307770.

- **OPINION (2017)**

Takashi Kubo, Toshiro Shinke, Takayuki Okamura, Kiyoshi Hibi, Gaku Nakazawa, Yoshihiro Morino, Junya Shite, Tetsuya Fusazaki, Hiromasa Otake, Ken Kozuma, Takashi Akasaka.

Optical frequency domain imaging vs. intravascular ultrasound in percutaneous coronary intervention (OPINION trial): Study protocol for a randomized controlled trial

J Cardiol 2016;68:455-460. doi:10.1016/j.jjcc.2015.11.007.

Takashi Kubo, Toshiro Shinke, Takayuki Okamura, Kiyoshi Hibi, Gaku Nakazawa, Yoshihiro Morino, Junya Shite, Tetsuya Fusazaki, Hiromasa Otake, Ken Kozuma, Tetsuya Ioji, Hideaki Kaneda, Takeshi Serikawa, Toru Kataoka, Hisayuki Okada, Takashi Akasaka.

Optical frequency domain imaging vs. intravascular ultrasound in percutaneous coronary intervention (OPINION trial): one-year angiographic and clinical results.

Eur Heart J 2017;38:3139–3147. doi:10.1093/eurheartj/ehx351.

- **RENOVATE-COMPLEX-PCI (2023)**

Joo Myung Lee, Ki Hong Choi, Young Bin Song, Jong-Young Lee, Seung-Jae Lee, Sang Yeub Lee, Sang Min Kim, Kyeong Ho Yun, Jae Young Cho, Chan Joon Kim, Hyo-Suk Ahn, Chang-Wook Nam, Hyuck-Jun Yoon, Yong Hwan Park, Wang Soo Lee, Jin-Ok Jeong, Pil Sang Song, Joon-Hyung Doh, Sang-Ho Jo, Chang-Hwan Yoon, Min Gyu Kang, Jin-Sin Koh, Kwan Yong Lee, Young-Hyo Lim, Yun-Hyeong Cho, Jin-Man Cho, Woo Jin Jang, Kook-Jin Chun, David Hong, Taek Kyu Park, Jeong Hoon Yang, Seung-Hyuk Choi, Hyeon-Cheol Gwon, Joo-Yong Hahn.

Intravascular Imaging-Guided or Angiography-Guided Complex PCI.

N Engl J Med 2023;388:1668-1679. doi:10.1056/NEJMoa2216607.

- **RESET (2013)**

Jung-Sun Kim, Tae-Soo Kang, Gary S. Mintz, Byoung-Eun Park, Dong-Ho Shin, Byeong-Keuk Kim, Young-Guk Ko, Donghoon Choi, Yangsoo Jang, Myeong-Ki Hong.

Randomized Comparison of Clinical Outcomes Between Intravascular Ultrasound and Angiography-Guided Drug-Eluting Stent Implantation for Long Coronary Artery Stenoses.

JACC Cardiovasc Interv 2013;6:369-76. doi:10.1016/j.jcin.2012.11.009.

- **Tan et al. (2015)**

Qiang Tan, Qingsheng Wang, Dongtian Liu, Shuangyue Zhang, Yang Zhang, Yang Li.

Intravascular ultrasound-guided unprotected left main coronary artery stenting in the elderly.

Saudi Med J 2015;36:549-553. doi:10.15537/smj.2015.5.11251.

- **ULTIMATE (2018)**

Junjie Zhang, Xiaofei Gao, Jing Kan, Zhen Ge, Leng Han, Shu Lu, Nailiang Tian, Song Lin, Qinghua Lu, Xueming Wu, Qihua Li, Zhizhong Liu, Yan Chen, Xuesong Qian, Juan Wang, Dayang Chai, Chonghao Chen, Xiaolong Li, Bill D Gogas, Tao Pan, Shoujie Shan, Fei Ye, Shao-Liang Chen.

Intravascular Ultrasound Versus Angiography-Guided Drug-Eluting Stent Implantation: The ULTIMATE Trial.

J Am Coll Cardiol 2018;72:3126-3137. doi:10.1016/j.jacc.2018.09.013.

Xiao-Fei Gao, Zhen Ge, Xiang-Quan Kong, Jing Kan, Leng Han, Shu Lu, Nai-Liang Tian, Song Lin, Qing-Hua Lu, Xiao-Yan Wang, Qi-Hua Li, Zhi-Zhong Liu, Yan Chen, Xue-Song Qian, Juan Wang, Da-Yang Chai, Chong-Hao Chen, Tao Pan, Fei Ye, Jun-Jie Zhang, Shao-Liang Chen.

3-Year Outcomes of the ULTIMATE Trial Comparing Intravascular Ultrasound Versus Angiography-Guided Drug-Eluting Stent Implantation.

JACC Cardiovasc Interv 2021;14:247-257. doi:10.1016/j.jcin.2020.10.001.

- **Wang et al. (2015)**

Hong-Xia Wang, Ping-Shuan Dong, Zhi-Juan Li, Hong-Lei Wang, Ke Wang, Xiang-Yong Liu.

Application of Intravascular Ultrasound in the Emergency Diagnosis and Treatment of Patients with ST-Segment Elevation Myocardial Infarction.

Echocardiography 2015;32:1003-8. doi:10.1111/echo.12794.

SUPPLEMENTARY METHODS

Frequentist and Bayesian Frameworks, Network and Pairwise Meta-Analyses

Analyses were conducted in the frequentist framework and replicated in the Bayesian framework.[9,16] The frequentist method operates by assessing the probability of significance and a 95% confidence interval (CI) leading to the acceptance or rejection of a research hypothesis.[9,16] In contrast, the Bayesian method computes the posterior probability of a research hypothesis by integrating the information inherent in the data with the prior probability derived from previously known information.[9,16] In Bayesian analyses, summary estimates are reported along with 95% credible intervals (CrIs).[9,16] However, the CrI presents different definition and meaning compared with CI since it is the range containing a particular percentage (i.e., usually 95%) of the posterior probable values.[9,16] Bayesian models based on noninformative overdispersed priors generally produce more conservative results compared with frequentist models.[9,16]

In network meta-analyses, treatment estimates result from the combination of the direct evidence deriving from the head-to-head comparison (i.e., direct connection in the network) with the indirect evidence deriving from the network.[9,13] In contrast, pairwise meta-analyses compare two treatments at a time and rely only on direct evidence (i.e., direct comparison of the two treatments).[9]

Search, Data Extraction, and Qualitative Assessment

Trials comparing invasive coronary angiography (ICA) alone, intravascular ultrasound (IVUS), and optical coherence tomography (OCT) to guide percutaneous coronary intervention (PCI) were searched across major electronic databases (PubMed/Medline, Scopus, Web of Science, Cochrane Library). The search spanned from the date of the inception of each database to the date of each search string deployment. No language restrictions were imposed. Additionally, a supplementary

search on the websites of leading cardiovascular medicine conferences and major cardiovascular scientific societies was conducted to review data from official conference proceedings and gather any pertinent news regarding potentially relevant trials. Following the execution of search queries, six reviewers independently screened titles and abstracts to identify trials that met the inclusion criteria. Duplicate entries identified across various electronic databases were removed. The remaining reports underwent independent full-text screening by the same six reviewers to confirm compliance with the eligibility criteria. Reports related to the same trial, such as analyses at different follow-up times, were integrated. After completing each independent review process, the results were collectively reviewed to determine the final pool of trials that met the inclusion criteria. Discordant results were solved by consensus under the supervision of the lead investigators. Data pertaining to the outcomes of interest, as well as the primary clinical and procedural characteristics, were extracted at the arm level and incorporated into dedicated electronic spreadsheets. Key trial-level data, encompassing design features, follow-up duration, definitions, as well as inclusion and exclusion criteria, were extensively summarized. Before running the statistical analysis, the reviewers collegially assessed the quality of each trial by using the Risk of Bias (RoB) 2.[10] The Risk of Bias 2.0 is a qualitative grading system comprising five prespecified domains: 1. bias arising from the randomization process; 2. bias due to deviations from intended interventions; 3. bias due to missing outcome data; 4. bias in measurement of the outcome; and 5. bias in selection of the reported result.[10] Each domain is based on a series of "signaling questions", a judgment about risk of bias for the domains, one or more justification responses to the signaling questions and risk-of-bias judgements, and an option to explain the likely direction of bias.[10] The risk of bias judgement implies the assignment of one of three levels to each domain: low risk of bias, some concerns, or high risk of bias.[10] The adjudication algorithm includes the assessment of some conditions for each

of the domains.[10] The posterior qualitative assessment of the meta-analysis results by using the Grading of Recommendations, Assessment, Development, and Evaluations (GRADE) was planned.[11]

Endpoints

Before conducting the meta-analysis, we selected the primary outcome of target lesion revascularization for two reasons. Firstly, this outcome had the potential to comprehensively encompass the effects directly attributable to IVI guidance for PCI. Indeed, at long-term follow-up, the major consequences of suboptimal PCI are in-stent restenosis and stent thrombosis, both of which generally require target lesion revascularization. Secondly, target lesion revascularization ranked among the lesion-related outcomes with the highest incidence at follow-up across known available trials. However, considering that target lesion revascularization could be less significantly influence long-term prognosis, we prespecified myocardial infarction as the coprimary endpoint. We opted for myocardial infarction instead of the preferable target lesion myocardial infarction due to our awareness that several early trials did not include the lesion-specific outcome.

After preliminary review of reported outcomes across trials, some inconsistencies in the definitions became apparent (**Online Tables 4-6**). Hence, it was opted to conduct the primary analysis with an allowance of a certain degree of heterogeneity (e.g., myocardial infarction instead of target vessel myocardial infarction) when a limited proportion of trials supplied to avoid differences across outcomes driven by the inconsistent inclusion of trials rather than true effects. Thus, for example, the analysis of myocardial infarction included a minor proportion of trials reporting only target vessel myocardial infarction and, conversely, some trials reporting only myocardial infarction were pooled in the analysis of target vessel myocardial infarction (**Online Tables 4-6**). However, in order

to avoid spurious conclusions, it was also decided to repeat the analysis of each outcome in the more restricted pool of trials employing consistent definition or mildly inconsistent definitions (**Online Tables 4-6**). In general, we defined as consistent those outcomes with the same definition, mildly inconsistent those outcomes with reasonable differences (i.e., cardiovascular death instead of cardiac death, target lesion revascularization instead of ischemia-driven target lesion revascularization, clinically-driven target lesion revascularization instead of ischemia-driven target lesion revascularization), and moderately inconsistent those outcomes with more pronounced differences (i.e., cardiac death instead of all-cause death; target vessel myocardial infarction target vessel revascularization instead of target lesion revascularization; any stent thrombosis instead of definite or probable stent thrombosis) (**Online Tables 4-6**). Although a composite of major adverse cardiac events was the primary endpoint in the several original trials, it was expected extreme inconsistency in the definition with the resulting impossibility to meaningfully combine the data. In this case the primary outcome of trials was defined as severely inconsistent. For this reason, we decided to refrain from centering our meta-analysis on a composite endpoint of major adverse cardiac events.

The preferential follow-up time was 24 months, consistently with recent pivotal trials; when this follow-up time was not available, the closest follow-up time <24 months was used. As described in the manuscript, sensitivity analyses accounting for difference in follow-up length were employed.

SUPPLEMENTARY TABLES

Online Table 1. PRISMA-NMA.

Section / Topic	Item #	Checklist Item	Page #
**TITLE**			
Title	1	Identify the report as a systematic review *incorporating a network meta-analysis (or related form of meta-analysis).*	1
**ABSTRACT**			
Structured summary	2	Provide a structured summary including, as applicable:<br>**Background:** main objectives<br>**Methods:** data sources; study eligibility criteria, participants, and interventions; study appraisal; and *synthesis methods, such as network meta-analysis.*<br>**Results:** number of studies and participants identified; summary estimates with corresponding confidence/credible intervals; *treatment rankings may also be discussed. Authors may choose to summarize pairwise comparisons against a chosen treatment included in their analyses for brevity.*<br>**Discussion/Conclusions:** limitations; conclusions and implications of findings.<br>**Other:** primary source of funding; systematic review registration number with registry name.	2-3
**INTRODUCTION**			
Rationale	3	Describe the rationale for the review in the context of what is already known*, including mention of why a network meta-analysis has been conducted.*	5-6
Objectives	4	Provide an explicit statement of questions being addressed, with reference to participants, interventions, comparisons, outcomes, and study design (PICOS).	6
**METHODS**			
Protocol and registration	5	Indicate whether a review protocol exists and if and where it can be accessed (e.g., Web address); and, if available, provide registration information, including registration number.	6, S5–S9
Eligibility criteria	6	Specify study characteristics (e.g., PICOS, length of follow-up) and report characteristics (e.g., years considered, language, publication status) used as criteria for eligibility, giving rationale. *Clearly describe eligible treatments included in the treatment network, and note whether any have been clustered or merged into the same node (with justification).*	7
Information sources	7	Describe all information sources (e.g., databases with dates of coverage, contact with study authors to identify additional	7, S16–S17

		studies) in the search and date last searched.	
Search	8	Present full electronic search strategy for at least one database, including any limits used, such that it could be repeated.	S27–S29
Study selection	9	State the process for selecting studies (i.e., screening, eligibility, included in systematic review, and, if applicable, included in the meta-analysis).	7, S27–S29
Data collection process	10	Describe method of data extraction from reports (e.g., piloted forms, independently, in duplicate) and any processes for obtaining and confirming data from investigators.	S16–S17
Data items	11	List and define all variables for which data were sought (e.g., PICOS, funding sources) and any assumptions and simplifications made.	7, S16–S18
**Geometry of the network**	**S1**	Describe methods used to explore the geometry of the treatment network under study and potential biases related to it. This should include how the evidence base has been graphically summarized for presentation, and what characteristics were compiled and used to describe the evidence base to readers.	S16–S19
Risk of bias within individual studies	12	Describe methods used for assessing risk of bias of individual studies (including specification of whether this was done at the study or outcome level), and how this information is to be used in any data synthesis.	S17
Summary measures	13	State the principal summary measures (e.g., risk ratio, difference in means). *Also describe the use of additional summary measures assessed, such as treatment rankings and surface under the cumulative ranking curve (SUCRA) values, as well as modified approaches used to present summary findings from meta-analyses.*	7, S17–S18
Planned methods of analysis	14	Describe the methods of handling data and combining results of studies for each network meta-analysis. This should include, but not be limited to:<br>• *Handling of multi-arm trials;*<br>• *Selection of variance structure;*<br>• *Selection of prior distributions in Bayesian analyses; and*<br>• *Assessment of model fit.*	7–9, S18
**Assessment of Inconsistency**	**S2**	Describe the statistical methods used to evaluate the agreement of direct and indirect evidence in the treatment network(s) studied. Describe efforts taken to address its presence when found.	8
Risk of bias across studies	15	Specify any assessment of risk of bias that may affect the cumulative evidence (e.g., publication bias, selective reporting within studies).	S17
Additional analyses	16	Describe methods of additional analyses if done, indicating which were pre-specified. This may include, but not be limited to, the following:<br>• Sensitivity or subgroup analyses;	S18–S19

		• Meta-regression analyses;<br>• *Alternative formulations of the treatment network; and*<br>• *Use of alternative prior distributions for Bayesian analyses (if applicable).*	
**RESULTS†**			
Study selection	17	Give numbers of studies screened, assessed for eligibility, and included in the review, with reasons for exclusions at each stage, ideally with a flow diagram.	9, S10–S15, S129
**Presentation of network structure**	**S3**	Provide a network graph of the included studies to enable visualization of the geometry of the treatment network.	9, S130, central illustration
**Summary of network geometry**	**S4**	Provide a brief overview of characteristics of the treatment network. This may include commentary on the abundance of trials and randomized patients for the different interventions and pairwise comparisons in the network, gaps of evidence in the treatment network, and potential biases reflected by the network structure.	9
Study characteristics	18	For each study, present characteristics for which data were extracted (e.g., study size, PICOS, follow-up period) and provide the citations.	Table 1, S30–S52
Risk of bias within studies	19	Present data on risk of bias of each study and, if available, any outcome level assessment.	S131, S132
Results of individual studies	20	For all outcomes considered (benefits or harms), present, for each study: 1) simple summary data for each intervention group, and 2) effect estimates and confidence intervals. *Modified approaches may be needed to deal with information from larger networks.*	Figures 2, 5, 8, S133–S134
Synthesis of results	21	Present results of each meta-analysis done, including confidence/credible intervals. *In larger networks, authors may focus on comparisons versus a particular comparator (e.g. placebo or standard care), with full findings presented in an appendix. League tables and forest plots may be considered to summarize pairwise comparisons.* If additional summary measures were explored (such as treatment rankings), these should also be presented.	10–13, Tables 2–3, Figures 1 -7, S133–S134
**Exploration for inconsistency**	**S5**	Describe results from investigations of inconsistency. This may include such information as measures of model fit to compare consistency and inconsistency models, *P* values from statistical tests, or summary of inconsistency estimates from different parts of the treatment network.	10–13, Tables 5, Figures 3, 6, S53
Risk of bias across studies	22	Present results of any assessment of risk of bias across studies for the evidence base being studied.	14, S127–S128
Results of additional analyses	23	Give results of additional analyses, if done (e.g., sensitivity or subgroup analyses, meta-regression analyses*, alternative network geometries studied, alternative choice of prior distributions for Bayesian analyses,* and so forth).	12–13, S54–S126

**DISCUSSION**			
Summary of evidence	24	Summarize the main findings, including the strength of evidence for each main outcome; consider their relevance to key groups (e.g., healthcare providers, users, and policy-makers).	14–18
Limitations	25	Discuss limitations at study and outcome level (e.g., risk of bias), and at review level (e.g., incomplete retrieval of identified research, reporting bias). *Comment on the validity of the assumptions, such as transitivity and consistency. Comment on any concerns regarding network geometry (e.g., avoidance of certain comparisons).*	19
Conclusions	26	Provide a general interpretation of the results in the context of other evidence, and implications for future research.	19–20
**FUNDING**			
Funding	27	Describe sources of funding for the systematic review and other support (e.g., supply of data); role of funders for the systematic review. This should also include information regarding whether funding has been received from manufacturers of treatments in the network and/or whether some of the authors are content experts with professional conflicts of interest that could affect use of treatments in the network.	1

PICOS = Population, Intervention, Comparators, Outcomes, Study design.

* Text in italics indicates wording specific to reporting of network meta-analyses that has been added to guidance from the PRISMA statement.

† Authors may wish to plan for use of appendices to present all relevant information in full detail for items in this section.

Online Table 2. PRISMA-Pairwise.

Section / Topic	Item #	Checklist Item	Page #
**TITLE**			
Title	1	Identify the report as a systematic review.	1
**ABSTRACT**			
Abstract	2	See the PRISMA 2020 for Abstracts checklist.	2-3
**INTRODUCTION**			
Rationale	3	Describe the rationale for the review in the context of existing knowledge.	5-6
Objectives	4	Provide an explicit statement of the objective(s) or question(s) the review addresses.	6
**METHODS**			
Eligibility criteria	5	Specify the inclusion and exclusion criteria for the review and how studies were grouped for the syntheses.	7
Information sources	6	Specify all databases, registers, websites, organisations, reference lists and other sources searched or consulted to identify studies. Specify the date when each source was last searched or consulted.	7, S16–S17
Search strategy	7	Present the full search strategies for all databases, registers and websites, including any filters and limits used.	7, S27–S29
Selection process	8	Specify the methods used to decide whether a study met the inclusion criteria of the review, including how many reviewers screened each record and each report retrieved, whether they worked independently, and if applicable, details of automation tools used in the process.	S16–S17
Data collection process	9	Specify the methods used to collect data from reports, including how many reviewers collected data from each report, whether they worked independently, any processes for obtaining or confirming data from study investigators, and if applicable, details of automation tools used in the process.	7, S16–S17
Data items	10a	List and define all outcomes for which data were sought. Specify whether all results that were compatible with each outcome domain in each study were sought (e.g., for all measures, time points, analyses), and if not, the methods used to decide which results to collect.	S16–S17
	10b	List and define all other variables for which data were sought (e.g., participant and intervention characteristics, funding sources). Describe any assumptions made about any missing or unclear information.	S17–S18
Study risk of bias assessment	11	Specify the methods used to assess risk of bias in the included studies, including details of the tool(s) used, how many reviewers assessed each study and whether they worked independently, and if applicable, details of automation tools used in the process.	S17
Effect measures	12	Specify for each outcome the effect measure(s) (e.g., risk ratio, mean difference) used in the synthesis or presentation of results.	7, S17–S18
Synthesis methods	13a	Describe the processes used to decide which studies were eligible for each synthesis (e.g., tabulating the study intervention characteristics and comparing against the planned groups for each synthesis (item #5)).	S17–S18
	13b	Describe any methods required to prepare the data for presentation or synthesis, such as handling of missing summary statistics, or data conversions.	7, S17–S18
	13c	Describe any methods used to tabulate or visually display results of individual studies and syntheses.	8–9, S17–S18

Section / Topic	Item #	Checklist Item	Page #
	13d	Describe any methods used to synthesize results and provide a rationale for the choice(s). If meta-analysis was performed, describe the model(s), method(s) to identify the presence and extent of statistical heterogeneity, and software package(s) used.	8–9, S17–S18
	13e	Describe any methods used to explore possible causes of heterogeneity among study results (e.g., subgroup analysis, meta-regression).	S18–S19
	13f	Describe any sensitivity analyses conducted to assess robustness of the synthesized results.	S18–S19
Reporting bias assessment	14	Describe any methods used to assess risk of bias due to missing results in a synthesis (arising from reporting biases).	8-9
Certainty assessment	15	Describe any methods used to assess certainty (or confidence) in the body of evidence for an outcome.	S18–S19
**RESULTS**			
Study selection	16a	Describe the results of the search and selection process, from the number of records identified in the search to the number of studies included in the review, ideally using a flow diagram.	9, S10–S15, S129
	16b	Cite studies that might appear to meet the inclusion criteria, but which were excluded, and explain why they were excluded.	-
Study characteristics	17	Cite each included study and present its characteristics.	Table 1, S10–S1, S30–S52
Risk of bias in studies	18	Present assessments of risk of bias for each included study.	14, S127–S128
Results of individual studies	19	For all outcomes, present, for each study: (a) summary statistics for each group (where appropriate) and (b) an effect estimate and its precision (e.g. confidence/credible interval), ideally using structured tables or plots.	13–14, Figure 8
Results of syntheses	20a	For each synthesis, briefly summarise the characteristics and risk of bias among contributing studies.	S131–S132
	20b	Present results of all statistical syntheses conducted. If meta-analysis was done, present for each the summary estimate and its precision (e.g. confidence/credible interval) and measures of statistical heterogeneity. If comparing groups, describe the direction of the effect.	13–14, Table 6, Figure 8
	20c	Present results of all investigations of possible causes of heterogeneity among study results.	13–14, Table 6, Figure 8, S118–S126
	20d	Present results of all sensitivity analyses conducted to assess the robustness of the synthesized results.	S118–S126
Reporting biases	21	Present assessments of risk of bias due to missing results (arising from reporting biases) for each synthesis assessed.	14, S136
Certainty of evidence	22	Present assessments of certainty (or confidence) in the body of evidence for each outcome assessed.	13–14, Table 6, Figure 8, S118–S126,

Section / Topic	Item #	Checklist Item	Page #
			S136
**DISCUSSION**			
Discussion	23a	Provide a general interpretation of the results in the context of other evidence.	14–18
	23b	Discuss any limitations of the evidence included in the review.	19
	23c	Discuss any limitations of the review processes used.	19
	23d	Discuss implications of the results for practice, policy, and future research.	19–20
**OTHER INFORMATION**			
Registration and protocol	24a	Provide registration information for the review, including register name and registration number, or state that the review was not registered.	6, S5–S9
	24b	Indicate where the review protocol can be accessed, or state that a protocol was not prepared.	6, S5–S9
	24c	Describe and explain any amendments to information provided at registration or in the protocol.	6, S5–S9
Support	25	Describe sources of financial or non-financial support for the review, and the role of the funders or sponsors in the review.	1
Competing interests	26	Declare any competing interests of review authors.	1
Availability of data, code and other materials	27	Report which of the following are publicly available and where they can be found: template data collection forms; data extracted from included studies; data used for all analyses; analytic code; any other materials used in the review.	49

Online Table 3. Search Strategy.

Database	Search String	Results
**MedLine/Pubmed**	((“Tomography, Optical Coherence”[MeSH] OR "optical coherence tomography guided"[Title/Abstract] OR "OCT-Guided"[Title/Abstract] OR "OCT guidance"[Title/Abstract] OR "optical coherence tomography guided"[Title/Abstract] OR ("OCT"[All Fields] AND "guided"[Title/Abstract]) OR "OCT-guided PCI"[Title/Abstract] OR "Optical Frequency Domain Imaging"[Title/Abstract] OR "OFDI"[Title/Abstract] OR "OFDI-guided PCI"[Title/Abstract] OR "Optical coherence tomography"[Title/Abstract]) AND ("IVUS"[Title/Abstract] OR "intravascular ultrasound"[Title/Abstract] OR "ivus guid*"[Title/Abstract] OR "intravascular ultrasound guid*"[Title/Abstract] OR "Intravascular Ultrasound-Guided Drug-Eluting Stent"[Title/Abstract] OR "intravascular ultrasound-guided"[Title/Abstract] OR "intravascular ultrasonography"[Title/Abstract] OR "IVUS-guided PCI"[Title/Abstract] OR "intravascular imaging guid*"[Title/Abstract])) OR (("OCT"[Title/Abstract] OR "optical coherence tomography guided"[Title/Abstract] OR "OCT-Guided"[Title/Abstract] OR "OCT guidance"[Title/Abstract] OR "optical coherence tomography guided"[Title/Abstract] OR ("OCT"[All Fields] AND "guided"[Title/Abstract]) OR "OCT-guided PCI"[Title/Abstract] OR "Optical Frequency Domain Imaging"[Title/Abstract] OR "OFDI"[Title/Abstract] OR "OFDI-guided PCI"[Title/Abstract] OR "Optical coherence tomography"[Title/Abstract]) AND ("angiography-guided"[Title/Abstract] OR "angio-guided"[Title/Abstract] OR "angiography guid*"[Title/Abstract] OR "angiography guid*"[Title/Abstract] OR "invasive coronary treatment"[Title/Abstract] OR “Coronary angiography”[MeSH] OR "angiographically guided"[Title/Abstract] OR "CAG-guided"[Title/Abstract] OR "coronary arteriography"[Title/Abstract] OR "angiographic guidance"[Title/Abstract])) OR (("IVUS"[Title/Abstract] OR "intravascular ultrasound"[Title/Abstract] OR "ivus guid*"[Title/Abstract] OR "intravascular ultrasound guid*"[Title/Abstract] OR "Intravascular Ultrasound-Guided Drug-Eluting Stent"[Title/Abstract] OR "intravascular ultrasound-guided"[Title/Abstract] OR "intravascular ultrasonography"[Title/Abstract] OR "IVUS-guided PCI"[Title/Abstract] OR "intravascular imaging guid*"[Title/Abstract]) AND ("angiography-guided"[Title/Abstract] OR "angio-guided"[Title/Abstract] OR "angiography guid*"[Title/Abstract] OR "angiography guid*"[Title/Abstract] OR "invasive coronary treatment"[Title/Abstract] OR "coronary angiography"[Title/Abstract] OR "angiographically	**4,250**

	guided"[Title/Abstract] OR "CAG-guided"[Title/Abstract] OR "coronary arteriography"[Title/Abstract] OR "angiographic guidance"[Title/Abstract]))	
**Scopus**	((INDEXTERMS("Tomography, Optical Coherence") OR TITLE-ABS("optical coherence tomography guided") OR TITLE-ABS(OCT-Guided) OR TITLE-ABS("OCT guidance") OR TITLE-ABS("optical coherence tomography guided") OR (ALL(OCT) AND TITLE-ABS(guided)) OR TITLE-ABS("OCT-guided PCI") OR TITLE-ABS("Optical Frequency Domain Imaging") OR TITLE-ABS(OFDI) OR TITLE-ABS("OFDI-guided PCI") OR TITLE-ABS("Optical coherence tomography")) AND (TITLE-ABS(IVUS) OR TITLE-ABS("intravascular ultrasound") OR TITLE-ABS("ivus guid*") OR TITLE-ABS("intravascular ultrasound guid*") OR TITLE-ABS("Intravascular Ultrasound-Guided Drug-Eluting Stent") OR TITLE-ABS("intravascular ultrasound-guided") OR TITLE-ABS("intravascular ultrasonography") OR TITLE-ABS("IVUS-guided PCI") OR TITLE-ABS("intravascular imaging guid*"))) OR ((TITLE-ABS(OCT) OR TITLE-ABS("optical coherence tomography guided") OR TITLE-ABS(OCT-Guided) OR TITLE-ABS("OCT guidance") OR TITLE-ABS("optical coherence tomography guided") OR (ALL(OCT) AND TITLE-ABS(guided)) OR TITLE-ABS("OCT-guided PCI") OR TITLE-ABS("Optical Frequency Domain Imaging") OR TITLE-ABS(OFDI) OR TITLE-ABS("OFDI-guided PCI") OR TITLE-ABS("Optical coherence tomography")) AND (TITLE-ABS(angiography-guided) OR TITLE-ABS(angio-guided) OR TITLE-ABS("angiography guid*") OR TITLE-ABS("angiography guid*") OR TITLE-ABS("invasive coronary treatment") OR INDEXTERMS("Coronary angiography") OR TITLE-ABS("angiographically guided") OR TITLE-ABS(CAG-guided) OR TITLE-ABS("coronary arteriography") OR TITLE-ABS("angiographic guidance"))) OR ((TITLE-ABS(IVUS) OR TITLE-ABS("intravascular ultrasound") OR TITLE-ABS("ivus guid*") OR TITLE-ABS("intravascular ultrasound guid*") OR TITLE-ABS("Intravascular Ultrasound-Guided Drug-Eluting Stent") OR TITLE-ABS("intravascular ultrasound-guided") OR TITLE-ABS("intravascular ultrasonography") OR TITLE-ABS("IVUS-guided PCI") OR TITLE-ABS("intravascular imaging guid*")) AND (TITLE-ABS(angiography-guided) OR TITLE-ABS(angio-guided) OR TITLE-ABS("angiography guid*") OR TITLE-ABS("angiography guid*") OR TITLE-ABS("invasive coronary treatment") OR TITLE-ABS("coronary angiography") OR TITLE-ABS("angiographically guided") OR TITLE-ABS(CAG-guided) OR TITLE-ABS("coronary arteriography") OR TITLE-ABS("angiographic guidance")))	**4,589**
**Web of Science**	((ALL=("optical coherence tomography" OR "optical coherence tomography guided" OR "OCT-guided PCI" OR "Optical Frequency Domain Imaging" OR OFDI OR "OFDI-guided PCI" OR OCT)) AND ALL=(IVUS OR "intravascular ultrasound" OR "intravascular ultrasonography" OR "IVUS-	**6,103**

	guided PCI")) OR ((ALL=(angiography-guided OR angio-guided OR "invasive coronary treatment" OR "coronary angiography" OR CAG-guided OR "coronary arteriography")) AND ALL=("optical coherence tomography" OR "optical coherence tomography guided" OR "OCT-guided PCI" OR "Optical Frequency Domain Imaging" OR OFDI OR "OFDI-guided PCI" OR OCT)) OR ((ALL=(angiography-guided OR angio-guided OR "invasive coronary treatment" OR "coronary angiography" OR CAG-guided OR "coronary arteriography")) AND ALL=(IVUS OR "intravascular ultrasound" OR "intravascular ultrasonography" OR "IVUS-guided PCI"))<br>Refined by Document Types: Article and Meeting Abstract	
**Cochrane Library**	(("optical coherence tomography" OR "optical coherence tomography guided" OR "OCT-guided PCI" OR "Optical Frequency Domain Imaging" OR OFDI OR "OFDI-guided PCI" OR OCT) AND (IVUS OR "intravascular ultrasound" OR "intravascular ultrasonography" OR "IVUS-guided PCI")) OR (("optical coherence tomography" OR "optical coherence tomography guided" OR "OCT-guided PCI" OR "Optical Frequency Domain Imaging" OR OFDI OR "OFDI-guided PCI" OR OCT) AND (angiography-guided OR angio-guided OR "invasive coronary treatment" OR "coronary angiography" OR CAG-guided OR "coronary arteriography")) OR ((IVUS OR "intravascular ultrasound" OR "intravascular ultrasonography" OR "IVUS-guided PCI")<br>AND (angiography-guided OR angio-guided OR "invasive coronary treatment" OR "coronary angiography" OR CAG-guided OR "coronary arteriography")); Refined by Document types: Trial	**990**

The last search was run on August 28th, 2023.

Online Table 4. Endpoints Across Trials.

Trial	Target Lesion Revascularization	Myocardial Infarction	Death	Cardiac Death	Target Vessel Myocardial Infarction	Ischemia- Driven Target Lesion Revascularization	Target Vessel Revascularization	Definite or Probable Stent Thrombosis
**AIR-CTO**	X	X	X	X	xx	x	X	X
**AVIO**	X	X	xx	X	xx	x	X	X
**CTO-IVUS**	X	X	X	X	x‡	x	X	X
**DOCTORS**	xx	X	X	xx	x‡	xx	X	X
**EROSION III**	X	X	xx	X	X	X	xx	NA
**GUIDE-DES**	X	X	X	X	X	x§	X	X
**HOME DES IVUS**	X	X	X	x†	xx	x	xx	X
**ILUMIEN III**	X	X	X	X	X	X	xx	x
**ILUMIEN IV**	X	X	X	X	X	X	X	X
**iSIGHT**	X	x	X	X	X	x§	xx	X
**IVUS-XPL**	X	x*	xx	X	X	X	xx	X
**Kala et al.**	X	X	X	x†	xx	X	xx	X
**Kim et al.**	X	X	X	X	xx	x	xx	X
**Li et al.**	X	X	X	—	xx	x§	xx	NA
**Liu et al.**	X	X	xx	X	xx	x	X	X
**MISTIC-1**	X	X	X	X	X	x	X	X
**OCTACS**	X	X	X	X	X	X	X	X
**OCTIVUS**	X	X	X	X	X	x§	X	X
**OCTOBER**	X	X	X	X	X	X	X	X
**OPINION**	X	X	X	X	X	X	X	x
**RENOVATE-COMPLEX-PCI**	X	X	X	X	X	x	X	x
**RESET**	xx	X	X	X	xx	xx	X	x
**Tan et al.**	X	X	xx	x	xx	x	—	X
**ULTIMATE**	X	x	X	X	X	X	X	X
**Wang et al.**	xx	X	xx	X	X	xx	X	X

X=Consistent; x=Mildly inconsistent; xx=Moderately inconsistent; NA=Not Available.

In the primary analysis trials with inconsistent definitions were considered. In a focused sensitivity analysis (**Online Tables 19-20**) only trials with similar definitions were pooled.

* For the primary analysis, the IVUS-XPL estimates included periprocedural events differently from the original analysis of the trial.

† Although only death was reported, the number of events was deemed equivalent to cardiac death.
‡ Although only myocardial infarction was reported, the number of events was deemed closely equivalent to target vessel myocardial infarction.
§ Although only target lesion revascularization was reported, the number of events was deemed closely equivalent to ischemia-driven target lesion revascularization.

Online Table 5. Sensitivity Analysis by Definition.

Trial	Target Lesion Revascularization	Myocardial Infarction	Death	Cardiac Death	Target Vessel Myocardial Infarction	Ischemia- Driven Target Lesion Revascularization	Target Vessel Revascularization	Definite or Probable Stent Thrombosis
AIR-CTO	X	X	X	X	—	—	X	X
AVIO	X	X	—	X	—	—	X	X
CTO-IVUS	X	X	X	X	X	—	X	X
DOCTORS	—	X	X	—	X	—	X	X
EROSION III	X	X	—	X	X	X	—	NA
GUIDE-DES	X	X	X	X	X	X	X	X
HOME DES IVUS	X	X	X	X	—	—	—	X
ILUMIEN III	X	X	X	X	X	X	—	—
ILUMIEN IV	X	X	X	X	X	X	X	X
iSIGHT	X	—	X	X	X	X	—	X
IVUS-XPL	X	—	—	—	X	X	—	X
Kala et al.	X	X	X	X	—	X	—	X
Kim et al.	X	X	X	X	—	—	—	X
Li et al.	X	X	X	X	—	X	—	NA
Liu et al.	X	X	—	X	—	—	X	X
MISTIC-1	X	X	X	X	X	—	X	X
OCTACS	X	X	X	X	X	X	X	X
OCTIVUS	X	X	X	X	X	X	X	X
OCTOBER	X	X	X	X	X	X	X	X
OPINION	X	X	X	X	X	X	X	—
RENOVATE-COMPLEX-PCI	X	X	X	X	X	X	X	—
RESET	—	X	X	X	—	—	X	—
Tan et al.	X	X	—	X	—	—	—	X
ULTIMATE	X	—	X	X	X	X	X	X
Wang et al.	—	X	—	X	X	—	X	X

X=Included in the sensitivity analysis; —=Excluded from the sensitivity analysis; NA=Not Available.

Online Table 6. Major Adverse Cardiac Events Definitions Across Included Studies.

Trial	Major Adverse Cardiac Events Definition	Sensitivity
**AIR-CTO**	Not Available	—
**AVIO**	Cardiac Death, Myocardial Infarction, or Target Vessel Revascularization	—
**CTO-IVUS**	Cardiac Death, Myocardial Infarction, or Target Vessel Revascularization	—
**DOCTORS**	Death, Myocardial Infarction, or Target Vessel Revascularization	—
**EROSION III**	Cardiac Death, Recurrent Myocardial Infarction, Target Lesion Revascularization, Stroke, Heart Failure, Malignant Arrhythmia, or Rehospitalization	—
**GUIDE-DES**	Cardiac Death, Target Vessel Myocardial Infarction, or Ischemia-Driven Target Lesion Revascularization	X
**HOME DES IVUS**	Death, Myocardial Infarction, or Target Lesion Revascularization	X
**ILUMIEN III**	Cardiac Death, Target Vessel Myocardial Infarction, or Ischemia-Driven Target Lesion Revascularization	X
**ILUMIEN IV**	Cardiac Death, Target Vessel Myocardial Infarction, or Ischemia-Driven Target Lesion Revascularization	X
**iSIGHT**	Cardiac Death, Nonfatal Myocardial Infarction, or Target Lesion Revascularization	X
**IVUS-XPL**	Cardiac Death, Nonfatal Myocardial Infarction, or Target Lesion Revascularization	X
**Kala et al.**	Death, Myocardial Infarction, or Target Lesion Revascularization	X
**Kim et al.**	Cardiac Death, Nonfatal Myocardial Infarction, or Target Lesion Revascularization	X
**Li et al.**	Cardiac Death, Nonfatal Myocardial Infarction, or Target Lesion Revascularization	X
**Liu et al.**	Cardiac Death, Myocardial Infarction, or Target Vessel Revascularization	X
**MISTIC-1**	Cardiac Death, Target Vessel Myocardial Infarction, or Clinically-Driven Target Lesion Revascularization	X
**OCTACS**	Cardiac Death, Target Vessel Myocardial Infarction, or Ischemia-Driven Target Lesion Revascularization	X
**OCTIVUS**	Cardiac Death, Target Vessel Myocardial Infarction, or Ischemia-Driven Target Lesion Revascularization	X
**OCTOBER**	Cardiac Death, Myocardial Infarction, or Ischemia-Driven Target Lesion Revascularization	X
**OPINION**	Cardiac Death, Target Vessel Myocardial Infarction, or Ischemia-Driven Target Lesion Revascularization	X
**RESET**	Cardiovascular Death, Myocardial Infarction, Stent Thrombosis, or Target Vessel Revascularization	—
**Tan et al.**	Cardiovascular Death, Myocardial Infarction, or Target Lesion Revascularization	X
**ULTIMATE**	Cardiac Death, Target Vessel Myocardial Infarction, or Clinically-Driven Target Lesion Revascularization	X
**Wang et al.**	Cardiac Death, Myocardial Infarction, or Target Vessel Revascularization	—

Online Table 7. Main Clinical Characteristics Across Trials.

Trial	Clinical Presentation	Age	Females	Diabetes	Multivessel Disease	Lesions per Patient	Bifurcation	Left Main
**AIR-CTO**	• CCS: 73.5<br>• UA: 9.2<br>• AMI > 24 h: 17.3	66.5	15.7	28.3	83.9	1	14.8	-
**AVIO**	• UA: 27.9	63.8	20.4	25.4	-	1.3	19.1	0
**CTO-IVUS**	• CCS: 100.0	61.2	19.4	34.3	67.2	1	24.9 °	0
**DOCTORS**	• UA: 19.0<br>• ACS: 81.0	60.5	22.5	18.8	30.8	1	-	0
**EROSION III**	• ACS: 100.0	55.5	20.4	21.2	-	1	-	0
**GUIDE-DES**	• CCS: 70.9<br>• NSTE-ACS: 22.0<br>• STEMI: 7.1	64.3	22.7	32.3	50	1.2	67.2	12.8
**HOME DES IVUS**	• CCS: 39.0<br>• UA/NSTE-ACS: 41.0<br>• STEMI: 25.0	59.8	28.0	43.5	57	1.2	-	3.5
**ILUMIEN III**	• CCS: 63.5<br>• UA: 18.9<br>• NSTE-ACS: 14<br>• STEMI: 3.6	66.3	28.2	33.1	-	-	0	0
**ILUMIEN IV**	• CCS: 42.5<br>• UA: 27.6<br>• NSTE-ACS: 24.2<br>• Recent STEMI: 5.7	65.6	22.6	42.0	-	1	3.3	0
**iSIGHT**	• CCS: 40.6<br>• UA/NSTE-ACS: 38.7<br>• Recent MI: 20.7	59.3	30.0	39.3	-	1	0	0
**IVUS-XPL**	• CCS: 51.0<br>• UA: 33.5<br>• AMI: 15.5	64.0	31.0	36.5	68.6	1.3	0	0

**Kala et al.**	• STEMI: 100.0	58.0	15.1	21.3	10.6	1	-	0
**Kim et al.**	• CCS: 61.4<br>• ACS: 38.6	60.2	24.8	31.7	-	1	0	0
**Li et al.**	• CCS: 100.0	57.8	46.9	100.0	53.9	1.2	36.8	17.0
**Liu et al.**	• CCS: 13.4<br>• UA: 75.3<br>• Recent MI: 11.3	65.1	36.3	32.1	83.6	1.6	60.1	100.0
**MISTIC-1**	• CCS: 100.0	71.5	22.0	46.8	40.3	1.2	-	0
**OCTACS**	• NSTE-ACS: 100.0	62.2	30	13	38.0	1	0	0
**OCTIVUS**	• CCS: 76.6<br>• UA: 13.5<br>• NSTE-ACS: 9.9	64.7	21.6	33.3	61.6	1.3	52.6	13.5
**OCTOBER**	• CCS: 54.2<br>• UA: 9.2<br>• NSTE-ACS: 13.1<br>• Staged procedure after AMI: 23.5	66.3	21.1	16.7	18.9	1	100.0	16.5
**OPINION**	• CCS: 87.5<br>• UA: 12.5	68.5	22.0	40.9	-	1	38.4	0
**RENOVATE-COMPLEX PCI**	• CCS: 49.2<br>• UA: 32.6<br>• NSTE-ACS: 15.7<br>• STEMI: 2.4	65.6	20.7	37.6	67.9	1.4	21.9	11.7
**RESET**	• CCS: 52.3<br>• UA: 38.3<br>• AMI: 9.4	63.6	39.8	30.8	39.0	1.4	0	0
**Tan et al.**	• Stable Angina: 31.8<br>• UA: 68.2	76.1	34.1	31.7	86.2	1	53.7	100
**ULTIMATE**	• CCS: 21.5<br>• UA: 65.8	65.5	26.5	30.6	54.9	1.4	25.0	9.2

	• AMI: 12.5							
**Wang et al.**	• STEMI: 100	55.0	36.2	16.3	-	1	7.5	0

AMI=Acute Myocardial Infarction; CCS=Chronic Coronary Syndrome; NSTE-ACS= Non-ST-Segment Elevation Myocardial Infarction; STEMI= ST-Segment Elevation Myocardial Infarction; UA=Unstable Angina.

Online Table 8. Main Procedural Characteristics Across Trials.

Trial Name, Year	Stent per Lesion[a] / Patient[b] (%)	Stent Length per Lesion[a]/ Patient[b] (mm)	Mean[c] / Maximum[d] Stent Diameter (mm)	DES Strut Thickness[e] (micron)	DES Drug Eluted[f]	Post-Dilation (%)	Mean[c] / Maximum[d] Balloon Diameter (mm)	Maximum Post-Dilation Pressure (atm)	Procedure Duration (min)	Contrast Volume (mL)
**AIR-CTO**	1.5[b]	53.5[b]	2.9[c]	-	Sirolimus	-	-	-	73.5	293.0
**AVIO**	-	23.5[b]	2.9[c]	-	-	78.3	3.2[c]	19.9	-	-
**CTO-IVUS**	1.65[a]	42.3[a]	2.9[d]	81<br>120	Zotarolimus<br>Biolimus	-	-	14.2	91.5	297.0
**DOCTORS**	1.2 [b]	21.1 [b]	-	-	-	27.2	-	-	46	155.0
**EROSION III**	1.1[a] / 0.5[b]	28.5[a] / 14.6[b]	3.5[d]	-	-	82.2	3.5[d]	-	-	-
**GUIDE-DES**	1.6 [b]	35.1[b]	-	60-80	Sirolimus	-	3.5[d]	20.0	-	-
**HOME DES IVUS**	1.1[a] /1.3[b]	22.9[b]	-	132<br>140	Sirolimus<br>Paclitaxel	16.9	3.2[c]	15.8	43.5	122.2
**ILUMIEN III**	1.0 [a]	22.5[b]	3.0[d]	-	Everolimus<br>Zotarolimus<br>Sirolimus	-	3.3[d]	18.6	67.2	199.0
**ILUMIEN IV**	-	42.3[b]	3.1[d]	81	Everolimus	89.3	-	19.0	59.0	214.9
**iSIGHT**	1.1[a]	29.0[b]	3.3[c]	81	Zotarolimus<br>Everolimus	100.0	3.5[d]	21.3	55.0	82.8
**IVUS-XPL**	1.3 [a]	39.2[b]	-	81	Everolimus	66.5	3.0[c] /3.1[d]	16.0	-	-
**Kala et al.**	1.3[b]	-	-	81<br>120	Biolimus<br>Everolimus	-	-	17.0	-	200.3
**Kim et al.**	-	17.8[a] /18.5[b]	3.2[c]	81	Zotarolimus	33.2	3.3[d]	16.2	-	-

**Li et al.**	1.5[a]/1.2[b]	19.1[a]	2.7[c]	81	Everolimus	64.5	-	-	-	-
**Liu et al.**	2.3[b]	33.0[b]	3.4[c]	-	-	-	3.5[c]	14.6	-	-
**MISTIC-1**	1.0[a] /18.0[b]	20.8[b]	3.0[c]	120	Biolimus	65.8	3.4[d]	18.0	72.0	132.9
**OCTACS**	1.0[a]	21.3[a]	3.0[c]	120	Biolimus	-	3.3[d]	15.9	37.5	130.0
**OCTOBER**	1.0[b]	35.5[b]	-	81[g]	Everolimus	-	4.1[d]	-	-	250.0
**OCTIVUS**	1.3[a] /1.6[b]	47.5[b]	3.3[d]	81<br>81<br>60	Everolimus<br>Zotarolimus<br>Sirolimus	92.0	3.7[d]	22.1	-	218.5
**OPINION**	25.3[b]	-	3.0[c]	120<br>-<br>-	Biolimus<br>Everolimus<br>Zotarolimus	75.9	3.2[d]	16	-	151.0
**RENOVATE-COMPLEX-PCI**	1.3[a]	37.6[a]	3.1[c]	74<br>81	Everolimus<br>Everolimus	-	3.5[c]	18.9	64.4	206.6
**RESET**	-	30.0[a] /32.3[b]	-	91<br>81	Zotarolimus<br>Everolimus	49.5	3.1[d]	13.5	-	-
**Tan et al.**	-	19.8[b]	-	86<br>88	Sirolimus<br>Sirolimus	16.0	-	-	-	-
**ULTIMATE**	1.8[a] /2.5[b]	47.9[a] /66.5[b]	3.1[c]	-	Zotarolimus<br>Everolimus<br>Sirolimus	-	3.6[d]	19.4	52.2	169.2
**Wang et al.**	1.1[b]	-	-	-	-	-	-	-	-	-

DES=Drug-Eluting Stent.

[a] Refers to characteristics per lesion; [b] Refers to characteristics per patient; [c] Refers to mean value across the study population; [d] Refers to maximum mean value across the study population; [e] The stent choice was based on the operator's choice and both drug-eluting and bare metal stents were allowed, though these figures were not quantified in the study results; however, considering the study period, the predominant use of drug-eluting stents was assuumed; [f] Drug eluting stent implanted in less than 5% of cases were not reported in this table; [g] Other unspecified drug eluting stents were implanted in less of 20% of cases.

Online Table 9. Inclusion and Exclusion Criteria Across Trials.

Study	Inclusion Criteria	Exclusion Criteria
**AIR-CTO**	General Inclusion Criteria:<br>• At least 18 years of age at the time of consent.<br>• Clinical symptoms suggestive of ischemic heart disease or evidence of ischemia attributed to the chronic total occlusion target vessel and scheduled for clinically indicated PCI<br>• Eligibility and consensus to undergo PCI<br>• Acceptable candidate for PCI, stenting, and emergency coronary artery bypass grafting<br>• Willing and able to sign a study consent form<br>• Female participants of childbearing potential with a negative pregnancy test per standard of care for PCI and be practicing contraception<br><br>Angiographic Criteria:<br>• A minimum of one de novo lesion with at least one target segment in a native coronary vessel meeting the definition of chronic total occlusion and estimated to be in duration of ≥3 months by clinical history and/or comparison with antecedent angiogram or electrocardiogram	General Exclusion Criteria:<br>• History of allergy to iodinated contrast that cannot be effectively managed medically<br>• Evidence of acute myocardial infarction within 72 hours prior to the intended treatment<br>• Previous coronary interventional procedure of any kind within 30 days prior to the procedure<br>• Any contraindication to cardiac catheterization or to any of the standard concomitant therapies used during routine cardiac catheterization and PCI<br>• Target lesion requires treatment with another device, after successful crossing with a study device, other than PCI devices prior to stent placement<br>• Atherectomy procedure is planned for the target lesion<br>• Known history of clinically significant abnormal laboratory findings ≤14 days prior to enrollment, including neutropenia, thrombocytopenia, hepatic enzymes, alkaline phosphatase, or bilirubin >1.5x upper limit of normal, and serum creatinine >2.0 mg/dL<br>• Evidence of current clinical instability including sustained systolic blood pressure <100 mmHg or cardiogenic shock; acute pulmonary edema or severe chronic heart failure, suspected acute myocarditis, pericarditis, endocarditis, or cardiac tamponade; suspected dissecting aortic aneurysm; hemodynamically significant valvular heart disease, hypertrophic cardiomyopathy, restrictive cardiomyopathy, or congenital heart disease<br>• History of stroke or transient ischemic attac within 6 months prior to procedure; active peptic ulcer or upper gastrointestinal bleeding within 6 months prior to procedure; history of bleeding diathesis or coagulopathy or refusal of blood transfusions; other pathology such as cancer, known mental illness, etc., which might, in the opinion of the Investigator, put the patient at risk or confound the results of the study<br>• Unable or unwilling to comply with the protocol

		• Currently participating in an investigational drug or device study that has not completed the primary endpoint or that clinically interferes with the current study endpoints.<br><br><u>Angiographic exclusion criteria</u><br>• Occlusion involves segment within previous stent<br>• Extensive lesion-related thrombus (TIMI thrombus grade 3 or 4)<br>• Previous stenting in the target vessel unless the following conditions are met: (1) it has been at least 9 months since the previous stenting; (2) target lesion is ≥15 mm away from the previously placed stent; (3) previously stented segment has ≤40% diameter stenosis<br>• Target vessel has other lesions proximal to the total occlusion identified with >75% diameter stenosis based on visual estimate
**AVIO**	• Patients of at least 18 years of age<br>• Complex lesions (lesions length >28 mm, total occlusion of duration more than 3-months, bifurcation disease, reference vessel diameter ≤2.50 mm, or ≥4 stents	• Contraindication to dual antiplatelet therapy<br>• Left ventricular ejection fraction <30%<br>• Renal failure (creatinine >2 mg/dL)<br>• Significant co-morbidity precluding clinical follow-up<br>• Acute myocardial infarction in the 48 hours prior to the procedure<br>• In-stent restenosis<br>• Prior brachytherapy<br>• Thrombocytopenia <100,000<br>• Unprotected left main stenosis<br>• Venous or arterial grafts<br>• Recipient of heart transplant<br>• A positive pregnancy test in women with childbearing potential<br>• Acute infections<br>• Major surgery planned which will lead to discontinuation of antiplatelet therapy<br>• Patients with prior bare metal stent or drug-eluting stent implanted in the target vessel less than one year prior to the enrollment, including one year from any intercurrent restenotic or thrombotic event
**CTO-IVUS**	• Patient ≥20 or ≤80 years old.<br>• Total obstruction of coronary blood flow (Thrombolysis in Myocardial Infarction grade 0) with estimated occlusion duration ≥3 months	• Hypersensitivity reaction or side effects to aspirin, clopidogrel, biolimus A9, and zotarolimus<br>• Unprotected left main disease<br>• Cardiogenic shock or left ejection ejection fraction ≤30%

	• Reference vessel diameter of 2.5 to 4.0 mm by operator assessment<br>• Total length of total occluded lesion and main lesion less than 80 mm and lesions can be treated less than 4 stents<br>• Guide wire could be passed through occluded lesion without complications<br>• Patients who could keep dual antiplatelet therapy more than 6 months after procedure	• Previous stent restenotic lesion<br>• Treated within 2 weeks at the same lesion<br>• Creatinine level ≥2.0 mg/dL or end-stage renal disease<br>• Severe tortuous and calcified lesion<br>• Life expectancy <1 year<br>• Severe hepatic dysfunction (>3 times normal reference values)<br>• Pregnant women or women with potential childbearing
**DOCTORS**	• Patients aged 18 to 80 years inclusive, admitted for acute coronary syndrome<br>• Patients presenting with an indication for coronary angioplasty with stent implantation of the target lesion considered to be responsible for the acute coronary syndrome<br>• Patients provided written informed consent	• Left main disease<br>• Presence of coronary artery bypass grafting<br>• Cardiogenic shock or severe hemodynamic instability<br>• Severely calcified or tortuous arteries.<br>• Persistent ST-segment elevation<br>• One or more other lesions considered angiographically significant and located on the target vessel<br>• Severe renal insufficiency (creatinine clearance ≤30 mL/min)<br>• Bacteraemia or septicaemia<br>• Severe coagulation disorders<br>• Patients who refuse to sign the informed consent form
**EROSION III**	• Patients aged 18 to 80 years old<br>• Patients with ST-segment elevation myocardial infarction <12h<br>• Target lesion located in a native coronary artery<br>• The residual diameter stenosis is ≤70% on angiogram and Thrombolysis in Myocardial Infarction flow grade is 3 after thrombus aspiration or not<br>• Written informed consent	• Patients who are breastfeeding or pregnant or planning to pregnant during the study period.<br>• Patients with a history of heart failure.<br>• Hemodynamic instability.<br>• Left main disease<br>• Three-vessel disease<br>• Ostial lesion<br>• Tortuous lesion<br>• Angulated lesion<br>• Subjects with contraindication of contrast medium<br>• Contraindications to aspirin or clopidogrel<br>• Severe hepatic and renal insufficiency (alanine-aminotransferase or arginine-aminotransferase >3x upper limits of normal, creatinine >2.0 mg/dL or end-stage renal disease)<br>• Patients with bleeding tendency, bleeding or coagulation disorders<br>• Acute myocardial infarction caused by surgery, trauma, gastrointestinal bleeding, PCI, or its complications

		• Acute myocardial infarction in patients hospitalized for other clinical reasons<br>• Poor compliance and low likelihood of adherence to the protocol as judged by the investigators<br>• Life expectancy ≤24 months<br>• Patients with heart transplantation<br>• Patients with diagnosis of tumors<br>• Patients who are currently enrolled in other clinical trial which has not reached its primary endpoint<br>• Patients who are not suitable for the current study judged by the investigators
**GUIDE-DES**	• Men or women at least 19 years of age<br>• Typical chest pain or objective evidence of myocardial ischemia suitable for elective PCI<br>• Significant coronary artery lesions suitable for sirolimus-eluting Orsiro or Orsiro Mission stent implantation.<br>• The patient or guardian agrees to the study protocol and the schedule of clinical follow-up, and provides informed, written consent, as approved by the appropriate Institutional Review Board/Ethical Committee of the respective clinical site	• Coronary artery bypass graft lesions<br>• Impaired delivery of intravascular ultrasound is expected, such as extreme angulation (≥90°) proximal to or within the target lesion, excessive tortuosity (≥2 45° angles) proximal to or within the target lesion, and heavy calcification proximal to or within the target lesion<br>• Previous PCI within 6 months before the index procedure<br>• Previous bioresorbable vascular scaffold implantation<br>• Left ventricular ejection fraction <30%.<br>• Hypersensitivity or contraindication to device material and its degradants that cannot be adequately pre-medicated.<br>• Persistent thrombocytopenia (platelet count <100,000/µl)<br>• Any history of hemorrhagic stroke or intracranial hemorrhage, transient or ischemic stroke within the past 6 month<br>• Known intolerance to antiplatelet agents<br>• Any surgery requiring general anesthesia or discontinuation of aspirin and/or an adenosine diphosphate antagonist planned within 12 months after the procedure<br>• Diagnosis of cancer in the past 3 years or current treatment for the active cancer<br>• Any clinically significant abnormality identified at the screening visit, physical examination, laboratory tests, or electrocardiogram which, in the judgment of the Investigator, would preclude safe completion of the study.<br>• Hepatic disease or biliary tract obstruction, or significant hepatic enzyme elevation (>3 times upper limit of normal)

		• Life expectancy <1 year for any non-cardiac or cardiac causes<br>• Unwillingness or inability to comply with the procedures described in this protocol<br>• Pregnancy or breast feeding or childbearing potential
**HOME DES IVUS**	• Acute coronary syndrome<br>• Complex coronary lesions (lesion type B2 and C, proximal left anterior descending disease, left main disease, reference vessel diameter <2.50 mm, lesion length >20 mm, or in-stent restenosis)<br>• Insulin-dependent diabetes mellitus	Not reported.
**ILLUMIEN III**	General Inclusion Criteria:<br>• Estimated creatinine clearance <30 ml/min using Cockcroft-Gault equation<br>• Age ≥18 years<br>• Patient with an indication for PCI including angina (stable or unstable), silent ischemia, non-ST-segment elevation myocardial infarction or ST-segment elevation myocardial infarction (>24 hours from initial presentation and stable)<br>• Patients will undergo cardiac catheterization and possible or definite PCI with intent to stent using any non-investigational metallic drug-eluting stent<br>• Signed written informed consent<br><br>Angiographic Inclusion Criteria:<br>• Target lesion located in a native coronary artery with visually estimated reference vessel diameter of ≥2.25 mm to ≤3.50 mm.<br>• Lesion length <40 mm.	General Exclusion Criteria:<br>• Estimated creatinine clearance <30 ml/min using Cockcroft-Gault equation<br>• ST-segment elevation myocardial infarction within 24 hours of initial time of presentation to the first treating hospital<br>• PCI within 24 hours preceding the study procedure<br>• PCI of a lesion within the target vessel within 12 months prior to the study procedure<br>• Planned use of bare-metal stent<br>• Planned use of bioresorbable scaffold<br>• Cardiogenic shock<br>• Mobitz II second degree or complete heart block<br>• Malignant ventricular arrhythmias requiring treatment<br>• Pulmonary edema<br>• Intubation<br>• Known left ventricular ejection fraction <30%<br>• Severe valvular disease<br>• Cerebrovascular accident or transient ischemic attack within the past 6 months, or any permanent neurologic defect attributed to cerebrovascular accident<br>• One or more co-morbidities which reduces life expectancy to less than 12 months<br>• Known allergy to protocol-required concomitant medications or iodinated contrast<br>• Patient is participating in any other investigational drug or device clinical trial that has not reached its primary endpoint

		• Women who are pregnant or breastfeeding<br><br><u>Angiographic Exclusion Criteria:</u><br>• The presence of any non-study lesion in the target vessel with angiographic diameter stenosis >50%, or any additional target vessel stenosis which requires PCI either during or within 12 months after the study procedure<br>• Left main diameter stenosis ≥30% or left main PCI planned.<br>• Study target lesion in a bypass graft.<br>• Ostial right coronary artery target lesion<br>• Chronic total occlusion target lesion.<br>• Bifurcation lesion with a planned dual stent strategy<br>• In-stent restenosis study target lesion<br>• Any study lesion characteristic resulting in the expected inability to deliver the IVUS or OCT catheter to the lesion pre and post PCI
**ILUMIEN IV**	• At least 18 years of age<br>• Evidence of myocardial ischemia, unstable angina, or acute myocardial infarction suitable for elective PCI<br>• Patients undergoing planned Xience stent implantation during a clinically indicated PCI procedure meeting one or more of the following criteria: 1) High clinical-risk, defined as medication-treated diabetes mellitus, and/or (b) high angiographic-risk lesion(s), with at least one target lesion in each target vessel planned for randomization meeting at least one of the following criteria: (i) Target lesion is the culprit lesion responsible for either: non-ST-segment elevation myocardial infarction, or ST-segment elevation myocardial infarction >24 hours from the onset of ischemic symptoms; (ii) long or multiple lesions (defined as intended total stent length in any single target vessel ≥28 mm), (iii) bifurcation intended to be treated with 2 planned stents, and where the planned side branch stent is ≥2.5 mm in diameter by angiographic visual estimation; (iv) angiographic severe calcification; (v) chronic	• Patients with ST-segment elevation myocardial infarction ≤24 hours from the onset of ischemic symptoms<br>• Patients with creatinine clearance ≤30 ml/min/1.73 m^2 and not on dialysis<br>• Patients with hypotension, shock or need for mechanical support or intravenous vasopressors at the time of the index procedure<br>• Patients with chronic heart failure (Killip class ≥2 or New York Heart Association class ≥III)<br>• Patients with left ventricular ejection fraction ≤30% by the most recent imaging test within 3 months prior to procedure<br>• Patients with unstable ventricular arrhythmias<br>• Patients with inability to take dual antiplatelet therapy for at least 12 months in the those presenting with an acute coronary syndrome, or at least 6 months in those presenting with stable coronary artery disease, unless the patient is also taking chronic oral anticoagulation in which case a shorter duration of dual antiplatelet therapy may be prescribed per local standard of care<br>• Patients with planned major cardiac or non-cardiac surgery within 24 months after the index procedure.<br>• Patients who underwent prior PCI within the target vessel within 12 months.

	total occlusion; (vi) in-stent restenosis of diffuse or multi-focal pattern with angiographically-assessed diameter stenosis ≥70% or non-invasive or invasive evidence of ischemia and angiographically-assessed diameter stenosis ≥50%<br>• Target lesions including a visually estimated or quantitatively assessed percentage diameter stenosis of either ≥70%, or ≥ 50% plus one or more of the following: an abnormal functional test signifying ischemia in the distribution of the target lesion(s) or biomarker positive acute coronary syndrome with plaque disruption or thrombus<br>• Target lesion planned for treatment with only ≥2.50 mm and ≤3.50 mm stents and post- dilatation balloons based on pre-PCI angiographic visual estimation<br>• No more than 2 target lesions requiring PCI in any single vessel and no more than 2 target vessels, for a total of no more than 4 randomized target lesions per patient in a maximum of 2 target vessels, including their branches<br>• Target lesions intended to be treated by PCI in the target vessel are amenable to OCT-guided PCI<br>• Written informed consent prior to any study related procedure	• Patients with any planned PCI within the target vessel(s) within 24 months after the study procedure, other than a planned staged intervention in a second randomized target vessel<br>• Any prior PCI in a non-target vessel within 24 hours before the study procedure, or within previous 30 days if unsuccessful or complicated.<br>• Known hypersensitivity or contraindication to any of the study drugs or radiocontrast dye that cannot be adequately pre-medicated<br>• Prior solid organ transplant which is functioning or active on a waiting list for any solid organ transplants with expected transplantation within 24 months<br>• Immunosuppressant therapy or severe autoimmune disease requiring chronic immunosuppressive therapy<br>• Previous or scheduled radiotherapy to a coronary artery, or the chest/mediastinum<br>• Platelet count <100,000 or >700,000 cells/mm^3<br>• Documented or suspected hepatic disorder<br>• History of bleeding diathesis or coagulopathy, or history of significant gastro-intestinal or significant urinary bleed within the past 6 months<br>• Cerebrovascular accident or transient ischemic attach within the past 6 months, or any prior intracranial bleed, or any permanent neurologic defect, or any known intracranial pathology<br>• Extensive peripheral vascular disease<br>• Patients with life expectancy <2 years for any non-cardiac cause<br>• Current participation in another investigational drug or device clinical study<br>• Pregnancy or nursing or planned pregnancy in the period up to 2 years following index procedure<br>• Other anatomic or comorbid conditions, or other medical, social, or psychological conditions that, in the investigator's opinion, could limit the subject's ability to participate in the clinical investigation or to comply with follow-up requirements, or impact the scientific soundness of the clinical investigation results<br>• Syntax score ≥33, unless a formal meeting of the Heart Team, including a

		• cardiac surgeon, concludes that PCI is appropriate<br>• Planned use of any stent <2.50 mm in a target vessel based on visual estimation.<br>• Planned use of a stent or post-dilatation balloon ≥3.75 mm for the target<br>• Severe vessel tortuosity or calcification in a target vessel such that it is unlikely that the OCT catheter can be delivered<br>• Target vessel including nontarget lesions with diameter stenosis ≥50% that is not planned for treatment at the time of index procedure<br>• Ostial right coronary artery stenosis, is a stent thrombosis<br>• Left main coronary artery<br>• Coronary artery bypass graft<br>• Planned use of any stent other than Xience in a target lesion
**iSIGHT**	• Patients of 18 years of age or older scheduled for PCI of native coronary arteries<br>• Patients with stable angina, non-ST-segment elevation acute coronary syndrome, or ST-segment elevation myocardial infarction ≥48 hours from the initial presentation<br>• Patients with ≥1 target lesion in ≥1 native coronary with a reference diameter ranging from 2.25 to 4.00 mm by visual estimation	• Cardiogenic shock or signs of chronic heart failure<br>• Chronic kidney disease and an estimated glomerular filtration rate ≤45 mL/min per 1.73 m^2.<br>• Left main stenosis ≥50%<br>• Aorto-ostial lesions.<br>• Chronic total occlusion<br>• Bifurcation lesions in which a 2-stent strategy was anticipated<br>• Target lesion in arterial or venous grafts
**IVUS-XPL**	• Age 20 years old or older<br>• Patients with typical chest pain or evidences of myocardial ischemia and positive functional study or reversible changes in the ECG consistent with ischemia<br>• Non-emergent conditions<br>• Patients with signed informed consent<br>• Stent length ≥28 mm by angiography<br>• Significant coronary artery stenosis (>50% by visual estimate) considered for coronary revascularization with stent implantation.<br>• Reference vessel diameter of 2.50 to 4.00 mm by operator assessment.	• Acute ST elevation myocardial infarction within 48 hours.<br>• Contraindication to anti-platelet agents and bleeding history within prior 3 months.<br>• Known hypersensitivity or contraindication to heparin, aspirin, clopidogrel, zotarolimus or other -limus drugs<br>• Prior history of cerebrovascular accident, occlusive peripheral artery disease, thromboembolic disease, stent thrombosis<br>• Age >80 years old<br>• Severe hepatic dysfunction (3x times normal reference values)<br>• Serum creatinine >2.0 mg/dL<br>• Significant leucopenia, neutropenia, thrombocytopenia, anemia, or known bleeding diathesis<br>• Cardiogenic shock

		• Left ventricular ejection fraction <40%<br>• Pregnant women or women with potential childbearing<br>• Life expectancy <1 year<br>• Left main disease requiring PCI<br>• Bifurcation lesion with 2-stent technique<br>• Chronic total occlusion<br>• Presence of previously implanted drug-eluting stent within 6 months<br>• In-stent restenosis
**Kala et al.**	• 18 to 85 years of age<br>• ST-segment elevation myocardial infarction within 12 hours from symptoms onset<br>• Target lesion in a native coronary artery with diameter ranging from 2.50 to 3.75 mm by visual estimation, suitable for stenting	• Signs of chronic heart failure (Killip class IV)<br>• Significant left main stenosis or lesions not suitable for OCT scan (ostial lesion, very distal or vessel >3.75mm in diameter)<br>• Patients with recent (<1 month) bleeding.<br>• Patients with known allergy to aspirin and/or clopidogrel/ticlopidine<br>• Patients in anticoagulant therapy<br>• Patients with life expectancy <1 year<br>• Patients with severe liver disease<br>• Serum creatinine >2.0 mg/dL.<br>• Pregnancy
**Kim et al.**	• Patients of 20 years of age or older<br>• Patients admitted with stable angina or unstable angina<br>• Patients with a single lesion in a single vessel with reference vessel diameter ranging from 2.50 to 3.50 mm and lesion length ≤34 mm and ≤34 mm stent length<br>• Patients able to enforce follow-up angiography	• Target vessel of ST-segment elevation myocardial infarction<br>• Coronary artery bypass grafting<br>• Thrombosis<br>• Restenosis<br>• Bifurcation requiring 2 stents<br>• Lesions requiring overlapped stenting or more than two drug-eluting stents in each vessel<br>• Far distal lesion<br>• Tortuous lesion making difficulties in OCT evaluation or OCT follow-up<br>• Heavy calcified lesions<br>• Chronic total occlusion<br>• Left main disease<br>• Left ventricular ejection fraction <30%<br>• Patients with severe hepatic dysfunction.<br>• Serum creatinine ≥2.0 mg/dL or chronic kidney disease<br>• Life expectancy of less than 1 year.<br>• Patients with reference vessel diameter <2.5 mm or >4.0 mm<br>• Any drug-eluting stent implanted within 3 months at other vessel<br>• Patients with contraindication to antiplatelet agents<br>• Pregnant women or women with potential childbearing

**Li et al.**	• Type 2 diabetes<br>• Stable angina and a positive stress test<br>• PCI for coronary lesions involving a vessel segment with reference vessel diameter between 2.2 and 3.0 mm by using quantitative coronary angiography	• Prior any acute coronary syndrome<br>• Prior PCI or coronary artery bypass grafting of the target vessel<br>• New York Heart Failure class III<br>• Severe hepatic<br>• Severe renal dysfunction or hemodialysis<br>• Impossibility to reach or cross the lesion with the imaging catheter<br>• Contraindication to anticoagulation or high bleeding risk<br>• Life expectancy of less than 2 year
**Liu et al.**	• Age between 18 and 75 years<br>• Unprotected left main lesion planned for receiving drug-eluting stent implantation<br>• Good compliance to post-PCI antiplatelet therapy	• Acute myocardial infarction (≤24 hours)<br>• Cardiogenic shock<br>• High bleeding risk conditions, such as coagulopathy or prior major hemorrhage<br>• Renal failure<br>• Hepatic failure<br>• Carcinoma<br>• Chronic total occlusion in the left anterior descending or left circumflex arteries with no clear access for antegrade treatment or complicated with severe calcification needing rotational atherectomy
**MISTIC-1**	• Over 20 years of age<br>• Stable coronary artery disease with symptoms or myocardial ischemia proven by non-invasive or invasive stress test	• Renal insufficiency with estimated glomerular filtration rate <45 mL/min/1.73 m^2<br>• Left ventricular ejection fraction <30% or history of congestive heart failure<br>• Acute coronary syndrome within 7 days after onset<br>• Target lesion inappropriate for drug-eluting stent implantation or dual antiplatelet therapy for one year after the index procedure<br>• Life expectancy within one year.<br>• Lesion length estimated by quantitative coronary angiography >28 mm.<br>• Chronic total occlusion<br>• Left main lesion<br>• Bifurcation requiring side branch balloon dilatation<br>• Severely calcified lesion<br>• Other conditions making inappropriate to enroll the patients because of safety concern
**OCTACS**	• Age between 18 and 80 years.<br>• Non-ST-segment myocardial infarction	• Patients included in other randomized trials<br>• Left main disease<br>• Bifurcation lesions

	• De novo lesion with ≥50% diameter stenosis by coronary angiography.<br>• PCI with drug-eluting stent	• Life expectancy <1 year<br>• Allergy to aspirin, clopidogrel, ticagrelor and prasugrel<br>• Allergy to limus-agents<br>• Ostial lesions<br>• Serum creatinine>170 µg/l<br>• Tortuous and extremely calcified lesions where intravascular imaging was deemed associated with an increased risk for the patient<br>• Very long lesions
**OCTIVUS**	• 19 years of age or older.<br>• De novo obstructive coronary artery disease undergoing PCI with contemporary drug-eluting stent or restenosis undergoing PCI with contemporary drug-eluting stent or drug-coated balloon<br>• Written informed consent	• ST-segment myocardial infarction.<br>• Estimated glomerular filtration rate <30 mL/min/1.73 m^2, unless on renal replacement therapy<br>• Cardiogenic shock or decompensated heart failure associated with left ventricular ejection fraction <30%<br>• Life expectancy <1 year<br>• Any lesion characteristics resulting in the expected inability to deliver the intracoronary imaging catheter during PCI<br>• Any clinically significant abnormality identified at the screening visit, physical examination, laboratory tests, or electrocardiogram, which in the judgment of the investigator would preclude safe completion of the study<br>• Unwillingness or inability to comply with the procedures described in this protocol
**OCTOBER**	• Stable angina, unstable angina, or clinically stable non-ST-segment elevation myocardial infarction<br>• 18 years of age or older.<br>• Written informed consent and willingness to comply with the specified follow-up contacts<br>• De novo disease<br>• Native coronary bifurcation<br>• Diameter stenosis >50% in the main vessel.<br>• Diameter stenosis >50% in the side branch within 5 mm of the ostium.<br>• Reference size at least 2.75 mm in the main vessel and ≥2.50 mm in the side branch.<br>• Functional significance of the main vessel lesion or documented ischemia of the main vessel territory or other objective documentation of lesion	• Patients with ST-segment elevation myocardial infarction within 72 hours<br>• Patients with cardiogenic shock<br>• Patients with prior coronary artery bypass grafting or planned coronary artery bypass grafting<br>• Patients with renal failure with estimated glomerular filtration rate <50 mL/min/1.73 m^2<br>• Patients with active bleeding or coagulopathy<br>• Patients with expected survival of less than two years<br>• Patients with left ventricular ejection fraction <30%<br>• Patients with New York Heart Association class >II<br>• Patients with relevant allergies (aspirin, clopidogrel, ticagrelor, contrast compounds, everolimus).

	significance. Objective evidence of ischemia is required for all treated lesions except for lesions with diameter stenosis >80% that may be considered significant.<br>• Indication for two-stent technique or one-stent technique with kissing balloon inflation.	• Severe tortuosity around target bifurcation.<br>• Chronic total occlusion.<br>• Left main with massive thrombus<br>• Medina 0,0,1 lesions
**OPINION**	• De novo lesion<br>• Planned drug-eluting stent implantation<br>• Age between 20 and 85 years old<br>• Written informed consent.	• Acute myocardial infarction within 3 months<br>• Cardiogenic shock<br>• Chronic heart failure<br>• Estimated glomerular filtration rate ≤30 ml/min/1.73 m^2 or serum creatinine ≥1.5 mg/dL<br>• Current enrollment in other clinical trial<br>• Planned use of bare metal stent<br>• 3-vessel diseases<br>• Planned surgery within 1 year<br>• Dialysis<br>• Left main stenosis<br>• Aorto-ostial lesion location within 3 mm of the aorta junction<br>• Chronic total occlusion<br>• Reference vessel diameter <2.50 mm in the target segment<br>• Coronary artery bypass grafting
**RENOVATE-COMPLEX-PCI**	• Age ≥19 years old<br>• Coronary artery disease requiring PCI<br>• Complex lesion defined as bifurcation disease involving a side branch (Medina 1,1,1 / 1,0,1 / 0,1,1) ≥2.50 mm, chronic total occlusion, unprotected left main stenosis, long disease (implanted stent ≥38 mm), multi-vessel PCI (≥2 vessels treated at one PCI session), multiple stents needed (≥3 more stent per patient), in-stent restenosis, severe calcification, ostial disease<br>• Verbally-confirmed understanding of risks, benefits and treatment alternatives and written informed consent	• Target lesions not amenable for PCI by operators' decision.<br>• Cardiogenic shock (Killip class IV) at presentation<br>• Intolerance to aspirin, clopidogrel, prasugrel, ticagrelor, heparin, or everolimus<br>• Known true anaphylaxis to contrast medium<br>• Pregnancy or breast feeding<br>• Non-cardiac co-morbid conditions with life expectancy <1 year or that may result in protocol non-compliance<br>• Unwillingness or inability to comply with the procedures described in this protocol
**RESET**	• Patients of 20 years of age or older.<br>• Patients with a de novo lesion requiring a stent 28 mm in length in a vessel with a distal reference diameter 2.50 mm by visual estimation.	• Cardiogenic shock<br>• Left ventricular ejection fraction <40%<br>• ST-segment elevation myocardial infarction within 48 hours after onset of symptoms<br>• Recent (<3 months) bleeding

	• Patients with a lesion.	• Known hypersensitivity to heparin, aspirin, clopidogrel, or a -limus-related drug<br>• Cerebral vascular accident<br>• Peripheral artery occlusive disease<br>• Thromboembolic disease<br>• Stent thrombosis<br>• Bifurcation lesions requiring a 2-stent technique<br>• Chronic total occlusions<br>• History of PCI with drug-eluting stent.
**Tan et al.**	• 70 years or older<br>• Unprotected left main stenosis at least of 50%	• Severe left ventricular ejection fraction <30%<br>• Cardiogenic shock<br>• Acute myocardial infarction<br>• Carcinoma
**ULTIMATE**	• 18 years and older.<br>• Established indication to PCI<br>• Native coronary lesion suitable for drug-eluting stent placement and IVUS imaging<br>• Provision of informed consent prior to any study specific procedures	• ST-segment elevation myocardial infarction within 24 hours from the onset of chest pain to admission<br>• Pregnancy and breast-feeding mother.<br>• Co-morbidity with an estimated life expectancy of <50 % at 12 months.<br>• Scheduled major surgery in the next 12 months.<br>• Inability to follow the protocol and comply with follow-up requirements or any other reason that the investigator feels would place the patient at increased risk.<br>• Previous enrolment in this study or treatment with an investigational drug or device under another study protocol in the past 30 days.<br>• Known allergy against ticagrelor, or against clopidogrel, or aspirin History of major hemorrhage (intracranial, gastrointestinal, etc.).<br>• Not recanalized chronic total occlusion<br>• Severe calcification needing rotational atherectomy<br>• ST-segment elevation myocardial infarction within 24-hour from the onset of chest pain to admission
**Wang et al.**	• ST-segment elevation myocardial infarction within 12 hours of symptom onset<br>• Preprocedural Thrombolysis in Myocardial Infarction flow grade 0 or 1 or thrombus grade ≥3 in the infarct-related artery	• Patients with residual stenosis >75% or Thrombolysis in Myocardial Infarction grade <3 flow after aspiration thrombectomy<br>• Patients with more than 2 stents inserted.<br>• Patients with left main occlusion

	• Critical lesion defined as 50–75% residual stenosis after aspiration thrombectomy and a Thrombolysis in Myocardial Infarction flow grade 3 at the distal end of the infarct-related artery	• Hemodynamic instability requiring hemodynamic support devices<br>• Old myocardial infarction<br>• Prior cardiopulmonary resuscitation<br>• Patients with hepatic and renal dysfunction or neoplastic disease, valvular heart disease, congenital heart disease, or cardiomyopathy<br>• Patients undergoing coronary angioplasty or coronary artery bypass grafting<br>• Patients with coagulation disorders.<br>• Patients with no tolerance for aspirin and clopidogrel<br>• Patients with heparin and contrast medium allergies

IVUS=Intravascular Ultrasound; OCT=Optical Coherence Tomography; PCI=Percutaneous Coronary Intervention.

Online Table 10. Bayesian Random-Effects Network Meta-Analysis by Inconsistency Model and Comparison with Consistency Model.

**Target Lesion Revascularization**					
	ICA	IVUS	OCT	DIC Consistency	DIC UME
ICA		1.53 [1.25-1.87]	1.30 [0.99-1.69]	73.791	72.109
IVUS	0.65 [0.53-0.80]		0.85 [0.63-1.14]		
OCT	0.77 [0.59-1.01]	1.18 [0.88-1.58]			
**Myocardial Infarction**					
	ICA	IVUS	OCT	DIC Consistency	DIC UME
ICA		1.19 [0.88-1.67]	1.08 [0.73-1.54]	71.498	72.357
IVUS	0.84 [0.60-1.14]		1.39 [0.63-3.07]		
OCT	0.92 [0.65-1.37]	0.72 [0.33-1.59]			
**Death**					
	ICA	IVUS	OCT	DIC Consistency	DIC UME
ICA		1.43 [0.94-2.28]	1.35 [0.77-2.17]	67.215	69.279
IVUS	0.70 [0.44-1.07]		1.09 [0.46-2.48]		
OCT	0.74 [0.46-1.30]	0.92 [0.40-2.17]			
**Cardiac Death**					
	ICA	IVUS	OCT	DIC Consistency	DIC UME
ICA		2.09 [1.22-3.86]	1.55 [0.76-2.95]	69.637	70.429
IVUS	0.48 [0.26-0.82]		1.80 [0.46-8.12]		
OCT	0.64 [0.34-1.32]	0.56 [0.12-2.19]			
**Target Vessel Myocardial Infarction**					
	ICA	IVUS	OCT	DIC Consistency	DIC UME
ICA		1.19 [0.87-1.7]	1.16 [0.75-1.72]	70.615	72.252
IVUS	0.84 [0.59-1.15]		1.38 [0.62-3.17]		
OCT	0.86 [0.58-1.34]	0.73 [0.32-1.62]			
**Ischemia-Driven Target Lesion Revascularization**					
	ICA	IVUS	OCT	DIC Consistency	DIC UME
ICA		1.61 [1.22-2.16]	1.05 [0.72-1.52]	73.885	72.424
IVUS	0.62 [0.46-0.82]		1.27 [0.70-2.39]		
OCT	0.95 [0.66-1.4]	0.79 [0.42-1.42]			
**Target Vessel Revascularization**					
	ICA	IVUS	OCT	DIC Consistency	DIC UME
ICA		1.67 [1.28-2.18]	1.03 [0.71-1.43]	70.951	71.298
IVUS	0.60 [0.46-0.78]		0.95 [0.57-1.57]		
OCT	0.97 [0.70-1.41]	1.05 [0.64-1.76]			
**Definite or Probable Stent Thrombosis**					
	ICA	IVUS	OCT	DIC Consistency	DIC UME
ICA		2.19 [1.12-4.65]	1.82 [0.65-4.97]	66.081	66.586
IVUS	0.46 [0.21-0.89]		5.87 [0.60-229.66]		
OCT	0.55 [0.2-1.54]	0.17 [0.00-1.67]			
**Major Adverse Cardiac Events**					
	ICA	IVUS	OCT	DIC Consistency	DIC UME
ICA		1.61 [1.30-1.98]	1.16 [0.86-1.51]	80.240	78.726
IVUS	0.62 [0.5-0.77]		1.25 [0.76-2.05]		
OCT	0.86 [0.66-1.16]	0.80 [0.49-1.31]			

DIC=Deviance Information Criterion; ICA=Invasive Coronary Angiography; IVUS=Intravascular Ultrasound; OCT=Optical Coherence Tomography; UME=Unrelated mean effects.

Values are OR [95% CrIs].

Online Table 11. Frequentist Random-Effects Network Meta-Analysis After Excluding ILUMIEN IV.

**Target Lesion Revascularization**			
	ICA	IVUS	OCT
ICA		1.52 [1.20–1.93]	1.53 [1.05–2.25]
IVUS	0.66 [0.52–0.84]		1.01 [0.69–1.49]
OCT	0.65 [0.45–0.95]	0.99 [0.67–1.46]	
Heterogeneity: I^2=0%; τ^2=0			
**Myocardial Infarction**			
	ICA	IVUS	OCT
ICA		1.08 [0.83–1.41]	1.06 [0.76–1.48]
IVUS	0.92 [0.71–1.21]		0.98 [0.67–1.44]
OCT	0.94 [0.68–1.31]	1.02 [0.70–1.49]	
Heterogeneity: I^2=0%; τ^2=0			
**Death**			
	ICA	IVUS	OCT
ICA		1.31 [0.93–1.84]	1.45 [0.91–2.31]
IVUS	0.77 [0.54–1.08]		1.11 [0.68–1.81]
OCT	0.69 [0.43–1.10]	0.90 [0.55–1.47]	
Heterogeneity: I^2=0%; τ^2=0			
**Cardiac Death**			
	ICA	IVUS	OCT
ICA		1.74 [1.11–2.73]	1.71 [0.95–3.09]
IVUS	0.58 [0.37–0.90]		0.99 [0.50–1.92]
OCT	0.59 [0.32–1.06]	1.02 [0.52–1.98]	
Heterogeneity: I^2=0%; τ^2=0			
**Target Vessel Myocardial Infarction**			
	ICA	IVUS	OCT
ICA		1.10 [0.84–1.44]	1.18 [0.83–1.67]
IVUS	0.91 [0.70–1.20]		1.07 [0.72–1.60]
OCT	0.85 [0.60–1.21]	0.93 [0.63–1.39]	
Heterogeneity: I^2=0%; τ^2=0			
**Ischemia-Driven Target Lesion Revascularization**			
	ICA	IVUS	OCT
ICA		1.56 [1.23–1.99]	1.54 [1.04–2.29]
IVUS	0.64 [0.50–0.81]		0.99 [0.66–1.47]
OCT	0.65 [0.44–0.97]	1.01 [0.68–1.52]	
Heterogeneity: I^2=0%; τ^2=0			
**Target Vessel Revascularization**			
	ICA	IVUS	OCT
ICA		1.61 [1.28–2.01]	1.38 [0.96–1.97]
IVUS	0.62 [0.50–0.78]		0.86 [0.60–1.22]
OCT	0.73 [0.51–1.04]	1.17 [0.82–1.66]	
Heterogeneity: I^2=0%; τ^2=0			
**Definite or Probable Stent Thrombosis**			
	ICA	IVUS	OCT
ICA		1.61 [0.92–2.82]	1.56 [0.66–3.69]
IVUS	0.62 [0.35–1.08]		0.97 [0.37–2.49]
OCT	0.64 [0.27–1.52]	1.04 [0.40–2.68]	
Heterogeneity: I^2=0%; τ^2=0			
**Major Adverse Cardiac Events**			
	ICA	IVUS	OCT
ICA		1.51 [1.26–1.81]	1.46 [1.13–1.89]

IVUS	0.66 [0.55–0.79]		0.97 [0.73–1.27]
OCT	0.68 [0.53–0.89]	1.03 [0.79–1.36]	
Heterogeneity: I^2=0%; τ^2=0			

ICA=Invasive Coronary Angiography; IVUS=Intravascular Ultrasound; OCT=Optical Coherence Tomography.

Values are ORs [95% CIs].

Online Table 12. Bayesian Random-Effects Network Meta-Analysis After Excluding ILUMIEN IV.

**Target Lesion Revascularization**			
	ICA	IVUS	OCT
ICA		1.61 [1.23–2.17]	1.54 [1.01–2.33]
IVUS	0.62 [0.46–0.81]		1.00 [0.66–1.53]
OCT	0.65 [0.43–0.99]	1.00 [0.65–1.50]	
Heterogeneity: I^2=0%; τ^2=0			
**Myocardial Infarction**			
	ICA	IVUS	OCT
ICA		1.12 [0.83–1.56]	1.07 [0.71–1.69]
IVUS	0.89 [0.64–1.20]		1.00 [0.59–1.51]
OCT	0.93 [0.59–1.40]	1.00 [0.66–1.70]	
Heterogeneity: I^2=0%; τ^2=0			
**Death**			
	ICA	IVUS	OCT
ICA		1.41 [0.91–2.17]	1.41 [0.77–2.50]
IVUS	0.71 [0.46–1.10]		1.00 [0.53–1.75]
OCT	0.71 [0.40–1.30]	1.00 [0.57–1.90]	
Heterogeneity: I^2=0%; τ^2=0			
**Cardiac Death**			
	ICA	IVUS	OCT
ICA		1.92 [1.14–3.42]	1.75 [0.83–3.70]
IVUS	0.52 [0.29–0.88]		0.90 [0.39–2.00]
OCT	0.57 [0.27–1.20]	1.10 [0.50–2.60]	
Heterogeneity: I^2=0%; τ^2=0			
**Target Vessel Myocardial Infarction**			
	ICA	IVUS	OCT
ICA		1.14 [0.83–1.61]	1.18 [0.77–1.89]
IVUS	0.88 [0.62–1.20]		1.04 [0.62–1.67]
OCT	0.85 [0.53–1.30]	0.96 [0.60–1.60]	
Heterogeneity: I^2=0%; τ^2=0			
**Ischemia-Driven Target Lesion Revascularization**			
	ICA	IVUS	OCT
ICA		1.64 [1.22–2.18]	1.56 [1.01–2.38]
IVUS	0.61 [0.46–0.82]		1.01 [0.67–1.54]
OCT	0.64 [0.42–0.99]	0.99 [0.65–1.50]	
Heterogeneity: I^2=0%; τ^2=0			
**Target Vessel Revascularization**			
	ICA	IVUS	OCT
ICA		1.64 [1.27–2.10]	1.35 [0.91–2.00]
IVUS	0.61 [0.48–0.79]		1.01 [0.56–1.50]
OCT	0.74 [0.50–1.10]	0.99 [0.65–1.80]	
Heterogeneity: I^2=0%; τ^2=0			
**Definite or Probable Stent Thrombosis**			
	ICA	IVUS	OCT
ICA		1.91 [0.90–4.04]	2.00 [0.67–7.69]
IVUS	0.52 [0.25–1.10]		1.06 [0.32–4.17]
OCT	0.50 [0.13–1.50]	0.94 [0.24–3.10]	
Heterogeneity: I^2=0%; τ^2=0			
**Major Adverse Cardiac Events**			
	ICA	IVUS	OCT
ICA		1.52 [1.24–1.88]	1.39 [1.00–1.88]

IVUS	0.66 [0.53–0.81]		0.91 [0.63–1.27]
OCT	0.72 [0.53–1.00]	1.10 [0.79–1.60]	
Heterogeneity: I^2=0%; τ^2=0			

ICA=Invasive Coronary Angiography; IVUS=Intravascular Ultrasound; OCT=Optical Coherence Tomography.

Values are ORs [95% CrIs].

Online Table 13. Frequentist and Bayesian Rank Probabilities and SUCRA Values After Excluding ILUMIEN IV.

Target Lesion Revascularization			
**Frequentist Ranking**	**ICA**	**IVUS**	**OCT**
**1st**	0.0	45.7	54.3
**2nd**	1.4	54.3	44.3
**3rd**	98.6	0.0	1.4
**SUCRA**	0.8	71.8	77.4
**Bayesian Ranking**	**ICA**	**IVUS**	**OCT**
**1st**	0.0	58.8	41.2
**2nd**	3.3	41.1	55.6
**3rd**	96.7	0.1	3.2
**SUCRA**	1.7	79.3	69.0
**Myocardial Infarction**			
**Frequentist Ranking**	**ICA**	**IVUS**	**OCT**
**1st**	12.5	43.4	44.1
**2nd**	39.4	34.5	26.1
**3rd**	48.1	22.1	29.8
**SUCRA**	31.3	62.8	56.0
**Bayesian Ranking**	**ICA**	**IVUS**	**OCT**
**1st**	10.5	50.6	38.9
**2nd**	37.0	34.8	28.3
**3rd**	52.5	14.7	32.8
**SUCRA**	29.0	67.9	53.0
**Death**			
**Frequentist Ranking**	**ICA**	**IVUS**	**OCT**
**1st**	1.1	35.1	63.8
**2nd**	12.0	58.9	29.1
**3rd**	86.9	6.0	7.1
**SUCRA**	5.3	64.3	80.4
**Bayesian Ranking**	**ICA**	**IVUS**	**OCT**
**1st**	1.3	35.8	63.0
**2nd**	11.2	59.5	29.3
**3rd**	87.5	4.8	7.7
**SUCRA**	6.9	65.5	77.6
**Cardiac Death**			
**Frequentist Ranking**	**ICA**	**IVUS**	**OCT**
**1st**	0.0	53.2	46.8
**2nd**	4.3	46.1	49.6
**3rd**	95.7	0.7	3.6
**SUCRA**	2.2	75.6	72.3
**Bayesian Ranking**	**ICA**	**IVUS**	**OCT**
**1st**	0.2	57.9	41.8
**2nd**	6.7	41.5	51.8
**3rd**	93.1	0.6	6.3
**SUCRA**	3.6	78.7	67.8
**Target Vessel Myocardial Infarction**			
**Frequentist Ranking**	**ICA**	**IVUS**	**OCT**
**1st**	5.0	32.8	62.2
**2nd**	28.9	47.7	23.4
**3rd**	66.1	19.5	14.4
**SUCRA**	21.9	54.3	73.9

Bayesian Ranking	ICA	IVUS	OCT
1st	6.4	40.6	53.0
2nd	28.9	44.3	26.8
3rd	64.7	15.1	20.2
**SUCRA**	20.9	62.7	66.4
**Ischemia-Driven Target Lesion Revascularization**			
**Frequentist Ranking**	**ICA**	**IVUS**	**OCT**
1st	0.0	52.3	47.7
2nd	1.3	47.7	51.0
3rd	98.7	0.0	1.3
**SUCRA**	0.7	76.1	73.3
**Bayesian Ranking**	**ICA**	**IVUS**	**OCT**
1st	0.0	56.1	43.8
2nd	3.1	43.8	53.1
3rd	96.9	0.1	3.1
**SUCRA**	1.60	78.0	70.4
**Target Vessel Revascularization**			
**Frequentist Ranking**	**ICA**	**IVUS**	**OCT**
1st	0.0	80.2	50.1
2nd	4.2	19.8	48.5
3rd	95.8	0.0	1.4
**SUCRA**	2.1	90.7	57.2
**Bayesian Ranking**	**ICA**	**IVUS**	**OCT**
1st	0.0	83.3	16.7
2nd	6.9	16.7	76.4
3rd	93.1	0.0	6.9
**SUCRA**	3.5	91.6	54.9
**Stent Thrombosis**			
**Frequentist Ranking**	**ICA**	**IVUS**	**OCT**
1st	1.2	56.0	42.8
2nd	18.1	40.4	41.5
3rd	80.7	3.6	15.7
**SUCRA**	9.8	74.1	66.1
**Bayesian Ranking**	**ICA**	**IVUS**	**OCT**
1st	0.6	45.7	53.7
2nd	12.6	51.6	35.8
3rd	86.8	2.7	10.8
**SUCRA**	6.9	71.5	71.6
**Major Adverse Cardiac Events**			
**Frequentist Ranking**	**ICA**	**IVUS**	**OCT**
1st	0.0	66.1	33.9
2nd	0.3	33.9	65.8
3rd	99.7	0.0	0.3
**SUCRA**	0.2	82.8	67.1
**Bayesian Ranking**	**ICA**	**IVUS**	**OCT**
1st	0.0	71.0	29.0
2nd	2.7	29.0	68.4
3rd	97.3	0.0	2.6
**SUCRA**	1.3	85.5	63.2

SUCRA=Surface Under the Cumulative Ranking Curve.

Values are percentages.

Online Table 14. Frequentist and Bayesian Network Node Split After Excluding ILUMIEN IV.

		Frequentist		Bayesian	
	W (%)	OR [95 CI]	$P_{inconsistency}$	OR [95 CI]	$P_{inconsistency}$
**Target Lesion Revascularization**					
**IVUS vs ICA**			0.200		0.266
Direct	90	0.63 [0.49–0.80]		0.62 [0.46–0.81]	
Indirect	10	1.05 [0.50–2.22]		1.00 [0.45–2.50]	
Network		0.66 [0.52–0.84]		0.65 [0.50–0.85]	
**OCT vs ICA**			0.126		0.171
Direct	61	0.82 [0.50–1.34]		0.84 [0.48–1.50]	
Indirect	39	0.46 [0.25–0.83]		0.46 [0.23–0.92]	
Network		0.65 [0.45–0.95]		0.65 [0.43–0.99]	
**OCT vs IVUS**			0.132		0.205
Direct	54	0.75 [0.44–1.27]		0.76 [0.43–1.30]	
Indirect	56	1.36 [0.78–2.40]		1.30 [0.70–2.60]	
Network		0.99 [0.67–1.46]		1.00 [0.65–1.50]	
**Myocardial Infarction**					
**IVUS vs ICA**			0.324		0.307
Direct	89	0.88 [0.67–1.17]		0.84 [0.57–1.20]	
Indirect	11	1.36 [0.60–3.05]		1.40 [0.51–4.10]	
Network		0.92 [0.71–1.21]		0.89 [0.64–1.20]	
**OCT vs ICA**			0.324		0.280
Direct	81	1.02 [0.71–1.47]		1.10 [0.63–1.80]	
Indirect	19	0.67 [0.31–1.42]		0.61 [0.24–1.50]	
Network		0.94 [0.68–1.31]		0.93 [0.59–1.40]	
**OCT vs IVUS**			0.440		0.439
Direct	36	0.84 [0.44–1.57]		0.87 [0.43–1.80]	
Indirect	64	1.14 [0.71–1.84]		1.20 [0.63–2.80]	
Network		1.02 [0.70–1.49]		1.00 [0.66–1.70]	
**Death**					
**IVUS vs ICA**			0.965		0.986
Direct	86	0.76 [0.55–1.10]		0.71 [0.43–1.10]	
Indirect	14	0.78 [0.31–1.96]		0.72 [0.22–2.30]	
Network		0.77 [0.54–1.08]		0.71 [0.46–1.10]	
**OCT vs ICA**			0.999		0.887
Direct	66	0.69 [0.39–1.22]		0.72 [0.35–1.60]	
Indirect	34	0.69 [0.31–1.53]		0.66 [0.25–1.80]	
Network		0.69 [0.43–1.10]		0.71 [0.40–1.30]	
**OCT vs IVUS**			0.869		0.796
Direct	49	0.94 [0.47–1.89]		1.10 [0.47–2.90]	
Indirect	51	0.87 [0.44–1.72]		0.93 [0.38–2.60]	
Network		0.90 [0.55–1.47]		1.00 [0.57–1.90]	
**Cardiac Death**					
**IVUS vs ICA**			0.336		0.286
Direct	89	0.53 [0.33–0.86]		0.47 [0.24–0.84]	
Indirect	11	1.09 [0.23–4.35]		1.20 [0.23–7.90]	
Network		0.58 [0.37–0.90]		0.52 [0.29–0.88]	
**OCT vs ICA**			0.359		0.261
Direct	78	0.68 [0.35–1.32]		0.69 [0.29–1.80]	
Indirect	22	0.35 [0.10–1.24]		0.26 [0.01–1.20]	
Network		0.59 [0.32–1.06]		0.57 [0.27–1.20]	

**OCT vs IVUS**			0.463		0.434
Direct	36	0.73 [0.24–2.22]		0.72 [0.18–3.10]	
Indirect	64	1.22 [0.53–2.86]		1.40 [0.49–5.10]	
Network		1.02 [0.52–1.98]		1.10 [0.50–2.60]	
**Target-Vessel Myocardial Infarction**					
**IVUS vs ICA**			0.416		0.430
Direct	89	0.88 [0.66–1.16]		0.84 [0.56–1.20]	
Indirect	11	1.27 [0.55–2.86]		1.30 [0.45–3.80]	
Network		0.91 [0.70–1.20]		0.88 [0.62–1.20]	
**OCT vs ICA**			0.449		0.392
Direct	79	0.91 [0.62–1.35]		0.94 [0.53–1.80]	
Indirect	21	0.65 [0.30–1.41]		0.61 [0.24–1.50]	
Network		0.85 [0.60–1.21]		0.85 [0.53–1.30]	
**OCT vs IVUS**			0.524		0.522
Direct	35	0.78 [0.37–1.54]		0.81 [0.38–1.80]	
Indirect	65	1.03 [0.59–1.70]		1.10 [0.55–2.70]	
Network		0.93 [0.63–1.39]		0.96 [0.60–1.60]	
**Ischemia-Driven Target Lesion Revascularization**					
**IVUS vs ICA**			0.217		0.301
Direct	90	0.63 [0.49–0.80]		0.61 [0.46–0.82]	
Indirect	10	1.03 [0.48–2.22]		1.00 [0.45–2.50]	
Network		0.64 [0.50–0.81]		0.65 [0.50–0.84]	
**OCT vs ICA**			0.137		0.194
Direct	59	0.83 [0.51–1.37]		0.82 [0.47–1.50]	
Indirect	41	0.46 [0.25–0.84]		0.46 [0.23–0.90]	
Network		0.65 [0.44–0.97]		0.64 [0.42–0.99]	
**OCT vs IVUS**			0.144		0.212
Direct	55	0.73 [0.41–1.30]		0.76 [0.42–1.30]	
Indirect	45	1.31 [0.78–2.21]		1.30 [0.67–2.60]	
Network		1.01 [0.68–1.52]		0.99 [0.65–1.50]	
**Target Vessel Revascularization**					
**IVUS vs ICA**			0.516		0.408
Direct	89	0.61 [0.49–0.78]		0.59 [0.45–0.77]	
Indirect	11	0.78 [0.40–1.33]		0.83 [0.45–1.80]	
Network		0.62 [0.50–0.78]		0.61 [0.48–0.79]	
**OCT vs ICA**			0.323		0.403
Direct	54	0.89 [0.55–1.43]		0.87 [0.51–1.60]	
Indirect	46	0.63 [0.38–1.04]		0.62 [0.34–1.10]	
Network		0.73 [0.51–1.04]		0.74 [0.50–1.10]	
**OCT vs IVUS**			0.419		0.399
Direct	61	1.08 [0.69–1.66]		0.76 [0.42–1.80]	
Indirect	39	1.42 [0.83–2.44]		1.30 [0.67–2.90]	
Network		1.17 [0.82–1.66]		0.99 [0.65–1.80]	
**Stent Thrombosis**					
**IVUS vs ICA**			0.274		0.158
Direct	93	0.57 [0.32–1.01]		0.47 [0.21–0.97]	
Indirect	7	1.92 [0.23–16.67]		3.80 [0.22–164.02]	
Network		0.62 [0.35–1.08]		0.52 [0.25–1.10]	
**OCT vs ICA**			0.223		0.992
Direct	79	0.85 [0.32–2.27]		0.79 [0.18–2.90]	
Indirect	21	0.23 [0.04–1.47]		0.08 [0.00–0.86]	
Network		0.64 [0.27–1.52]		0.50 [0.13–1.50]	
**OCT vs IVUS**			0.302		0.391

Direct	33	0.51 [0.10–2.63]		0.43 [0.00–3.30]	
Indirect	67	1.47 [0.46–4.76]		1.30 [0.20–6.70]	
Network		1.04 [0.40–2.68]		0.94 [0.24–3.10]	
**Major Adverse Cardiac Events**					
**IVUS vs ICA**			0.109		0.115
Direct	89	0.63 [0.52–0.78]		0.62 [0.50–0.77]	
Indirect	11	1.00 [0.59–1.70]		1.10 [0.56–2.10]	
Network		0.66 [0.55–0.79]		0.66 [0.53–0.81]	
**OCT vs ICA**			0.054		0.069
Direct	70	0.82 [0.63–1.11]		0.88 [0.61–1.40]	
Indirect	30	0.48 [0.30–0.75]		0.49 [0.28–0.84]	
Network		0.68 [0.53–0.89]		0.72 [0.53–1.00]	
**OCT vs IVUS**			0.088		0.100
Direct	45	1.23 [0.82–1.84]		0.82 [0.52–1.30]	
Indirect	55	0.73 [0.53–1.10]		1.40 [0.89–2.40]	
Network		0.87 [0.72–1.24]		1.10 [0.79–1.60]	

ICA=Invasive Coronary Angiography; IVUS=Intravascular Ultrasound; OCT=Optical Coherence Tomography; $P_{inconsistency}$=P Value for Inconsistency.

Values are OR [95% CIs] in frequentist analysis and OR [95% CrI] in the Bayesian analysis.

Online Table 15. Frequentist Random-Effects Network Meta-Analysis After Excluding OCTOBER.

Target lesion revascularization			
	ICA	IVUS	OCT
ICA		1.44 [1.14–1.82]	1.12 [0.83–1.53]
IVUS	0.70 [0.55–0.88]		0.78 [0.56–1.1]
OCT	0.89 [0.66–1.21]	1.28 [0.91–1.79]	
Heterogeneity: I^2=0%; τ^2=0			
**Myocardial infarction**			
	ICA	IVUS	OCT
ICA		1.11 [0.85–1.45]	1.25 [0.92–1.7]
IVUS	0.90 [0.69–1.17]		1.12 [0.78–1.62]
OCT	0.80 [0.59–1.09]	0.89 [0.62–1.28]	
Heterogeneity: I^2=0%; τ^2=0			
**Death**			
	ICA	IVUS	OCT
ICA		1.28 [0.91–1.79]	1.31 [0.9–1.9]
IVUS	0.78 [0.56–1.09]		1.02 [0.66–1.59]
OCT	0.76 [0.53–1.11]	0.98 [0.63–1.52]	
Heterogeneity: I^2=0%; τ^2=0			
**Cardiac Death**			
	ICA	IVUS	OCT
ICA		1.73 [1.10–2.71]	1.66 [0.93–2.94]
IVUS	0.58 [0.37–0.91]		0.96 [0.50–1.85]
OCT	0.60 [0.34–1.07]	1.04 [0.54–2.02]	
Heterogeneity: I^2=0%; τ^2=0			
**Target Vessel Myocardial Infarction**			
	ICA	IVUS	OCT
ICA		1.11 [0.85–1.46]	1.32 [0.90–1.93]
IVUS	0.90 [0.68–1.18]		1.18 [0.78–1.8]
OCT	0.76 [0.52–1.11]	0.85 [0.56–1.29]	
Heterogeneity: I^2=0%; τ^2=0			
**Ischemia-Driven Target Lesion Revascularization**			
	ICA	IVUS	OCT
ICA		1.44 [1.14–1.82]	1.12 [0.83–1.53]
IVUS	0.70 [0.55–0.88]		0.78 [0.56–1.1]
OCT	0.89 [0.66–1.21]	1.28 [0.91–1.79]	
Heterogeneity: I^2=0%; τ^2=0			
**Target Vessel Revascularization**			
	ICA	IVUS	OCT
ICA		1.51[1.21–1.88]	1.08[0.82–1.41]
IVUS	0.66[0.53–0.82]		0.71[0.53–0.96]
OCT	0.93[0.71–1.22]	1.40 [1.04–1.88]	
Heterogeneity: I^2=0%; τ^2=0			
**Definite or Probable Stent Thrombosis**			
	ICA	IVUS	OCT
ICA		1.70 [0.98–2.97]	2.63 [1.26–5.49]
IVUS	0.59 [0.34–1.02]		1.54 [0.65–3.64]
OCT	0.38 [0.18–0.80]	0.65 [0.27–1.53]	
Heterogeneity: I^2=0%; τ^2=0			
**Major adverse cardiovascular events***			
	ICA	IVUS	OCT
ICA		1.48 [1.23–1.78]	1.26 [0.97–1.63]

IVUS	0.68 [0.56–0.81]		0.85 [0.64–1.12]
OCT	0.80 [0.61–1.03]	1.18 [0.89–1.55]	
Heterogeneity: I^2=0%; τ^2=0.008			

ICA=Invasive Coronary Angiography; IVUS=Intravascular Ultrasound; OCT=Optical Coherence Tomography.

Values are ORs [95% CIs].

Online Table 16. Bayesian Random-Effects Network Meta-Analysis After Excluding OCTOBER.

**Target lesion revascularization**			
	ICA	IVUS	OCT
ICA		1.43 [1.14-1.82]	1.12 [0.89-1.52]
IVUS	0.70 [0.55-0.88]		0.78 [0.56-1.10]
OCT	0.89 [0.66-1.21]	1.28 [0.91-1.79]	
Heterogeneity: I^2=0%; τ^2=0			
**Myocardial infarction**			
	ICA	IVUS	OCT
ICA		1.11 [0.85-1.45]	1.25 [0.92-1.69]
IVUS	0.90 [0.69-1.17]		1.12 [0.78-1.61]
OCT	0.80 [0.59-1.09]	0.89 [0.62-1.28]	
Heterogeneity: I^2=0%; τ^2=0			
**Death**			
	ICA	IVUS	OCT
ICA		1.36 [0.93–2.11]	1.28 [0.73–2.09]
IVUS	0.74 [0.47–1.08]		0.94 [0.5–1.59]
OCT	0.78 [0.48–1.37]	1.06 [0.63–2.01]	
Heterogeneity: I^2=0%; τ^2=0			
**Cardiac Death**			
	ICA	IVUS	OCT
ICA		1.89 [1.14–3.31]	1.71 [0.81–3.53]
IVUS	0.53 [0.30–0.88]		0.91 [0.39–1.98]
OCT	0.58 [0.28–1.23]	1.10 [0.51–2.57]	
Heterogeneity: I^2=0%; τ^2=0			
**Target Vessel Myocardial Infarction**			
	ICA	IVUS	OCT
ICA		1.16 [0.85–1.63]	1.31 [0.8–2.12]
IVUS	0.86 [0.61–1.18]		1.13 [0.66–1.87]
OCT	0.77 [0.47–1.25]	0.89 [0.54–1.52]	
Heterogeneity: I^2=0%; τ^2=0			
**Ischemia-Driven Target Lesion Revascularization**			
	ICA	IVUS	OCT
ICA		1.45 [1.11–1.9]	1.14 [0.79–1.69]
IVUS	0.69 [0.53–0.9]		0.78 [0.53–1.19]
OCT	0.88 [0.59–1.27]	1.28 [0.84–1.89]	
Heterogeneity: I^2=0%; τ^2=0			
**Target Vessel Revascularization**			
	ICA	IVUS	OCT
ICA		1.53 [1.21–1.97]	1.07 [0.76–1.48]
IVUS	0.65 [0.51–0.83]		0.70 [0.49–0.98]
OCT	0.94 [0.68–1.31]	1.44 [1.02–2.05]	
Heterogeneity: I^2=0%; τ^2=0			
**Definite or Probable Stent Thrombosis**			
	ICA	IVUS	OCT
ICA		2.00 [1.03–4.02]	3.19 [1.15–10.55]
IVUS	0.50 [0.25–0.97]		1.59 [0.52–5.50]
OCT	0.31 [0.09–0.87]	0.63 [0.18–1.93]	
Heterogeneity: I^2=0%; τ^2=0			
**Major adverse cardiovascular events***			
	ICA	IVUS	OCT
ICA		1.49 [1.21–1.86]	1.25 [0.91–1.7]

IVUS	0.67 [0.54–0.83]		0.83 [0.60–1.16]
OCT	0.80 [0.59–1.10]	1.20 [0.86–1.67]	
Heterogeneity: I^2=3%			

ICA=Invasive Coronary Angiography; IVUS=Intravascular Ultrasound; OCT=Optical Coherence Tomography.

Values are ORs [95% CrIs].

Online Table 17. Frequentist and Bayesian Rank Probabilities and SUCRA Values After Excluding OCTOBER.

Target Lesion Revascularization			
**Frequentist Ranking**	**ICA**	**IVUS**	**OCT**
**1st**	0.0	92.8	7.2
**2nd**	23.0	7.2	69.8
**3rd**	77.0	0.0	23.0
**SUCRA**	11.15	96.1	42.8
**Bayesian Ranking**	**ICA**	**IVUS**	**OCT**
**1st**	0.3	88.5	11.3
**2nd**	23.2	11.3	65.5
**3rd**	76.5	0.3	23.3
**SUCRA**	11.9	94.1	44.0
**Myocardial Infarction**			
**Frequentist Ranking**	**ICA**	**IVUS**	**OCT**
**1st**	2.6	23.0	74.4
**2nd**	26.1	55.6	18.3
**3rd**	71.3	21.4	7.3
**SUCRA**	16.3	51.7	82.1
**Bayesian Ranking**	**ICA**	**IVUS**	**OCT**
**1st**	3.9	34.8	61.3
**2nd**	22.5	51.5	26.0
**3rd**	73.6	13.7	12.7
**SUCRA**	15.1	60.6	74.3
**Death**			
**Frequentist Ranking**	**ICA**	**IVUS**	**OCT**
**1st**	1.3	46.2	52.5
**2nd**	13.2	46.6	40.2
**3rd**	85.5	7.2	7.3
**SUCRA**	9.3	68.6	72.1
**Bayesian Ranking**	**ICA**	**IVUS**	**OCT**
**1st**	1.9	57.8	40.3
**2nd**	17.6	37.9	44.4
**3rd**	80.5	4.2	15.3
**SUCRA**	10.7	76.8	62.5
**Cardiac Death**			
**Frequentist Ranking**	**ICA**	**IVUS**	**OCT**
**1st**	0.0	53.6	46.4
**2nd**	3.9	45.8	50.3
**3rd**	96.1	0.6	3.3
**SUCRA**	2.5	76.6	71.0
**Bayesian Ranking**	**ICA**	**IVUS**	**OCT**
**1st**	0.2	7.2	92.6
**2nd**	59.7	39.7	0.6
**3rd**	40.1	53.1	6.8
**SUCRA**	3.8	79.5	66.7
**Target Vessel Myocardial Infarction**			
**Frequentist Ranking**	**ICA**	**IVUS**	**OCT**
**1st**	3.0	21.3	75.7
**2nd**	22.3	59.4	18.3
**3rd**	74.7	19.3	6.0
**SUCRA**	15.0	50.6	84.5

Bayesian Ranking	ICA	IVUS	OCT
1st	3.5	22.6	73.9
2nd	29.1	56.1	14.9
3rd	67.4	21.3	11.3
**SUCRA**	14.8	57.1	78.1
**Ischemia-Driven Target Lesion Revascularization**			
**Frequentist Ranking**	**ICA**	**IVUS**	**OCT**
1st	0.2	90.3	9.5
2nd	22.4	9.7	67.9
3rd	77.4	0.0	22.6
**SUCRA**	10.4	96.4	43.3
**Bayesian Ranking**	**ICA**	**IVUS**	**OCT**
1st	0.2	87.9	11.9
2nd	23.5	11.9	64.6
3rd	76.3	0.2	23.5
**SUCRA**	12.0	93.8	44.2
**Target Vessel Revascularization**			
**Frequentist Ranking**	**ICA**	**IVUS**	**OCT**
1st	0.0	99.0	1.0
2nd	29.3	1.0	69.7
3rd	70.7	0.0	29.3
**SUCRA**	14.5	99.2	36.3
**Bayesian Ranking**	**ICA**	**IVUS**	**OCT**
1st	0.1	98.0	1.9
2nd	34.0	1.9	64.0
3rd	65.9	0.0	34.1
**SUCRA**	17.1	99.0	33.9
**Stent Thrombosis**			
**Frequentist Ranking**	**ICA**	**IVUS**	**OCT**
1st	0.0	17.1	82.9
2nd	3.6	80.1	16.3
3rd	96.4	2.8	0.8
**SUCRA**	1.75	56.3	92.0
**Bayesian Ranking**	**ICA**	**IVUS**	**OCT**
1st	0.2	20.0	79.9
2nd	3.2	78.1	18.7
3rd	96.7	2.0	1.4
**SUCRA**	1.8	59.0	89.2
**Major Adverse Cardiac Events**			
**Frequentist Ranking**	**ICA**	**IVUS**	**OCT**
1st	0.0	87.6	12.4
2nd	3.6	12.4	84.0
3rd	96.4	0.0	3.6
**SUCRA**	1.6	93.6	54.9
**Bayesian Ranking**	**ICA**	**IVUS**	**OCT**
1st	0.0	86.6	13.4
2nd	8.0	13.4	78.6
3rd	91.9	0.0	8.0
**SUCRA**	4.1	93.3	52.7

SUCRA=Surface Under the Cumulative Ranking Curve.

Values are percentages.

Online Table 18. Frequentist and Bayesian Network Node Split After Excluding OCTOBER.

		Frequentist		Bayesian	
	W (%)	OR [95 CI]	$P_{inconsistency}$	OR [95 CrI]	$P_{inconsistency}$
**Target Lesion Revascularization**					
**IVUS vs ICA**			0.020		0.030
Direct	87	0.63 [0.49–0.80]		0.62 [0.47–0.81]	
Indirect	13	1.44 [0.75–2.77]		1.50 [0.71–3.32]	
Network		0.70 [0.55–0.88]		0.69 [0.53–0.90]	
**OCT vs ICA**			0.013		0.018
Direct	74	1.12 [0.78–1.59]		1.17 [0.76–1.87]	
Indirect	26	0.46 [0.25–0.84]		0.46 [0.23–0.88]	
Network		0.89 [0.66–1.21]		0.83 [0.63–1.09]	
**OCT vs IVUS**			0.012		0.017
Direct	42	0.76 [0.45–1.29]		0.76 [0.43–1.34]	
Indirect	58	1.84 [1.18–2.36]		1.93 [1.15–3.36]	
Network		1.28 [0.91–1.79]		1.27 [0.84–1.88]	
**Myocardial Infarction**					
**IVUS vs ICA**			0.659		0.539
Direct	89	0.88 [0.67–1.17]		0.84 [0.57–1.16]	
Indirect	11	1.07 [0.48–2.35]		1.15 [0.42–3.30]	
Network		0.90 [0.69–1.17]		0.87 [0.63–1.17]	
**OCT vs ICA**			0.610		0.459
Direct	83	0.83 [0.59–1.16]		0.87 [0.54–1.59]	
Indirect	17	0.67 [0.31–1.42]		0.61 [0.24–1.49]	
Network		0.80 [0.59–1.09]		0.80 [0.53–1.25]	
**OCT vs IVUS**			0.812		0.768
Direct	34	0.84 [0.44–1.57]		0.87 [0.43–1.79]	
Indirect	66	0.92 [0.58–1.44]		0.99 [0.52–2.35]	
Network		0.89 [0.62–1.28]		0.93 [0.60–1.50]	
**Death**					
**IVUS vs ICA**			0.741		0.742
Direct	84	0.76 [0.53–1.10]		0.71 [0.44–1.08]	
Indirect	16	0.89 [0.39–2.05]		0.85 [0.29–2.51]	
Network		0.78 [0.56–1.09]		0.74 [0.47–1.08]	
**OCT vs ICA**			0.779		0.687
Direct	78	0.79 [0.52–1.20]		0.82 [0.45–1.08]	
Indirect	22	0.69 [0.31–1.54]		0.66 [0.25–1.79]	
Network		0.76 [0.53–1.11]		0.78 [0.48–1.38]	
**OCT vs IVUS**			0.891		0.993
Direct	39	0.94 [0.47–1.89]		1.08 [0.49–1.72]	
Indirect	61	1.00 [0.57–1.75]		1.59 [0.51–2.72]	
Network		0.98 [0.63–1.52]		1.06 [0.63–2.01]	
**Cardiac Death**					
**IVUS vs ICA**			0.312		0.258
Direct	89	0.53 [0.33–0.86]		0.47 [0.44–0.83]	
Indirect	11	1.12 [0.29–4.39]		1.28 [0.24–8.03]	
Network		0.58 [0.37–0.91]		0.53 [0.30–0.88]	
**OCT vs ICA**			0.334		0.251
Direct	79	0.70 [0.37–1.33]		0.72 [0.31–1.75]	
Indirect	21	0.35 [0.10–1.23]		0.27 [0.06–1.24]	
Network		0.60 [0.34–1.07]		0.53 [0.28–1.23]	
**OCT vs IVUS**			0.432		0.414

Direct	35	0.73 [0.24–2.22]		0.75 [0.19–3.03]	
Indirect	65	1.27 [0.56–2.86]		1.47 [0.53–5.03]	
Network		1.04 [0.54–2.02]		1.10 [0.51–2.57]	
**Target-Vessel Myocardial Infarction**					
**IVUS vs ICA**			0.633		0.580
Direct	90	0.88 [0.66–1.17]		0.84 [0.55–1.17]	
Indirect	10	1.16 [0.46–2.58]		1.12 [0.39–3.41]	
Network		0.90 [0.68–1.18]		0.86 [0.61–1.18]	
**OCT vs ICA**			0.657		0.576
Direct	75	0.80 [0.63–1.24]		0.83 [0.46–1.62]	
Indirect	25	0.65 [0.30–1.41]		0.61 [0.24–1.50]	
Network		0.76 [0.52–1.11]		0.77 [0.47–1.25]	
**OCT vs IVUS**			0.768		0.716
Direct	39	0.78 [0.40–1.54]		0.81 [0.37–1.81]	
Indirect	61	0.89 [0.52–1.51]		0.97 [0.47–2.43]	
Network		0.85 [0.56–1.29]		0.89 [0.54–1.52]	
**Ischemia-Driven Target Lesion Revascularization**					
**IVUS vs ICA**			0.020		0.029
Direct	87	0.63 [0.49–0.80]		0.62 [0.47–0.82]	
Indirect	13	1.44 [0.75–2.77]		1.50 [0.71–3.28]	
Network		0.70 [0.55–0.88]		0.69 [0.53–0.90]	
**OCT vs ICA**			0.013		0.020
Direct	74	1.12 [0.78–1.59]		1.16 [0.77–1.84]	
Indirect	26	0.46 [0.25–0.84]		0.46 [0.25–0.89]	
Network		0.89 [0.66–1.21]		0.88 [0.59–1.27]	
**OCT vs IVUS**			0.012		0.017
Direct	42	0.76 [0.45–1.28]		0.76 [0.43–1.33]	
Indirect	58	1.85 [1.19–2.86]		1.91 [1.15–3.38]	
Network		1.28 [0.91–1.79]		1.28 [0.84–1.89]	
**Target Vessel Revascularization**					
**IVUS vs ICA**			0.130		0.119
Direct	85	0.62 [0.49–0.78]		0.60 [0.46–0.78]	
Indirect	15	0.99 [0.56–1.73]		1.04 [0.55–2.04]	
Network		0.66[0.53–0.82]		0.65 [0.51–0.83]	
**OCT vs ICA**			0.077		0.079
Direct	72	1.08 [0.78–1.49]		1.14 [0.77–1.83]	
Indirect	28	0.63 [0.38–1.05]		0.62 [0.34–1.11]	
Network		0.93[0.71–1.22]		0.94 [0.68–1.31]	
**OCT vs IVUS**			0.101		0.135
Direct	46	1.08 [0.69–1.67]		1.10 [0.68–1.80]	
Indirect	54	1.75 [1.08–2.63]		1.83 [1.13–3.10]	
Network		1.40 [1.04–1.88]		1.44 [1.02–2.05]	
**Stent Thrombosis**					
**IVUS vs ICA**			0.707		0.333
Direct	92	0.57 [0.32–1.01]		0.47 [0.21–0.94]	
Indirect	8	0.85 [0.11–6.27]		1.95 [0.12–83.02]	
Network		0.59 [0.35–1.02]		0.50 [0.25–0.97]	
**OCT vs ICA**			0.564		0.245
Direct	84	0.42 [0.28–0.93]		0.41 [0.11–1.47]	
Indirect	16	0.23 [0.04–1.48]		0.08 [0.00–0.90]	
Network		0.38 [0.26–0.80]		0.31 [0.10–0.87]	
**OCT vs IVUS**			0.736		0.729
Direct	27	0.51 [0.10–2.63]		0.44 [0.05–3.22]	

Indirect	63	0.71 [0.26–1.92]		0.68 [0.68–3.05]	
Network		0.65 [0.27–1.53]		0.63 [0.18–1.93]	
**Major Adverse Cardiac Events**					
**IVUS vs ICA**			0.028		0.040
Direct	88	0.63 [0.52–0.76]		0.62 [0.50–0.78]	
Indirect	12	1.20 [0.70–2.08]		1.22 [0.66–2.32]	
Network		0.68 [0.56–0.81]		0.67 [0.54–0.83]	
**OCT vs ICA**			0.133		0.025
Direct	81	0.98 [0.72–1.33]		1.00 [0.72–1.47]	
Indirect	19	0.48 [0.29–0.77]		0.48 [0.29–0.83]	
Network		0.80 [0.61–1.03]		0.80 [0.59–1.10]	
**OCT vs IVUS**			0.021		0.028
Direct	35	0.81 [0.54–1.24]		0.82 [0.51–1.27]	
Indirect	65	1.59 [1.09–2.27]		1.62 [1.07–2.60]	
Network		1.18 [0.89–1.55]		1.20 [0.86–1.67]	

ICA=Invasive Coronary Angiography; IVUS=Intravascular Ultrasound; OCT=Optical Coherence Tomography; $P_{inconsistency}$=P Value for Inconsistency.

Values are OR [95% CIs] in frequentist analysis and OR [95% CrI] in the Bayesian analysis.

Online Table 19. Frequentist Random-Effects Network Meta-Analysis by Definition.

Target Lesion Revascularization			
	ICA	IVUS	OCT
ICA		1.44 [1.13–1.85]	1.22 [0.92–1.61]
IVUS	0.69 [0.54–0.89]		0.84 [0.61–1.17]
OCT	0.82 [0.62–1.08]	1.18 [0.85–1.64]	
Heterogeneity: I^2=0%; τ^2=0			
**Myocardial Infarction**			
	ICA	IVUS	OCT
ICA		1.05 [0.78–1.41]	1.15 [0.90–1.47]
IVUS	0.95 [0.71–1.27]		1.09 [0.77–1.55]
OCT	0.87 [0.68–1.11]	0.92 [0.64–1.30]	
Heterogeneity: I^2=0%; τ^2=0.043			
**Death**			
	ICA	IVUS	OCT
ICA		1.16 [0.80–1.67]	1.38 [0.98–1.93]
IVUS	0.87 [0.60–1.24]		1.19 [0.77–1.84]
OCT	0.73 [0.52–1.02]	0.84 [0.54–1.30]	
Heterogeneity: I^2=0%; τ^2=0			
**Cardiac Death**			
	ICA	IVUS	OCT
ICA		1.73 [1.10–2.72]	1.79 [1.10–2.90]
IVUS	0.58 [0.37–0.91]		1.04 [0.56–1.90]
OCT	0.56 [0.34–0.91]	0.97 [0.53–1.77]	
Heterogeneity: I^2=0%; τ^2=0			
**Target Vessel Myocardial Infarction**			
	ICA	IVUS	OCT
ICA		1.09 [0.76–1.56]	1.23 [0.92–1.64]
IVUS	0.92 [0.64–1.31]		1.13 [0.75–1.70]
OCT	0.81 [0.61–1.08]	0.89 [0.59–1.34]	
Heterogeneity: I^2=0%; τ^2=0			
**Ischemia-Driven Target Lesion Revascularization**			
	ICA	IVUS	OCT
ICA		1.39 [0.98–1.96]	1.23 [0.88–1.77]
IVUS	0.72 [0.51–1.02]		0.89 [0.59–1.33]
OCT	0.81 [0.58–1.14]	1.13 [0.75–1.69]	
Heterogeneity: I^2=0%; τ^2=0.030			
**Target Vessel Revascularization**			
	ICA	IVUS	OCT
ICA		1.48 [1.15–1.89]	1.19 [0.92–1.54]
IVUS	0.68 [0.53–0.87]		0.80 [0.59–1.09]
OCT	0.84 [0.65–1.09]	1.24 [0.92–1.69]	
Heterogeneity: I^2=0%; τ^2=0			
**Definite or Probable Stent Thrombosis**			
	ICA	IVUS	OCT
ICA		1.73 [0.96–3.12]	2.10 [1.07–4.10]
IVUS	0.58 [0.32–1.04]		1.21 [0.50–2.89]
OCT	0.48 [0.24–0.93]	0.83 [0.35–1.98]	
Heterogeneity: I^2=0%; τ^2=0			
**Major Adverse Cardiac Events**			
	ICA	IVUS	OCT
ICA		1.43 [1.12–1.84]	1.36 [1.05–1.76]

IVUS	0.70 [0.54–0.89]		0.95 [0.70–1.28]
OCT	0.74 [0.57–0.95]	1.05 [0.78–1.43]	
Heterogeneity: I^2=13.4%; τ^2=0.021			

ICA=Invasive Coronary Angiography; IVUS=Intravascular Ultrasound; OCT=Optical Coherence Tomography.

Values are ORs [95% CIs].

Online Table 20. Bayesian Random-Effects Network Meta-Analysis by Definition.

**Target Lesion Revascularization**			
	ICA	IVUS	OCT
ICA		1.45 [1.09–1.93]	1.24 [0.88–1.78]
IVUS	0.69 [0.52–0.92]		0.86 [0.59–1.28]
OCT	0.81 [0.56–1.14]	1.17 [0.78–1.70]	
Heterogeneity: I^2=0%; τ^2=0			
**Myocardial Infarction**			
	ICA	IVUS	OCT
ICA		1.09 [0.79–1.57]	1.15 [0.81–1.63]
IVUS	0.91 [0.64–1.27]		1.05 [0.67–1.61]
OCT	0.87 [0.61–1.23]	0.95 [0.62–1.48]	
Heterogeneity: I^2=0%; τ^2=0			
**Death**			
	ICA	IVUS	OCT
ICA		1.20 [0.77–1.86]	1.35 [0.81–2.06]
IVUS	0.84 [0.54–1.30]		1.12 [0.63–1.87]
OCT	0.74 [0.49–1.24]	0.89 [0.53–1.59]	
Heterogeneity: I^2=0%; τ^2=0			
**Cardiac Death**			
	ICA	IVUS	OCT
ICA		1.87 [1.13–3.19]	1.87 [1.02–3.19]
IVUS	0.54 [0.31–0.88]		1.00 [0.48–2.03]
OCT	0.53 [0.29–0.98]	1.00 [0.49–2.07]	
Heterogeneity: I^2=0%; τ^2=0			
**Target Vessel Myocardial Infarction**			
	ICA	IVUS	OCT
ICA		1.12 [0.73–1.81]	1.24 [0.82–1.92]
IVUS	0.89 [0.55–1.37]		1.11 [0.65–1.83]
OCT	0.80 [0.52–1.22]	0.90 [0.55–1.54]	
Heterogeneity: I^2=0%; τ^2=0			
**Ischemia-Driven Target Lesion Revascularization**			
	ICA	IVUS	OCT
ICA		1.39 [0.84–2.12]	1.21 [0.74–1.88]
IVUS	0.72 [0.47–1.20]		0.87 [0.53–1.51]
OCT	0.83 [0.53–1.36]	1.15 [0.66–1.90]	
Heterogeneity: I^2=0%; τ^2=0.030			
**Target Vessel Revascularization**			
	ICA	IVUS	OCT
ICA		1.52 [1.14–2.05]	1.23 [0.88–1.79]
IVUS	0.66 [0.49–0.87]		0.81 [0.56–1.18]
OCT	0.82 [0.56–1.14]	1.23 [0.85–1.79]	
Heterogeneity: I^2=0%; τ^2=0			
**Definite or Probable Stent Thrombosis**			
	ICA	IVUS	OCT
ICA		2.03 [0.98–10.06]	2.60 [0.98–4.46]
IVUS	0.49 [0.22–1.04]		1.27 [0.40–5.89]
OCT	0.38 [0.10–1.02]	0.78 [0.40–5.89]	
Heterogeneity: I^2=0%; τ^2=0			
**Major Adverse Cardiac Events**			
	ICA	IVUS	OCT
ICA		1.45 [1.06–1.95]	1.34 [0.96–1.89]
IVUS	0.69 [0.51–0.94]		0.93 [0.64–1.34]

OCT	0.74 [0.53–1.05]	1.08 [0.75–1.56]	
Heterogeneity: I^2=0%; τ^2=0			

ICA=Invasive Coronary Angiography; IVUS=Intravascular Ultrasound; OCT=Optical Coherence Tomography.

Values are OR [95% CrIs].

Online Table 21. Frequentist Random-Effects Network Meta-Analysis with Outcomes Reported by IRR.

**Target Lesion Revascularization**			
	ICA	IVUS	OCT
ICA		1.43 [1.14–1.80]	1.20 [0.92–1.57]
IVUS	0.70 [0.56–0.87]		0.84 [0.61–1.14]
OCT	0.83 [0.64–1.08]	1.19 [0.87–1.63]	
Heterogeneity: I^2=0%; τ^2=0			
**Myocardial infarction**			
	ICA	IVUS	OCT
ICA		1.11 [0.85–1.45]	1.25 [0.92–1.70]
IVUS	0.90 [0.69–1.17]		1.12 [0.78–1.62]
OCT	0.80 [0.59–1.09]	0.89 [0.62–1.28]	
Heterogeneity: I^2=0%; τ^2=0			
**Death**			
	ICA	IVUS	OCT
ICA		1.30 [0.93–1.80]	1.40 [1.01–1.93]
IVUS	0.77 [0.56–1.07]		1.08 [0.72–1.62]
OCT	0.72 [0.52–0.99]	0.93 [0.62–1.39]	
Heterogeneity: I^2=0%; τ^2=0			
**Cardiac Death**			
	ICA	IVUS	OCT
ICA		1.67 [1.08–2.59]	1.70 [1.06–2.73]
IVUS	0.60 [0.39–0.93]		1.02 [0.56–1.83]
OCT	0.59 [0.37–0.94]	0.98 [0.55–1.77]	
Heterogeneity: I^2=0%; τ^2=0			
**Target Vessel Myocardial Infarction**			
	ICA	IVUS	OCT
ICA		1.11 [0.86–1.44]	1.21 [0.92–1.59]
IVUS	0.90 [0.69–1.17]		1.09 [0.77–1.54]
OCT	0.83 [0.63–1.09]	0.92 [0.65–1.31]	
Heterogeneity: I^2=0%; τ^2=0			
**Ischemia-Driven Target Lesion Revascularization**			
	ICA	IVUS	OCT
ICA		1.44 [1.15–1.80]	1.21 [0.92–1.58]
IVUS	0.70 [0.55–0.87]		0.84 [0.61–1.15]
OCT	0.83 [0.63–1.09]	1.19 [0.87–1.63]	
Heterogeneity: I^2=0%; τ^2=0			
**Target Vessel Revascularization**			
	ICA	IVUS	OCT
ICA		1.51 [1.22–1.86]	1.15 [0.90–1.46]
IVUS	0.66 [0.54–0.82]		0.76 [0.58–1.01]
OCT	0.87 [0.69–1.11]	1.31 [0.99–1.73]	
Heterogeneity: I^2=0%; τ^2=0			
**Definite or Probable Stent Thrombosis**			
	ICA	IVUS	OCT
ICA		1.68 [0.97–2.91]	1.97 [1.04–3.71]
IVUS	0.60 [0.34–1.04]		1.17 [0.53–2.58]
OCT	0.51 [0.27–0.96]	0.85 [0.39–1.88]	
Heterogeneity: I^2=0%; τ^2=0			
**Major Adverse Cardiac Events**			
	ICA	IVUS	OCT

ICA		1.44 [1.22–1.70]	1.27 [1.05–1.54]
IVUS	0.69 [0.59–0.82]		0.88 [0.70–1.11]
OCT	0.78 [0.65–0.95]	1.13 [0.90–1.42]	
Heterogeneity: I^2=7%; τ^2=0			

ICA=Invasive Coronary Angiography; IVUS=Intravascular Ultrasound; OCT=Optical Coherence Tomography.

Values are IRRs [95% CIs].

Online Table 22. Bayesian Random-Effects Network Meta-Analysis with Outcomes Reported by IRR.

**Target Lesion Revascularization**			
	ICA	IVUS	OCT
ICA		1.43 [1.10–1.85]	1.23 [0.90–1.73]
IVUS	0.70 [0.54–0.91]		0.86 [0.60–1.25]
OCT	0.81 [0.58–1.11]	1.16 [0.80–1.65]	
Heterogeneity: I^2=0%; τ^2=0			
**Myocardial Infarction**			
	ICA	IVUS	OCT
ICA		1.11 [0.84–1.49]	1.16 [0.85–1.59]
IVUS	0.90 [0.67–1.18]		1.04 [0.71–1.51]
OCT	0.86 [0.63–1.18]	0.96 [0.66–1.42]	
Heterogeneity: I^2=0%; τ^2=0			
**Death**			
	ICA	IVUS	OCT
ICA		1.29 [0.89–1.91]	1.43 [0.94–2.16]
IVUS	0.77 [0.52–1.12]		1.11 [0.67–1.79]
OCT	0.70 [0.46–1.07]	0.90 [0.56–1.49]	
Heterogeneity: I^2=0%; τ^2=0			
**Cardiac Death**			
	ICA	IVUS	OCT
ICA		1.61 [0.93–2.71]	1.89 [1.04–3.54]
IVUS	0.62 [0.37–1.08]		1.18 [0.57–2.54]
OCT	0.53 [0.28–0.96]	0.85 [0.39–1.76]	
Heterogeneity: I^2=0%; τ^2=0			
**Target Vessel Myocardial Infarction**			
	ICA	IVUS	OCT
ICA		1.13 [0.84–1.52]	1.23 [0.88–1.77]
IVUS	0.89 [0.66–1.19]		1.10 [0.73–1.64]
OCT	0.81 [0.57–1.14]	0.91 [0.61–1.37]	
Heterogeneity: I^2=0%; τ^2=0			
**Ischemia-Driven Target Lesion Revascularization**			
	ICA	IVUS	OCT
ICA		1.43 [1.10–1.86]	1.25 [0.91–1.76]
IVUS	0.70 [0.54–0.91]		0.87 [0.61–1.28]
OCT	0.80 [0.57–1.10]	1.15 [0.78–1.65]	
Heterogeneity: I^2=0%; τ^2=0			
**Target Vessel Revascularization**			
	ICA	IVUS	OCT
ICA		1.51 [1.21–1.92]	1.15 [0.87–1.54]
IVUS	0.66 [0.52–0.83]		0.76 [0.55–1.05]
OCT	0.87 [0.65–1.15]	1.31 [0.96–1.80]	
Heterogeneity: I^2=0%; τ^2=0			
**Definite or Probable Stent Thrombosis**			
	ICA	IVUS	OCT
ICA		1.96 [1.00–3.99]	2.02 [0.74–5.35]
IVUS	0.51 [0.25–1.00]		1.03 [0.32–3.15]
OCT	0.49 [0.19–1.34]	0.97 [0.32–3.12]	
Heterogeneity: I^2=0%; τ^2=0			
**Major Adverse Cardiac Events**			
	ICA	IVUS	OCT

ICA		1.45 [1.20–1.76]	1.26 [0.98–1.59]
IVUS	0.69 [0.57–0.83]		0.86 [0.65–1.12]
OCT	0.80 [0.63–1.02]	1.16 [0.89–1.53]	
Heterogeneity: I^2=0%; τ^2=0			

ICA=Invasive Coronary Angiography; IVUS=Intravascular Ultrasound; MACE=Major Adverse Cardiac Events; OCT=Optical Coherence Tomography.

Values are IRRs [95% CrIs]

Online Table 23. Frequentist Random-Effects Network Meta-Analysis at the Longest Available Follow-Up.

**Target Lesion Revascularization**			
	ICA	IVUS	OCT
ICA		1.46 [1.15–1.83]	1.21 [0.92–1.58]
IVUS	0.69 [0.54–0.87]		0.83 [0.60–1.14]
OCT	0.83 [0.63–1.09]	1.21 [0.88–1.66]	
Heterogeneity: I^2=0%; τ^2=0			
**Myocardial Infarction**			
	ICA	IVUS	OCT
ICA		1.15 [0.88–1.51]	1.15 [0.91–1.47]
IVUS	0.87 [0.66–1.14]		1.00 [0.72–1.40]
OCT	0.87 [0.68–1.10]	1.00 [0.71–1.39]	
Heterogeneity: I^2=0%; τ^2=0.043			
**Death**			
	ICA	IVUS	OCT
ICA		1.29 [0.95–1.77]	1.40 [1.01–1.95]
IVUS	0.77 [0.57–1.06]		1.09 [0.72–1.63]
OCT	0.71 [0.51–0.99]	0.92 [0.61–1.38]	
Heterogeneity: I^2=0%; τ^2=0			
**Cardiac Death**			
	ICA	IVUS	OCT
ICA		1.73 [1.15–2.60]	1.72 [1.07–2.78]
IVUS	0.58 [0.38–0.87]		1.00 [0.56–1.78]
OCT	0.58 [0.36–0.93]	1.00 [0.56–1.80]	
Heterogeneity: I^2=0%; τ^2=0			
**Target Vessel Myocardial Infarction**			
	ICA	IVUS	OCT
ICA		1.16 [0.88–1.53]	1.23 [0.93–1.63]
IVUS	0.86 [0.66–1.13]		1.06 [0.74–1.52]
OCT	0.81 [0.61–1.08]	0.94 [0.66–1.35]	
Heterogeneity: I^2=0%; τ^2=0			
**Ischemia-Driven Target Lesion Revascularization**			
	ICA	IVUS	OCT
ICA		1.50 [1.21–1.87]	1.22 [0.93–1.60]
IVUS	0.67 [0.53–0.83]		0.81 [0.59–1.11]
OCT	0.82 [0.62–1.08]	1.23 [0.90–1.69]	
Heterogeneity: I^2=0%; τ^2=0			
**Target Vessel Revascularization**			
	ICA	IVUS	OCT
ICA		1.58 [1.29–1.94]	1.16 [0.91–1.48]
IVUS	0.63 [0.51–0.78]		0.73 [0.55–0.97]
OCT	0.86 [0.67–1.10]	1.36 [1.03–1.80]	
Heterogeneity: I^2=0%; τ^2=0			
**Definite or Probable Stent Thrombosis**			
	ICA	IVUS	OCT
ICA		1.66 [0.95–2.89]	2.04 [1.08–3.85]
IVUS	0.60 [0.35–1.05]		1.23 [0.56–2.71]
OCT	0.49 [0.26–0.92]	0.81 [0.37–1.79]	
Heterogeneity: I^2=0%; τ^2=0			
**Major Adverse Cardiac Events**			
	ICA	IVUS	OCT
ICA		1.53 [1.28–1.83]	1.32 [1.05–1.64]

IVUS	0.66 [0.55–0.78]		0.86 [0.67–1.11]
OCT	0.76 [0.61–0.95]	1.16 [0.90–1.50]	
Heterogeneity: I^2=0%; τ^2=0.010			

ICA=Invasive Coronary Angiography; IVUS=Intravascular Ultrasound; OCT=Optical Coherence Tomography.

Values are ORs [95% CIs].

Online Table 24. Bayesian Random-Effects Network Meta-Analysis at the Longest Available Follow-Up.

**Target Lesion Revascularization**			
	ICA	IVUS	OCT
ICA		1.46 [1.15–1.83]	1.21 [0.92–1.58]
IVUS	0.69 [0.54–0.87]		0.83 [0.60–1.14]
OCT	0.83 [0.63–1.09]	1.21 [0.88–1.66]	
Heterogeneity: I^2=0%; τ^2=0			
**Myocardial Infarction**			
	ICA	IVUS	OCT
ICA		1.15 [0.88–1.51]	1.15 [0.91–1.47]
IVUS	0.87 [0.66–1.14]		1.00 [0.72–1.40]
OCT	0.87 [0.68–1.10]	1.00 [0.71–1.39]	
Heterogeneity: I^2=0%; τ^2=0.043			
**Death**			
	ICA	IVUS	OCT
ICA		1.29 [0.95–1.77]	1.40 [1.01–1.95]
IVUS	0.77 [0.57–1.06]		1.09 [0.72–1.63]
OCT	0.71 [0.51–0.99]	0.92 [0.61–1.38]	
Heterogeneity: I^2=0%; τ^2=0			
**Cardiac Death**			
	ICA	IVUS	OCT
ICA		1.73 [1.15–2.60]	1.72 [1.07–2.78]
IVUS	0.58 [0.38–0.87]		1.00 [0.56–1.78]
OCT	0.58 [0.36–0.93]	1.00 [0.56–1.80]	
Heterogeneity: I^2=0%; τ^2=0			
**Target Vessel Myocardial Infarction**			
	ICA	IVUS	OCT
ICA		1.16 [0.88–1.53]	1.23 [0.93–1.63]
IVUS	0.86 [0.66–1.13]		1.06 [0.74–1.52]
OCT	0.81 [0.61–1.08]	0.94 [0.66–1.35]	
Heterogeneity: I^2=0%; τ^2=0			
**Ischemia-Driven Target Lesion Revascularization**			
	ICA	IVUS	OCT
ICA		1.50 [1.21–1.87]	1.22 [0.93–1.60]
IVUS	0.67 [0.53–0.83]		0.81 [0.59–1.11]
OCT	0.82 [0.62–1.08]	1.23 [0.90–1.69]	
Heterogeneity: I^2=0%; τ^2=0			
**Target Vessel Revascularization**			
	ICA	IVUS	OCT
ICA		1.58 [1.29–1.94]	1.16 [0.91–1.48]
IVUS	0.63 [0.51–0.78]		0.73 [0.55–0.97]
OCT	0.86 [0.67–1.10]	1.36 [1.03–1.80]	
Heterogeneity: I^2=0%; τ^2=0			
**Definite or Probable Stent Thrombosis**			
	ICA	IVUS	OCT
ICA		1.66 [0.95–2.89]	2.04 [1.08–3.85]
IVUS	0.60 [0.35–1.05]		1.23 [0.56–2.71]
OCT	0.49 [0.26–0.92]	0.81 [0.37–1.79]	
Heterogeneity: I^2=0%; τ^2=0			
**Major Adverse Cardiac Events**			
	ICA	IVUS	OCT
ICA		1.53 [1.28–1.83]	1.32 [1.05–1.64]

IVUS	0.66 [0.55–0.78]		0.86 [0.67–1.11]
OCT	0.76 [0.61–0.95]	1.16 [0.90–1.50]	
Heterogeneity: I^2=0%; τ^2=0.010			

ICA=Invasive Coronary Angiography; IVUS=Intravascular Ultrasound; OCT=Optical Coherence Tomography.

Values are ORs [95% CrIs].

Online Table 25. Frequentist Random-Effects Network Meta-Analysis After Excluding Trials with Higher Risk of Bias.

**Target Lesion Revascularization**			
	ICA	IVUS	OCT
ICA		1.44 [1.11–1.86]	1.20 [0.91–1.59]
IVUS	0.70 [0.54–0.90]		0.84 [0.60–1.17]
OCT	0.83 [0.63–1.09]	1.19 [0.86–1.66]	
Heterogeneity: I^2=0%; τ^2=0.001			
**Myocardial Infarction**			
	ICA	IVUS	OCT
ICA		1.06 [0.80–1.41]	1.14 [0.90–1.46]
IVUS	0.94 [0.71–1.25]		1.08 [0.76–1.52]
OCT	0.87 [0.69–1.12]	0.93 [0.66–1.31]	
Heterogeneity: I^2=0%; τ^2=0			
**Death**			
	ICA	IVUS	OCT
ICA		1.30 [0.92–1.84]	1.41 [1.01–1.95]
IVUS	0.77 [0.54–1.09]		1.08 [0.71–1.65]
OCT	0.71 [0.51–0.99]	0.93 [0.61–1.41]	
Heterogeneity: I^2=0%; τ^2=0			
**Cardiac Death**			
	ICA	IVUS	OCT
ICA		1.83 [1.13–2.98]	1.74 [1.08–2.82]
IVUS	0.55 [0.34–0.88]		0.95 [0.51–1.77]
OCT	0.57 [0.36–0.93]	1.05 [0.57–1.96]	
Heterogeneity: I^2=0%; τ^2=0			
**Target Vessel Myocardial Infarction**			
	ICA	IVUS	OCT
ICA		1.07 [0.80–1.42]	1.21 [0.92–1.61]
IVUS	0.94 [0.70–1.25]		1.14 [0.79–1.64]
OCT	0.82 [0.62–1.09]	0.88 [0.61–1.27]	
Heterogeneity: I^2=0%; τ^2=0			
**Ischemia-Driven Target Lesion Revascularization**			
	ICA	IVUS	OCT
ICA		1.44 [1.11–1.87]	1.21 [0.91–1.61]
IVUS	0.70 [0.54–0.90]		0.84 [0.60–1.18]
OCT	0.83 [0.62–1.10]	1.19 [0.85–1.66]	
Heterogeneity: I^2=0%; τ^2=0.003			
**Target Vessel Revascularization**			
	ICA	IVUS	OCT
ICA		1.50 [1.18–1.90]	1.14 [0.89–1.46]
IVUS	0.67 [0.53–0.85]		0.76 [0.57–1.02]
OCT	0.88 [0.68–1.12]	1.31 [0.98–1.76]	
Heterogeneity: I^2=0%; τ^2=0			
**Definite or Probable Stent Thrombosis**			
	ICA	IVUS	OCT
ICA		1.78 [0.91–3.48]	2.06 [1.09–3.90]
IVUS	0.56 [0.29–1.10]		1.16 [0.49–2.73]
OCT	0.48 [0.26–0.92]	0.86 [0.37–2.03]	
Heterogeneity: I^2=0%; τ^2=0.001			
**Major Adverse Cardiac Events**			
	ICA	IVUS	OCT
ICA		1.47 [1.2–1.81]	1.30 [1.05–1.63]

IVUS	0.68 [0.55–0.83]		0.88 [0.68–1.15]
OCT	0.77 [0.62–0.96]	1.13 [0.87–1.47]	
Heterogeneity: I^2=7%; τ^2=0.009			

ICA=Invasive Coronary Angiography; IVUS=Intravascular Ultrasound; OCT=Optical Coherence Tomography.

Values are ORs [95% CIs].

Online Table 26. Bayesian Random-Effects Network Meta-Analysis After Excluding Trials with Higher Risk of Bias.

Target Lesion Revascularization			
	ICA	IVUS	OCT
ICA		1.44 [1.05–1.94]	1.21 [0.86–1.74]
IVUS	0.69 [0.52–0.95]		0.84 [0.58–1.28]
OCT	0.82 [0.57–1.16]	1.19 [0.78–1.73]	
Heterogeneity: I^2=0%; τ^2=0			
**Myocardial Infarction**			
	ICA	IVUS	OCT
ICA		1.09 [0.80–1.54]	1.14 [0.80–1.59]
IVUS	0.91 [0.65–1.25]		1.04 [0.67–1.56]
OCT	0.88 [0.63–1.24]	0.96 [0.64–1.49]	
Heterogeneity: I^2=0%; τ^2=0			
**Death**			
	ICA	IVUS	OCT
ICA		1.39 [0.94–2.17]	1.39 [0.87–2.12]
IVUS	0.72 [0.46–1.07]		1.00 [0.56–1.63]
OCT	0.72 [0.47–1.15]	1.00 [0.61–1.78]	
Heterogeneity: I^2=0%; τ^2=0			
**Cardiac Death**			
	ICA	IVUS	OCT
ICA		2.00 [1.15–3.70]	1.79 [0.94–3.24]
IVUS	0.50 [0.27–0.87]		0.90 [0.40–1.82]
OCT	0.56 [0.31–1.06]	1.11 [0.55–2.51]	
Heterogeneity: I^2=0%; τ^2=0			
**Target Vessel Myocardial Infarction**			
	ICA	IVUS	OCT
ICA		1.10 [0.8–1.58]	1.22 [0.84–1.77]
IVUS	0.91 [0.63–1.25]		1.10 [0.69–1.69]
OCT	0.82 [0.56–1.20]	0.91 [0.59–1.44]	
Heterogeneity: I^2=0%; τ^2=0			
**Ischemia-Driven Target Lesion Revascularization**			
	ICA	IVUS	OCT
ICA		1.44 [1.05–1.95]	1.23 [0.87–1.76]
IVUS	0.70 [0.51–0.95]		0.85 [0.58–1.29]
OCT	0.81 [0.57–1.15]	1.17 [0.78–1.74]	
Heterogeneity: I^2=0%; τ^2=0			
**Target Vessel Revascularization**			
	ICA	IVUS	OCT
ICA		1.51 [1.15–1.99]	1.13 [0.82–1.54]
IVUS	0.66 [0.50–0.87]		0.75 [0.52–1.06]
OCT	0.88 [0.65–1.22]	1.33 [0.95–1.91]	
Heterogeneity: I^2=0%; τ^2=0			
**Definite or Probable Stent Thrombosis**			
	ICA	IVUS	OCT
ICA		2.23 [0.93–6.1]	2.43 [0.89–8.43]
IVUS	0.45 [0.16–1.07]		1.10 [0.32–4.14]
OCT	0.41 [0.12–1.13]	0.91 [0.24–3.17]	
Heterogeneity: I^2=0%; τ^2=0			
**Major Adverse Cardiac Events**			
	ICA	IVUS	OCT
ICA		1.49 [1.17–1.89]	1.29 [0.97–1.69]

IVUS	0.67 [0.53–0.85]		0.87 [0.63–1.18]
OCT	0.77 [0.59–1.03]	1.15 [0.85–1.59]	
Heterogeneity: I^2=7%; τ^2=0			

ICA=Invasive Coronary Angiography; IVUS=Intravascular Ultrasound; OCT=Optical Coherence Tomography.

Values are OR [95% CrIs].

Online Table 27. Frequentist Random-Effects Network Meta-Analysis After Excluding Trials Without Primary Clinical Endpoints to be Assessed at Mid- or Long-Term Follow-Up.

Target Lesion Revascularization			
	ICA	IVUS	OCT
ICA		1.46 [1.15–1.83]	1.21 [0.92–1.58]
IVUS	0.69 [0.54–0.87]		0.83 [0.60–1.14]
OCT	0.83 [0.63–1.09]	1.21 [0.88–1.66]	
Heterogeneity: I^2=0%; τ^2=0			
**Myocardial Infarction**			
	ICA	IVUS	OCT
ICA		1.15 [0.88–1.51]	1.15 [0.91–1.47]
IVUS	0.87 [0.66–1.14]		1.00 [0.72–1.40]
OCT	0.87 [0.68–1.10]	1.00 [0.71–1.39]	
Heterogeneity: I^2=0%; τ^2=0.043			
**Death**			
	ICA	IVUS	OCT
ICA		1.29 [0.95–1.77]	1.40 [1.01–1.95]
IVUS	0.77 [0.57–1.06]		1.09 [0.72–1.63]
OCT	0.71 [0.51–0.99]	0.92 [0.61–1.38]	
Heterogeneity: I^2=0%; τ^2=0			
**Cardiac Death**			
	ICA	IVUS	OCT
ICA		1.73 [1.15–2.60]	1.72 [1.07–2.78]
IVUS	0.58 [0.38–0.87]		1.00 [0.56–1.78]
OCT	0.58 [0.36–0.93]	1.00 [0.56–1.80]	
Heterogeneity: I^2=0%; τ^2=0			
**Target Vessel Myocardial Infarction**			
	ICA	IVUS	OCT
ICA		1.16 [0.88–1.53]	1.23 [0.93–1.63]
IVUS	0.86 [0.66–1.13]		1.06 [0.74–1.52]
OCT	0.81 [0.61–1.08]	0.94 [0.66–1.35]	
Heterogeneity: I^2=0%; τ^2=0			
**Ischemia-Driven Target Lesion Revascularization**			
	ICA	IVUS	OCT
ICA		1.50 [1.21–1.87]	1.22 [0.93–1.60]
IVUS	0.67 [0.53–0.83]		0.81 [0.59–1.11]
OCT	0.82 [0.62–1.08]	1.23 [0.90–1.69]	
Heterogeneity: I^2=0%; τ^2=0			
**Target Vessel Revascularization**			
	ICA	IVUS	OCT
ICA		1.58 [1.29–1.94]	1.16 [0.91–1.48]
IVUS	0.63 [0.51–0.78]		0.73 [0.55–0.97]
OCT	0.86 [0.67–1.10]	1.36 [1.03–1.80]	
Heterogeneity: I^2=0%; τ^2=0			
**Definite or Probable Stent Thrombosis**			
	ICA	IVUS	OCT
ICA		1.66 [0.95–2.89]	2.04 [1.08–3.85]
IVUS	0.60 [0.35–1.05]		1.23 [0.56–2.71]
OCT	0.49 [0.26–0.92]	0.81 [0.37–1.79]	
Heterogeneity: I^2=0%; τ^2=0			
**Major Adverse Cardiac Events**			

	ICA	IVUS	OCT
ICA		1.53 [1.28–1.83]	1.32 [1.05–1.64]
IVUS	0.66 [0.55–0.78]		0.86 [0.67–1.11]
OCT	0.76 [0.61–0.95]	1.16 [0.90–1.50]	
Heterogeneity: I^2=0%; τ^2=0.010			

ICA=Invasive Coronary Angiography; IVUS=Intravascular Ultrasound; OCT=Optical Coherence Tomography.

Values are ORs [95% CIs].

Online Table 28. Bayesian Random-Effects Network Meta-Analysis After Excluding Trials Without Primary Clinical Endpoints to be Assessed at Mid- or Long-Term Follow-Up.

**Target Lesion Revascularization**			
	ICA	IVUS	OCT
ICA		1.50 [1.09-2.10]	1.26 [0.85-1.95]
IVUS	0.66 [0.48-0.92]		0.84 [0.54-1.34]
OCT	0.80 [0.51-1.18]	1.20 [0.74-1.86]	
Heterogeneity: I^2=0%; τ^2=0			
**Myocardial Infarction**			
	ICA	IVUS	OCT
ICA		1.20 [0.83-1.87]	1.19 [0.77-1.90]
IVUS	0.84 [0.54-1.21]		1.00 [0.57-1.66]
OCT	0.84 [0.53-1.29]	1.00 [0.60-1.76]	
Heterogeneity: I^2=15%; τ^2=0			
**Death**			
	ICA	IVUS	OCT
ICA		1.31 [0.86-2.06]	1.46 [0.85-2.37]
IVUS	0.76 [0.49-1.17]		1.11 [0.59-1.92]
OCT	0.69 [0.42-1.18]	0.90 [0.52-1.71]	
Heterogeneity: I^2=0%; τ^2=0			
**Cardiac Death**			
	ICA	IVUS	OCT
ICA		1.68 [0.93-3.11]	2.04 [0.98-4.37]
IVUS	0.59 [0.32-1.07]		1.21 [0.52-2.96]
OCT	0.49 [0.23-1.02]	0.83 [0.34-1.91]	
Heterogeneity: I^2=0%; τ^2=0			
**Target Vessel Myocardial Infarction**			
	ICA	IVUS	OCT
ICA		1.22 [0.84-1.89]	1.27 [0.81-2.01]
IVUS	0.82 [0.53-1.19]		1.04 [0.59-1.73]
OCT	0.79 [0.50-1.23]	0.96 [0.58-1.68]	
Heterogeneity: I^2=14%; τ^2=0			
**Ischemia-Driven Target Lesion Revascularization**			
	ICA	IVUS	OCT
ICA		1.22 [0.84-1.89]	1.27 [0.81-2.01]
IVUS	0.82 [0.53-1.19]		1.04 [0.59-1.73]
OCT	0.79 [0.50-1.23]	0.96 [0.58-1.68]	
Heterogeneity: I^2=0%; τ^2=0			
**Target Vessel Revascularization**			
	ICA	IVUS	OCT
ICA		1.53 [1.14-2.07]	1.22 [0.86-1.82]
IVUS	0.65 [0.48-0.88]		0.80 [0.54-1.20]
OCT	0.82 [0.55-1.17]	1.25 [0.83-1.86]	
Heterogeneity: I^2=2%; τ^2=0			
**Definite or Probable Stent Thrombosis**			
	ICA	IVUS	OCT
ICA		1.73 [0.88-3.65]	2.34 [0.94-6.67]
IVUS	0.58 [0.27-1.14]		1.35 [0.46-4.25]
OCT	0.43 [0.15-1.07]	0.74 [0.24-2.17]	
Heterogeneity: I^2=1%; τ^2=0			
**Major Adverse Cardiac Events**			
	ICA	IVUS	OCT

ICA		1.55 [1.19-2.03]	1.37 [0.97-1.93]
IVUS	0.65 [0.49-0.84]		0.88 [0.60-1.28]
OCT	0.73 [0.52-1.03]	1.13 [0.78-1.66]	
Heterogeneity: I^2=0%; τ^2=0.002			

ICA=Invasive Coronary Angiography; IVUS=Intravascular Ultrasound; OCT=Optical Coherence Tomography.

Values are OR [95% CrIs].

Online Table 29. Frequentist Random-Effects Network Meta-Analysis After Excluding Trials with a Sample Size <100 Patients.

**Target Lesion Revascularization**			
	ICA	IVUS	OCT
ICA		1.45 [1.15–1.83]	1.20 [0.92–1.58]
IVUS	0.69 [0.55–0.87]		0.83 [0.60–1.14]
OCT	0.83 [0.63–1.09]	1.20 [0.88–1.65]	
Heterogeneity: I^2=0%; τ^2=0			
**Myocardial Infarction**			
	ICA	IVUS	OCT
ICA		1.11 [0.85–1.44]	1.15 [0.90–1.46]
IVUS	0.90 [0.69–1.18]		1.04 [0.75–1.45]
OCT	0.87 [0.68–1.11]	0.96 [0.69–1.34]	
Heterogeneity: I^2=0%; τ^2=0			
**Death**			
	ICA	IVUS	OCT
ICA		1.30 [0.93–1.82]	1.41 [1.01–1.95]
IVUS	0.77 [0.55–1.07]		1.08 [0.71–1.64]
OCT	0.71 [0.51–0.99]	0.92 [0.61–1.40]	
Heterogeneity: I^2=0%; τ^2=0			
**Cardiac Death**			
	ICA	IVUS	OCT
ICA		1.74 [1.11–2.72]	1.73 [1.07–2.79]
IVUS	0.57 [0.37–0.90]		0.99 [0.54–1.81]
OCT	0.58 [0.36–0.94]	1.01 [0.55–1.84]	
Heterogeneity: I^2=0%; τ^2=0			
**Target Vessel Myocardial Infarction**			
	ICA	IVUS	OCT
ICA		1.11 [0.85–1.46]	1.22 [0.92–1.62]
IVUS	0.90 [0.69–1.18]		1.10 [0.77–1.57]
OCT	0.82 [0.62–1.08]	0.91 [0.64–1.30]	
Heterogeneity: I^2=0%; τ^2=0			
**Ischemia-Driven Target Lesion Revascularization**			
	ICA	IVUS	OCT
ICA		1.45 [1.15–1.83]	1.21 [0.92–1.59]
IVUS	0.69 [0.55–0.87]		0.83 [0.61–1.15]
OCT	0.83 [0.63–1.09]	1.20 [0.87–1.65]	
Heterogeneity: I^2=0%; τ^2=0			
**Target Vessel Revascularization**			
	ICA	IVUS	OCT
ICA		1.53 [1.23–1.90]	1.15 [0.90–1.47]
IVUS	0.66 [0.53–0.82]		0.75 [0.57–1.00]
OCT	0.87 [0.68–1.11]	1.33 [1.00–1.77]	
Heterogeneity: I^2=0%; τ^2=0			
**Definite or Probable Stent Thrombosis**			
	ICA	IVUS	OCT
ICA		1.65 [0.93–2.92]	2.04 [1.08–3.85]
IVUS	0.61 [0.34–1.07]		1.24 [0.56–2.75]
OCT	0.49 [0.26–0.92]	0.81 [0.36–1.80]	
Heterogeneity: I^2=0%; τ^2=0			
**Major Adverse Cardiac Events**			
	ICA	IVUS	OCT
ICA		1.49 [1.24–1.78]	1.30 [1.06–1.60]

IVUS	0.67 [0.56–0.80]		0.88 [0.69–1.12]
OCT	0.77 [0.63–0.94]	1.14 [0.90–1.45]	
Heterogeneity: I^2=0%; τ^2=0.002			

ICA=Invasive Coronary Angiography; IVUS=Intravascular Ultrasound; OCT=Optical Coherence Tomography.

Values are ORs [95% CIs].

Online Table 30. Bayesian Random-Effects Network Meta-Analysis After Excluding Trials with a Sample Size <100 Patients.

**Target Lesion Revascularization**			
	ICA	IVUS	OCT
ICA		1.45 [1.11–1.90]	1.22 [0.88–1.73]
IVUS	0.69 [0.53–0.90]		0.84 [0.58–1.23]
OCT	0.82 [0.58–1.14]	1.19 [0.81–1.72]	
Heterogeneity: I^2=0%; τ^2=0			
**Myocardial Infarction**			
	ICA	IVUS	OCT
ICA		1.15 [0.86–1.57]	1.15 [0.83–1.59]
IVUS	0.87 [0.64–1.16]		1.00 [0.66–1.48]
OCT	0.87 [0.63–1.20]	1.00 [0.68–1.51]	
Heterogeneity: I^2=0%; τ^2=0			
**Death**			
	ICA	IVUS	OCT
ICA		1.39 [0.96–2.08]	1.41 [0.88–2.11]
IVUS	0.72 [0.48–1.04]		1.01 [0.58–1.62]
OCT	0.72 [0.47–1.13]	0.99 [0.62–1.73]	
Heterogeneity: I^2=0%; τ^2=0			
**Cardiac Death**			
	ICA	IVUS	OCT
ICA		1.90 [1.14–3.30]	1.77 [0.97–3.20]
IVUS	0.53 [0.30–0.87]		0.93 [0.44–1.88]
OCT	0.57 [0.31–1.03]	1.08 [0.53–2.27]	
Heterogeneity: I^2=0%; τ^2=0			
**Target Vessel Myocardial Infarction**			
	ICA	IVUS	OCT
ICA		1.16 [0.86–1.63]	1.23 [0.86–1.79]
IVUS	0.86 [0.61–1.16]		1.07 [0.68–1.60]
OCT	0.81 [0.56–1.16]	0.94 [0.62–1.47]	
Heterogeneity: I^2=0%; τ^2=0			
**Ischemia-Driven Target Lesion Revascularization**			
	ICA	IVUS	OCT
ICA		1.46 [1.12–1.90]	1.23 [0.88–1.74]
IVUS	0.68 [0.53–0.89]		1.19 [0.80–1.73]
OCT	0.81 [0.57–1.13]	0.84 [0.58–1.25]	
Heterogeneity: I^2=0%; τ^2=0			
**Target Vessel Revascularization**			
	ICA	IVUS	OCT
ICA		1.54 [1.21–1.97]	1.14 [0.85–1.53]
IVUS	0.65 [0.51–0.82]		0.74 [0.53–1.02]
OCT	0.88 [0.65–1.18]	1.35 [0.98–1.90]	
Heterogeneity: I^2=0%; τ^2=0			
**Definite or Probable Stent Thrombosis**			
	ICA	IVUS	OCT
ICA		1.91 [0.94–4.04]	2.70 [1.12–8.81]
IVUS	0.52 [0.94–4.04]		1.41 [0.50–5.18]
OCT	0.37 [0.11–0.90]	0.71 [0.19–2.10]	
Heterogeneity: I^2=0%; τ^2=0			
**Major Adverse Cardiac Events**			
	ICA	IVUS	OCT
ICA		1.47 [1.19–1.82]	1.29 [0.99–1.67]

IVUS	0.67 [0.56–0.80]		0.88 [0.65–1.18]
OCT	0.78 [0.60–1.01]	1.14 [0.85–1.54]	
Heterogeneity: I^2=0%; τ^2=0.002			

ICA=Invasive Coronary Angiography; IVUS=Intravascular Ultrasound; OCT=Optical Coherence Tomography.

Values are OR [95% CrIs].

Online Table 31. Frequentist Random-Effects Network Meta-Analysis After Excluding Trials Employing IVI only for Stent Optimization.

**Target Lesion Revascularization**			
	ICA	IVUS	OCT
ICA		1.55 [1.15-2.10]	1.30 [0.94-1.79]
IVUS	0.64 [0.48-0.87]		0.84 [0.58-1.21]
OCT	0.77 [0.56-1.06]	1.20 [0.82-1.74]	
Heterogeneity: I^2=0%; τ^2=0.022			
**Myocardial Infarction**			
	ICA	IVUS	OCT
ICA		1.15 [0.80-1.66]	1.16 [0.91-1.49]
IVUS	0.87 [0.60-1.24]		1.01 [0.68-1.49]
OCT	0.86 [0.67-1.10]	0.99 [0.67-1.48]	
Heterogeneity: I^2=0%; τ^2=0			
**Death**			
	ICA	IVUS	OCT
ICA		1.34 [0.91-1.97]	1.42 [1.02-1.99]
IVUS	0.75 [0.51-1.10]		1.06 [0.68-1.66]
OCT	0.70 [0.50-0.98]	0.94 [0.60-1.47]	
Heterogeneity: I^2=0%; τ^2=0			
**Cardiac Death**			
	ICA	IVUS	OCT
ICA		1.85 [1.10-3.11]	1.79 [1.09-2.92]
IVUS	0.54 [0.32-0.91]		0.96 [0.51-1.83]
OCT	0.56 [0.34-0.92]	1.04 [0.55-1.98]	
Heterogeneity: I^2=0%; τ^2=0			
**Target Vessel Myocardial Infarction**			
	ICA	IVUS	OCT
ICA		1.17 [0.81-1.70]	1.24 [0.93-1.66]
IVUS	0.85 [0.59-1.24]		1.06 [0.70-1.61]
OCT	0.80 [0.60-1.07]	0.94 [0.62-1.43]	
Heterogeneity: I^2=0%; τ^2=0			
**Ischemia-Driven Target Lesion Revascularization**			
	ICA	IVUS	OCT
ICA		1.55 [1.15-2.10]	1.30 [0.94-1.79]
IVUS	0.64 [0.48-0.87]		0.84 [0.58-1.21]
OCT	0.77 [0.56-1.06]	1.20 [0.82-1.74]	
Heterogeneity: I^2=0%; τ^2=0.015			
**Target Vessel Revascularization**			
	ICA	IVUS	OCT
ICA		1.59 [1.23-2.04]	1.18 [0.92-1.52]
IVUS	0.63 [0.49-0.81]		0.74 [0.55-1.00]
OCT	0.85 [0.66-1.09]	1.34 [1.00-1.81]	
Heterogeneity: I^2=0%; τ^2=0			
**Definite or Probable Stent Thrombosis**			
	ICA	IVUS	OCT
ICA		1.79 [0.82-3.91]	2.07 [1.05-4.11]
IVUS	0.56 [0.26-1.22]		1.16 [0.45-2.97]
OCT	0.48 [0.24-0.96]	0.86 [0.34-2.22]	
Heterogeneity: I^2=0%; τ^2=0			
**Major Adverse Cardiac Events**			
	ICA	IVUS	OCT
ICA		1.62 [1.30-2.02]	1.34 [1.09-1.64]

IVUS	0.62 [0.50-0.77]		0.83 [0.64-1.07]
OCT	0.75 [0.61-0.92]	1.21 [0.93-1.57]	
Heterogeneity: I^2=0%; τ^2=0.001			

ICA=Invasive Coronary Angiography; IVUS=Intravascular Ultrasound; OCT=Optical Coherence Tomography.

Values are OR [95% CIs].

Online Table 32. Bayesian Random-Effects Network Meta-Analysis After Excluding Trials Employing IVI only for Stent Optimization.

**Target Lesion Revascularization**			
	ICA	IVUS	OCT
ICA		1.60 [1.13-2.30]	1.31 [0.91-2.02]
IVUS	0.62 [0.43-0.88]		0.82 [0.54-1.30]
OCT	0.76 [0.50-1.10]	1.22 [0.77-1.87]	
Heterogeneity: I^2=3%; τ^2=0			
**Myocardial Infarction**			
	ICA	IVUS	OCT
ICA		1.19 [0.79-1.81]	1.17 [0.81-1.71]
IVUS	0.84 [0.55-1.27]		0.99 [0.61-1.57]
OCT	0.85 [0.58-1.23]	1.01 [0.64-1.63]	
Heterogeneity: I^2=0%; τ^2=0			
**Death**			
	ICA	IVUS	OCT
ICA		1.47 [0.92-2.63]	1.41 [0.82-2.26]
IVUS	0.68 [0.38-1.09]		0.96 [0.47-1.65]
OCT	0.71 [0.44-1.23]	1.04 [0.61-2.12]	
Heterogeneity: I^2=0%; τ^2=0			
**Cardiac Death**			
	ICA	IVUS	OCT
ICA		2.00 [1.09-3.92]	1.84 [0.95-3.43]
IVUS	0.50 [0.26-0.92]		0.92 [0.39-1.98]
OCT	0.54 [0.29-1.05]	1.09 [0.51-2.54]	
Heterogeneity: I^2=0%; τ^2=0			
**Target Vessel Myocardial Infarction**			
	ICA	IVUS	OCT
ICA		1.21 [0.79-1.90]	1.27 [0.84-1.92]
IVUS	0.8 3[0.53-1.27]		1.05 [0.62-1.74]
OCT	0.79 [0.52-1.19]	0.96 [0.57-1.61]	
Heterogeneity: I^2=0%; τ^2=0			
**Ischemia-Driven Target Lesion Revascularization**			
	ICA	IVUS	OCT
ICA		1.60 [1.14-2.28]	1.32 [0.90-2.04]
IVUS	0.63 [0.44-0.87]		0.83 [0.54-1.30]
OCT	0.76 [0.49-1.11]	1.21 [0.77-1.84]	
Heterogeneity: I^2=3%; τ^2=0			
**Target Vessel Revascularization**			
	ICA	IVUS	OCT
ICA		1.64 [1.23-2.25]	1.20 [0.87-1.68]
IVUS	0.61 [0.44-0.81]		0.73[0.50-1.04]
OCT	0.84 [0.59-1.15]	1.37 [0.96-1.99]	
Heterogeneity: I^2=0%; τ^2=0			
**Definite or Probable Stent Thrombosis**			
	ICA	IVUS	OCT
ICA		1.90 [0.70-5.07]	2.17 [0.67-6.99]
IVUS	0.53 [0.20-1.42]		1.14 [0.31-4.41]
OCT	0.46 [0.14-1.50]	0.87 [0.23-3.25]	
Heterogeneity: I^2=0%; τ^2=0			
**Major Adverse Cardiac Events**			
	ICA	IVUS	OCT
ICA		1.63 [1.25-2.13]	1.35[1.02-1.80]

IVUS	0.61 [0.47-0.80]		0.83 [0.60-1.15]
OCT	0.74 [0.56-0.98]	1.21 [0.87-1.68]	
Heterogeneity: I^2=0%; τ^2=0			

ICA=Invasive Coronary Angiography; IVUS=Intravascular Ultrasound; OCT=Optical Coherence Tomography.

Values are OR [95% CrIs].

Online Table 33. Frequentist Random-Effects Network Meta-Analysis After Excluding EROSION III.

**Target Lesion Revascularization**			
	ICA	IVUS	OCT
ICA		1.46 [1.15-1.85]	1.24 [0.93-1.66]
IVUS	0.68 [0.54-0.87]		0.85 [0.61-1.18]
OCT	0.81 [0.60-1.08]	1.18 [0.85-1.64]	
Heterogeneity: I^2=0%; τ^2=0			
**Myocardial Infarction**			
	ICA	IVUS	OCT
ICA		1.10 [0.84-1.43]	1.15 [0.90-1.46]
IVUS	0.91 [0.70-1.19]		1.05 [0.75-1.46]
OCT	0.87 [0.68-1.11]	0.95 [0.69-1.33]	
Heterogeneity: I^2=0%; τ^2=0			
**Death**			
	ICA	IVUS	OCT
ICA		1.30 [0.93-1.82]	1.41 [1.01-1.97]
IVUS	0.77 [0.55-1.07]		1.08 [0.71-1.65]
OCT	0.71 [0.51-0.99]	0.92 [0.61-1.40]	
Heterogeneity: I^2=0%; τ^2=0			
**Cardiac Death**			
	ICA	IVUS	OCT
ICA		1.75[1.11-2.74]	1.78 [1.07-2.95]
IVUS	0.57 [0.37-0.90]		1.02 [0.55-1.89]
OCT	0.56 [0.34-0.93]	0.98 [0.53-1.82]	
Heterogeneity: I^2=0%; τ^2=0			
**Target Vessel Myocardial Infarction**			
	ICA	IVUS	OCT
ICA		1.10 [0.84-1.44]	1.22 [0.92-1.62]
IVUS	0.91 [0.69-1.19]		1.11 [0.77-1.58]
OCT	0.82 [0.62-1.09]	0.90 [0.63-1.29]	
Heterogeneity: I^2=0%; τ^2=0			
**Ischemia-Driven Target Lesion Revascularization**			
	ICA	IVUS	OCT
ICA		1.46 [1.16-1.85]	1.23 [0.93-1.63]
IVUS	0.68 [0.54-0.86]		0.84 [0.61-1.17]
OCT	0.81 [0.61-1.07]	1.19 [0.86-1.64]	
Heterogeneity: I^2=0%; τ^2=0			
**Target Vessel Revascularization**			
	ICA	IVUS	OCT
ICA		1.54 [1.24-1.91]	1.17 [0.91-1.50]
IVUS	0.65 [0.52-0.81]		0.76 [0.57-1.01]
OCT	0.86 [0.67-1.10]	1.32 [0.99-1.76]	
Heterogeneity: I^2=0%; τ^2=0			
**Definite or Probable Stent Thrombosis**			
	ICA	IVUS	OCT
ICA		1.66 [0.95-2.89]	2.04 [1.08-3.85]
IVUS	0.60 [0.35-1.05]		1.23 [0.56-2.71]
OCT	0.49 [0.26-0.92]	0.81 [0.37-1.79]	
Heterogeneity: I^2=0%; τ^2=0			
**Major Adverse Cardiac Events**			
	ICA	IVUS	OCT

ICA		1.50 [1.25-1.79]	1.34 [1.09-1.66]
IVUS	0.67 [0.56-0.80]		0.90 [0.70-1.15]
OCT	0.74 [0.60-0.92]	1.11 [0.87-1.43]	
Heterogeneity: I^2=0%; τ^2=0.003			

ICA=Invasive Coronary Angiography; IVUS=Intravascular Ultrasound; OCT=Optical Coherence Tomography.

Values are ORs [95% CIs].

Online Table 34. Frequentist and Rank Probabilities and SUCRA Values After Excluding EROSION III.

Target Lesion Revascularization			
Frequentist Ranking	ICA	IVUS	OCT
1st	0.0	82.0	18.0
2nd	7.8	17.8	74.4
3rd	92.2	0.2	7.6
SUCRA	3.60	92.2	54.3
**Myocardial Infarction**			
Frequentist Ranking	ICA	IVUS	OCT
1st	4.8	36.7	58.5
2nd	29.7	39.6	30.7
3rd	65.5	23.7	10.8
SUCRA	18.0	58.3	73.8
**Death**			
Frequentist Ranking	ICA	IVUS	OCT
1st	0.1	34.8	65.1
2nd	8.0	59.4	32.6
3rd	91.9	5.8	2.3
SUCRA	3.9	68.6	81.0
**Cardiac Death**			
Frequentist Ranking	ICA	IVUS	OCT
1st	0.0	45.6	54.5
2nd	2.2	54.0	43.8
3rd	97.8	0.4	1.8
SUCRA	1.1	74.5	74.4
**Target Vessel Myocardial Infarction**			
Frequentist Ranking	ICA	IVUS	OCT
1st	3.1	30.2	66.7
2nd	27.3	47.5	25.2
3rd	69.6	22.3	8.1
SUCRA	17.5	51.3	81.3
**Ischemia-Driven Target Lesion Revascularization**			
Frequentist Ranking	ICA	IVUS	OCT
1st	0.0	85.0	15.0
2nd	8.5	15.0	76.5
3rd	91.5	0.0	8.5
SUCRA	3.2	92.3	54.6
**Target Vessel Revascularization**			
Frequentist Ranking	ICA	IVUS	OCT
1st	0.0	96.9	3.1
2nd	11.1	3.1	85.8
3rd	88.9	0.0	11.1
SUCRA	7.0	98.3	44.8
**Stent Thrombosis**			
Frequentist Ranking	ICA	IVUS	OCT
1st	0.1	28.7	71.2
2nd	4.7	67.6	27.7
3rd	95.2	3.7	1.1
SUCRA	2.4	62.3	85.4
**Major Adverse Cardiac Events**			

Frequentist Ranking	ICA	IVUS	OCT
**1st**	0.0	81.4	18.6
**2nd**	0.3	18.6	81.1
**3rd**	99.7	0.0	0.3
**SUCRA**	0.45	89.0	60.6

SUCRA=Surface Under the Cumulative Ranking Curve.

Values are percentages.

Online Table 35. Bayesian Random-Effects Network Meta-Analysis After Excluding EROSION III.

Target Lesion Revascularization			
	ICA	IVUS	OCT
ICA		1.48 [1.13-1.93]	1.26 [0.90-1.81]
IVUS	0.68 [0.52-0.88]		0.85 [0.59-1.27]
OCT	0.79 [0.55-1.12]	1.17 [0.79-1.7]	
Heterogeneity: I^2=0%; τ^2=0			
**Myocardial Infarction**			
	ICA	IVUS	OCT
ICA		1.13 [0.85-1.54]	1.15 [0.82-1.59]
IVUS	0.88 [0.65-1.17]		1.01 [0.67-1.49]
OCT	0.87 [0.63-1.22]	0.99 [0.67-1.49]	
Heterogeneity: I^2=0%; τ^2=0			
**Death**			
	ICA	IVUS	OCT
ICA		1.38 [0.95-2.08]	1.40 [0.87-2.15]
IVUS	0.72 [0.48-1.05]		1.02 [0.57-1.65]
OCT	0.71 [0.47-1.15]	0.98 [0.60-1.75]	
Heterogeneity: I^2=0%; τ^2=0			
**Cardiac Death**			
	ICA	IVUS	OCT
ICA		1.91 [1.16-3.33]	1.84 [0.95-3.57]
IVUS	0.52 [0.30-0.86]		0.97 [0.43-2.00]
OCT	0.54 [0.28-1.05]	1.03 [0.50-2.33]	
Heterogeneity: I^2=0%; τ^2=0			
**Target Vessel Myocardial Infarction**			
	ICA	IVUS	OCT
ICA		1.14 [0.85-1.58]	1.23 [0.86-1.75]
IVUS	0.87 [0.63-1.17]		1.08 [0.69-1.62]
OCT	0.81 [0.57-1.16]	0.93 [0.62-1.44]	
Heterogeneity: I^2=0%; τ^2=0			
**Ischemia-Driven Target Lesion Revascularization**			
	ICA	IVUS	OCT
ICA		1.48 [1.14-1.94]	1.27 [0.91-1.83]
IVUS	0.67 [0.51-0.88]		0.86 [0.59-1.27]
OCT	0.79[0.55-1.10]	1.17 [0.79-1.70]	
Heterogeneity: I^2=0%; τ^2=0			
**Target Vessel Revascularization**			
	ICA	IVUS	OCT
ICA		1.57 [1.23-2.01]	1.17 [0.86-1.60]
IVUS	0.64 [0.50-0.81]		0.75 [0.53-1.04]
OCT	0.86 [0.63-1.17]	1.34 [0.96-1.88]	
Heterogeneity: I^2=0%; τ^2=0			
**Definite or Probable Stent Thrombosis**			
	ICA	IVUS	OCT
ICA		1.93 [1.00-3.85]	2.32 [0.98-6.13]
IVUS	0.52 [0.26-1.00]		1.20 [0.44-3.55]
OCT	0.43 [0.16-1.02]	0.83 [0.28-2.29]	
Heterogeneity: I^2=0%; τ^2=0			
**Major Adverse Cardiac Events**			
	ICA	IVUS	OCT
ICA		1.51 [1.23-1.86]	1.35 [1.03-1.78]

IVUS	0.66 [0.54-0.81]		0.89 [0.66-1.21]
OCT	0.74 [0.56-0.97]	1.12 [0.83-1.51]	
Heterogeneity: I^2=2%; τ^2=0.003			

ICA=Invasive Coronary Angiography; IVUS=Intravascular Ultrasound; OCT=Optical Coherence Tomography.

Values are ORs [95% CIs].

Online Table 36. Bayesian and Rank Probabilities and SUCRA Values After Excluding EROSION III.

Target Lesion Revascularization			
Frequentist Ranking	ICA	IVUS	OCT
1st	0.1	79.8	20.0
2nd	8.7	19.9	71.4
3rd	91.2	0.3	8.5
SUCRA	4.5	89.8	55.8
**Myocardial Infarction**			
Frequentist Ranking	ICA	IVUS	OCT
1st	5.1	45.1	49.9
2nd	28.7	37.8	33.5
3rd	66.3	17.1	16.7
SUCRA	19.4	64.0	66.6
**Death**			
Frequentist Ranking	ICA	IVUS	OCT
1st	0.8	47.1	52.1
2nd	10.0	48.8	41.2
3rd	89.2	4.1	6.7
SUCRA	5.8	71.5	72.7
**Cardiac Death**			
Frequentist Ranking	ICA	IVUS	OCT
1st	0.1	53.9	46.0
2nd	3.8	45.5	50.7
3rd	96.1	0.6	3.3
SUCRA	2.0	76.6	71.4
**Target Vessel Myocardial Infarction**			
Frequentist Ranking	ICA	IVUS	OCT
1st	2.9	34.8	62.2
2nd	23.6	48.4	28.0
3rd	73.4	16.8	9.8
SUCRA	14.8	59.0	76.3
**Ischemia-Driven Target Lesion Revascularization**			
Frequentist Ranking	ICA	IVUS	OCT
1st	0.1	79.5	20.4
2nd	8.1	20.3	71.6
3rd	91.8	0.2	8.0
SUCRA	4.1	89.6	56.2
**Target Vessel Revascularization**			
Frequentist Ranking	ICA	IVUS	OCT
1st	0.0	96.0	4.0
2nd	14.9	4.0	81.1
3rd	85.1	0.0	14.9
SUCRA	7.5	98.0	44.6
**Stent Thrombosis**			
Frequentist Ranking	ICA	IVUS	OCT
1st	0.2	35.3	64.5.4
2nd	4.8	62.4	32.9
3rd	95.0	2.4	2.6
SUCRA	2.6	66.5	80.9
**Major Adverse Cardiac Events**			

Frequentist Ranking	ICA	IVUS	OCT
**1st**	0.0	77.9	22.1
**2nd**	1.6	22.1	76.3
**3rd**	98.4	0.0	1.6
**SUCRA**	0.82	88.9	60.2

SUCRA=Surface Under the Cumulative Ranking Curve.

Values are percentages.

Online Table 37. Bayesian Random-Effects Network Meta-Regression Analysis by Diabetes.

**Target Lesion Revascularization**			
	ICA	IVUS	OCT
ICA		1.47 [1.10-1.92]	1.21 [0.87-1.75]
IVUS	0.68 [0.52-0.91]		0.83 [0.57-1.25]
OCT	0.83 [0.57-1.15]	1.21 [0.80-1.77]	
Regressor estimate [95% CrI]: 0.98 [0.91–1.05]			
**Myocardial Infarction**			
	ICA	IVUS	OCT
ICA		1.15 [0.86-1.56]	1.16 [0.83-1.59]
IVUS	0.87 [0.64-1.16]		1.01 [0.66-1.54]
OCT	0.86 [0.63-1.21]	0.99 [0.65-1.52]	
Regressor estimate [95% CrI]: 1.02 [0.94–1.11]			
**Death**			
	ICA	IVUS	OCT
ICA		1.37 [0.95-2.08]	1.35 [0.84-2.08]
IVUS	0.73 [0.48-1.05]		0.97 [0.54-1.59]
OCT	0.74 [0.48-1.19]	1.03 [0.63-1.84]	
Regressor estimate [95% CrI]: 0.96 [0.86–1.06]			
**Cardiac Death**			
	ICA	IVUS	OCT
ICA		1.92 [1.15-3.49]	1.75 [0.94-3.22]
IVUS	0.52 [0.29-0.87]		0.90 [0.42-1.89]
OCT	0.57 [0.31-1.07]	1.11 [0.53-2.36]	
Regressor estimate [95% CrI]: 0.98 [0.87–1.11]			
**Target Vessel Myocardial Infarction**			
	ICA	IVUS	OCT
ICA		1.15 [0.86-1.56]	1.24 [0.87-1.75]
IVUS	0.87 [0.64-1.17]		1.06 [0.66-1.64]
OCT	0.81 [0.57-1.15]	0.94 [0.61-1.51]	
Regressor estimate [95% CrI]: 1.02 [0.94–1.12]			
**Ischemia-Driven Target Lesion Revascularization**			
	ICA	IVUS	OCT
ICA		1.47 [1.11-1.92]	1.21 [0.88-1.75]
IVUS	0.68 [0.52-0.90]		0.82 [0.55-1.22]
OCT	0.83 [0.57-1.14]	1.22 [0.82-1.82]	
Regressor estimate [95% CrI]: 0.97 [0.89–1.05]			
**Target Vessel Revascularization**			
	ICA	IVUS	OCT
ICA		1.56 [1.22-1.96]	1.15 [0.85-1.54]
IVUS	0.64 [0.51-0.82]		0.72 [0.51-1.00]
OCT	0.90 [0.66-1.22]	1.39 [1.00-1.96]	
Regressor estimate [95% CrI]: 0.98 [0.91–1.05]			
**Definite or Probable Stent Thrombosis**			
	ICA	IVUS	OCT
ICA		1.89 [0.97-3.57]	2.27 [0.95-5.88]
IVUS	0.53 [0.28-1.03]		1.22 [0.41-3.67]
OCT	0.44 [0.17-1.05]	0.82 [0.27-2.44]	
Regressor estimate [95% CrI]: 0.92 [0.78–1.09]			
**Major Adverse Cardiac Events**			
	ICA	IVUS	OCT
ICA		1.52 [1.22-1.85]	1.28 [0.99-1.67]

IVUS	0.66 [0.54-0.82]		0.86 [0.63-1.18]
OCT	0.78 [0.60-1.01]	1.16 [0.85-1.60]	
Regressor estimate [95% CrI]: 0.99 [0.94–1.05]			

ICA=Invasive Coronary Angiography; IVUS=Intravascular Ultrasound; OCT=Optical Coherence Tomography.

Values are ORs [95% CrIs].

Online Table 38. Bayesian Random-Effects Network Meta-Regression Analysis by Acute Coronary Syndrome.

**Target Lesion Revascularization**			
	ICA	IVUS	OCT
ICA		1.59 [1.18-2.13]	1.27 [0.92-1.75]
IVUS	0.63 [0.47-0.85]		0.81 [0.56-1.18]
OCT	0.79 [0.57-1.09]	1.23 [0.85-1.79]	
Regressor estimate [95% CrI]: 1.05 [0.96–1.17]			
**Myocardial Infarction**			
	ICA	IVUS	OCT
ICA		1.11 [0.82-1.59]	1.14 [0.80-1.59]
IVUS	0.90 [0.63-1.22]		1.02 [0.66-1.56]
OCT	0.88 [0.63-1.25]	0.98 [0.64-1.52]	
Regressor estimate [95% CrI]: 0.99 [0.91–1.08]			
**Death**			
	ICA	IVUS	OCT
ICA		1.37 [0.89-2.13]	1.35 [0.82-2.13]
IVUS	0.73 [0.47-1.12]		0.99 [0.56-1.59]
OCT	0.74 [0.47-1.22]	1.01 [0.63-1.79]	
Regressor estimate [95% CrI]: 0.98 [0.84–1.14]			
**Cardiac Death**			
	ICA	IVUS	OCT
ICA		1.92 [1.09-3.46]	1.75 [0.90-3.33]
IVUS	0.52 [0.29-0.92]		0.93 [0.42-1.92]
OCT	0.57 [0.30-1.11]	1.08 [0.52-2.41]	
Regressor estimate [95% CrI]: 1.00 [0.80–1.21]			
**Target Vessel Myocardial Infarction**			
	ICA	IVUS	OCT
ICA		1.16 [0.83-1.67]	1.22 [0.83-1.79]
IVUS	0.86 [0.60-1.21]		1.06 [0.66-1.67]
OCT	0.82 [0.56-1.20]	0.94 [0.60-1.52]	
Regressor estimate [95% CrI]: 1.00 [0.90–1.13]			
**Ischemia-Driven Target Lesion Revascularization**			
	ICA	IVUS	OCT
ICA		1.56 [1.16-2.22]	1.28 [0.93-1.82]
IVUS	0.64 [0.48-0.86]		0.82 [0.57-1.19]
OCT	0.78 [0.55-1.08]	1.22 [0.84-1.77]	
Regressor estimate [95% CrI]: 1.05 [0.95–1.17]			
**Target Vessel Revascularization**			
	ICA	IVUS	OCT
ICA		1.59 [1.22-2.08]	1.16 [0.84-1.56]
IVUS	0.63 [0.48-0.82]		0.74 [0.51-1.02]
OCT	0.86 [0.64-1.19]	1.36 [0.98-1.95]	
Regressor estimate (95% CrI): 1.01 [0.92–1.11]			
**Definite or Probable Stent Thrombosis**			
	ICA	IVUS	OCT
ICA		1.64 [0.81-3.45]	2.14 [0.91-5.42]
IVUS	0.61 [0.29-1.24]		1.28 [0.46-3.74]
OCT	0.47 [0.19-1.09]	0.78 [0.27-2.17]	
Regressor estimate [95% CrI]: 0.87 [0.65–1.14]			
**Major Adverse Cardiac Events**			
	ICA	IVUS	OCT
ICA		1.56 [1.25-1.96]	1.32 [1.02-1.70]

IVUS	0.64 [0.51-0.80]		0.84 [0.62-1.11]
OCT	0.76 [0.59-0.98]	1.19 [0.90-1.62]	
Regressor estimate [95% CrI]: 1.04 [0.97–1.13]			

ICA=Invasive Coronary Angiography; IVUS=Intravascular Ultrasound; OCT=Optical Coherence Tomography.

Values are ORs [95% CrIs].

Online Table 39. Bayesian Random-Effects Network Meta-Regression Analysis by Stent Length.

Target Lesion Revascularization			
	ICA	IVUS	OCT
ICA		1.53 [1.14-2.05]	1.38 [0.76-2.41]
IVUS	0.65 [0.49-0.88]		0.91 [0.53-1.54]
OCT	0.73 [0.42-1.32]	1.11 [0.65-1.88]	
Regressor estimate [95% CrI]: 1.04 [0.89–1.21]			
**Myocardial Infarction**			
	ICA	IVUS	OCT
ICA		1.19 [0.85-1.73]	1.21 [0.65-2.29]
IVUS	0.84 [0.58-1.17]		1.03 [0.55-1.82]
OCT	0.83 [0.44-1.54]	0.97 [0.55-1.80]	
Regressor estimate [95% CrI]: 1.01 [0.87–1.19]			
**Death**			
	ICA	IVUS	OCT
ICA		1.35 [0.86-2.16]	1.41 [0.66-2.89]
IVUS	0.74 [0.46-1.16]		1.04 [0.53-1.92]
OCT	0.71 [0.35-1.52]	0.96 [0.52-1.88]	
Regressor estimate [95% CrI]: 0.98 [0.80–1.20]			
**Cardiac Death**			
	ICA	IVUS	OCT
ICA		2.01 [1.16-3.71]	1.90 [0.71-4.90]
IVUS	0.50 [0.27-0.86]		0.93 [0.38-2.20]
OCT	0.53 [0.20-1.42]	1.07 [0.45-2.67]	
Regressor estimate [95% CrI]: 1.02 [0.77–1.30]			
**Target Vessel Myocardial Infarction**			
	ICA	IVUS	OCT
ICA		1.19 [0.84-1.72]	1.26 [0.65-2.39]
IVUS	0.84 [0.58-1.19]		1.06 [0.57-1.95]
OCT	0.80 [0.42-1.54]	0.94 [0.51-1.77]	
Regressor estimate [95% CrI]: 1.00 [0.84–1.16]			
**Ischemia-Driven Target Lesion Revascularization**			
	ICA	IVUS	OCT
ICA		1.54 [1.79-2.08]	1.39 [0.79-2.34]
IVUS	0.65 [0.48-0.90]		0.91 [0.54-1.57]
OCT	0.72 [0.43-1.27]	1.11 [0.64-1.86]	
Regressor estimate [95% CrI]: 1.04 [0.91–1.20]			
**Target Vessel Revascularization**			
	ICA	IVUS	OCT
ICA		1.58 [1.20-2.10]	1.25 [0.73-2.08]
IVUS	0.63 [0.47-0.84]		0.77 [0.47-1.23]
OCT	0.80 [0.48-1.38]	1.30 [0.81-2.12]	
Regressor estimate [95% CrI]: 1.03 [0.90–1.17]			
**Definite or Probable Stent Thrombosis**			
	ICA	IVUS	OCT
ICA		2.54 [1.09-6.30]	6.05 [0.91-46.99]
IVUS	0.40 [0.16-0.91]		2.32 [0.34-15.03]
OCT	0.17 [0.02-1.11]	0.43 [0.07-2.97]	
Regressor estimate [95% CrI]: 1.30 [0.83–2.10]			
**Major Adverse Cardiac Events**			
	ICA	IVUS	OCT
ICA		1.55 [1.23-1.97]	1.39 [0.91-2.08]

IVUS	0.64 [0.51-0.81]		0.90 [0.61-1.31]
OCT	0.72 [0.48-1.09]	1.12 [0.76-1.63]	
Regressor estimate [95% CrI]: 1.02 [0.91–1.14]			

ICA=Invasive Coronary Angiography; IVUS=Intravascular Ultrasound; OCT=Optical Coherence Tomography.

Values are OR [95% CrIs].

Online Table 40. Bayesian Random-Effects Network Meta-Regression Analysis by Bifurcation Disease.

**Target Lesion Revascularization**			
	ICA	IVUS	OCT
ICA		1.59 [1.18-2.27]	1.33 [0.92-2.13]
IVUS	0.63 [0.44-0.85]		0.83 [0.54-1.28]
OCT	0.75 [0.47-1.09]	1.21 [0.78-1.84]	
Regressor estimate [95% CrI]: 1.00 [0.87–1.15]			
**Myocardial Infarction**			
	ICA	IVUS	OCT
ICA		1.16 [0.80-1.70]	1.21 [0.83-1.82]
IVUS	0.86 [0.59-1.25]		1.01 [0.66-1.54]
OCT	0.83 [0.55-1.20]	0.85 [0.54-1.38]	
Regressor estimate [95% CrI]: 1.03 [0.93–1.15]			
**Death**			
	ICA	IVUS	OCT
ICA		1.47 [0.84-2.77]	1.45 [0.75-2.63]
IVUS	0.68 [0.36-1.19]		0.97 [0.44-1.79]
OCT	0.69 [0.38-1.34]	1.03 [0.56-2.25]	
Regressor estimate [95% CrI]: 1.04 [0.84–1.34]			
**Cardiac Death**			
	ICA	IVUS	OCT
ICA		1.79 [0.83-4.00]	2.04 [0.97-4.76]
IVUS	0.56 [0.25-1.21]		1.18 [0.49-2.78]
OCT	0.49 [0.21-1.03]	0.85 [0.36-2.03]	
Regressor estimate [95% CrI]: 1.16 [0.85–1.60]			
**Target Vessel Myocardial Infarction**			
	ICA	IVUS	OCT
ICA		0.95 [0.63-1.49]	1.19 [0.79-1.82]
IVUS	1.05 [0.67-1.60]		1.25 [0.77-2.00]
OCT	0.84 [0.55-1.26]	0.80 [0.50-1.30]	
Regressor estimate [95% CrI]: 1.12 [0.95–1.32]			
**Ischemia-Driven Target Lesion Revascularization**			
	ICA	IVUS	OCT
ICA		1.59 [1.19-2.17]	1.32 [0.90-2.00]
IVUS	0.63 [0.46-0.84]		0.83 [0.55-1.27]
OCT	0.76 [0.50-1.11]	1.20 [0.79-1.82]	
Regressor estimate [95% CrI]: 0.99 [0.89–1.11]			
**Target Vessel Revascularization**			
	ICA	IVUS	OCT
ICA		1.67 [1.25-2.22]	1.25 [0.86-1.86]
IVUS	0.60 [0.45-0.80]		0.73 [0.51-1.06]
OCT	0.80 [0.54-1.16]	1.37 [0.94-1.96]	
Regressor estimate [95% CrI]: 1.00 [0.87–1.14]			
**Definite or Probable Stent Thrombosis**			
	ICA	IVUS	OCT
ICA		2.13 [0.83-5.26]	2.38 [0.86-7.69]
IVUS	0.47 [0.19-1.21]		1.14 [0.35-4.00]
OCT	0.42 [0.13-1.17]	0.88 [0.25-2.83]	
Regressor estimate [95% CrI]: 1.19 [0.84–1.67]			
**Major Adverse Cardiac Events**			
	ICA	IVUS	OCT
ICA		1.56 [1.19-2.04]	1.41 [1.04-1.96]

IVUS	0.64 [0.49-0.84]		0.89 [0.66-1.24]
OCT	0.71 [0.51-0.96]	1.12 [0.81-1.51]	
Regressor estimate [95% CrI]: 1.04 [0.93–1.16]			

ICA=Invasive Coronary Angiography; IVUS=Intravascular Ultrasound; OCT=Optical Coherence Tomography.

Values are OR [95% CrIs].

Online Table 41. Bayesian Random-Effects Network Meta-Regression Analysis by Chronic Total Occlusion.

**Target Lesion Revascularization**			
	ICA	IVUS	OCT
ICA		1.54 [1.16-2.04]	1.35 [0.94-2.00]
IVUS	0.65 [0.49-0.86]		0.88 [0.60-1.37]
OCT	0.74 [0.50-1.06]	1.14 [0.73-1.68]	
Regressor estimate [95% CrI]: 0.95 [0.82–1.11]			
**Myocardial Infarction**			
	ICA	IVUS	OCT
ICA		1.10 [0.81-1.52]	1.18 [0.83-1.70]
IVUS	0.91 [0.66-1.23]		1.06 [0.70-1.59]
OCT	0.85 [0.59-1.20]	0.94 [0.63-1.43]	
Regressor estimate [95% CrI]: 1.03 [0.87–1.23]			
**Death**			
	ICA	IVUS	OCT
ICA		1.49 [1.00-2.33]	1.52 [0.94-2.38]
IVUS	0.67 [0.43-1.00]		1.02 [0.58-1.72]
OCT	0.66 [0.42-1.07]	0.98 [0.58-1.73]	
Regressor estimate [95% CrI]: 1.17 [0.81–1.72]			
**Cardiac Death**			
	ICA	IVUS	OCT
ICA		2.12 [1.25-3.90]	2.18 [1.16-4.26]
IVUS	0.47 [0.26-0.80]		1.04 [0.46-2.23]
OCT	0.46 [0.24-0.86]	0.96 [0.45-2.16]	
Regressor estimate [95% CrI]: 1.23 [0.77–1.97]			
**Target Vessel Myocardial Infarction**			
	ICA	IVUS	OCT
ICA		1.12 [0.82-1.54]	1.24 [0.87-1.75]
IVUS	0.90 [0.65-1.22]		1.14 [0.73-1.77]
OCT	0.81 [0.57-1.15]	0.88 [0.57-1.38]	
Regressor estimate [95% CrI]: 1.03 [0.85–1.25]			
**Ischemia-Driven Target Lesion Revascularization**			
	ICA	IVUS	OCT
ICA		1.54 [1.15-2.05]	1.35 [0.94-2.01]
IVUS	0.65 [0.49-0.87]		0.88 [0.60-1.34]
OCT	0.74 [0.50-1.06]	1.14 [0.75-1.68]	
Regressor estimate [95% CrI]: 0.95 [0.80–1.12]			
**Target Vessel Revascularization**			
	ICA	IVUS	OCT
ICA		1.62 [1.26-2.10]	1.22 [0.89-1.67]
IVUS	0.62 [0.48-0.80]		0.76 [0.53-1.09]
OCT	0.82 [0.60-1.13]	1.32 [0.91-1.90]	
Regressor estimate [95% CrI]: 0.99 [0.85–1.15]			
**Definite or Probable Stent Thrombosis**			
	ICA	IVUS	OCT
ICA		2.05 [0.92-4.76]	2.64 [0.91-8.94]
IVUS	0.49 [0.21-1.88]		1.28 [0.38-5.21]
OCT	0.38 [0.11-1.08]	0.78 [0.19-2.67]	
Regressor estimate [95% CrI]: 0.98 [0.44–1.88]			
**Major Adverse Cardiac Events**			
	ICA	IVUS	OCT
ICA		1.55 [1.26-1.94]	1.43 [1.08-1.95]

IVUS	0.64 [0.52-0.80]		0.93 [0.68-1.28]
OCT	0.70 [0.51-0.92]	1.07 [0.78-1.48]	
Regressor estimate [95% CrI]: 0.99 [0.92–1.06]			

ICA=Invasive Coronary Angiography; IVUS=Intravascular Ultrasound; OCT=Optical Coherence Tomography.

Values are OR [95% CrIs].

Online Table 42. Frequentist Random-Effects Network Meta-Analysis in nonEast Asian Trials.

**Target Lesion Revascularization**			
	ICA	IVUS	OCT
ICA		1.05 [0.58–1.90]	1.08 [0.76–1.54]
IVUS	0.95 [0.53–1.71]		1.03 [0.53–2.00]
OCT	0.92 [0.65–1.31]	0.97 [0.50–1.89]	
Heterogeneity: I^2=0%; τ^2=0.013			
**Myocardial Infarction**			
	ICA	IVUS	OCT
ICA		1.41 [0.72–2.75]	1.11 [0.86–1.43]
IVUS	0.71 [0.36–1.38]		0.78 [0.39–1.58]
OCT	0.90 [0.70–1.17]	1.28 [0.63–2.56]	
Heterogeneity: I^2=0%; τ^2=0			
**Death**			
	ICA	IVUS	OCT
ICA		1.57 [0.39–6.21]	1.39 [0.96–2.01]
IVUS	0.64 [0.16–2.53]		0.89 [0.22–3.64]
OCT	0.72 [0.50–1.04]	1.13 [0.27–4.64]	
Heterogeneity: I^2=0%; τ^2=0			
**Cardiac Death**			
	ICA	IVUS	OCT
ICA		1.46 [0.37–5.82]	1.60 [0.92–2.77]
IVUS	0.69 [0.17–2.74]		1.10 [0.25–4.75]
OCT	0.63 [0.36–1.08]	0.91 [0.21–3.96]	
Heterogeneity: I^2=0%; τ^2=0			
**Target Vessel Myocardial Infarction**			
	ICA	IVUS	OCT
ICA		1.10 [0.84–1.44]	1.22 [0.92–1.62]
IVUS	0.91 [0.69–1.19]		1.11 [0.78–1.58]
OCT	0.82 [0.62–1.09]	0.90 [0.63–1.29]	
Heterogeneity: I^2=0%; τ^2=0			
**Ischemia-Driven Target Lesion Revascularization**			
	ICA	IVUS	OCT
ICA		1.05 [0.58–1.91]	1.09 [0.75–1.57]
IVUS	0.95 [0.52–1.73]		1.04 [0.53–2.04]
OCT	0.92 [0.64–1.33]	0.96 [0.49–1.90]	
Heterogeneity: I^2=0%; τ^2=0.018			
**Target Vessel Revascularization**			
	ICA	IVUS	OCT
ICA		1.38 [0.79–2.40]	1.07 [0.79–1.44]
IVUS	0.73 [0.42–1.27]		0.77 [0.42–1.43]
OCT	0.94 [0.70–1.26]	1.29 [0.70–2.38]	
Heterogeneity: I^2=0%; τ^2=0.003			
**Definite or Probable Stent Thrombosis**			
	ICA	IVUS	OCT
ICA		1.32 [0.43–4.07]	1.78 [0.90–3.53]
IVUS	0.76 [0.25–2.34]		1.35 [0.37–4.90]
OCT	0.56 [0.28–1.11]	0.74 [0.20–2.68]	
Heterogeneity: I^2=0%; τ^2=0			
**Major Adverse Cardiac Events**			
	ICA	IVUS	OCT
ICA		1.24 [0.80–1.92]	1.21 [0.97–1.52]

IVUS	0.81 [0.52–1.25]		1.00 [0.60–1.58]
OCT	0.83 [0.66–1.04]	1.02 [0.63–1.66]	
Heterogeneity: I^2=0%; τ^2=0.			

ICA=Invasive Coronary Angiography; IVUS=Intravascular Ultrasound; OCT=Optical Coherence Tomography.

Values are Odds Ratio [95% CIs].

Online Table 43. Bayesian Random-Effects Network Meta-Analysis in nonEast Asian Trials.

**Target Lesion Revascularization**			
	ICA	IVUS	OCT
ICA		1.04 [0.41–2.40]	1.03 [0.43–1.48]
IVUS	0.96 [0.42–2.41]		0.98 [0.32–2.66]
OCT	0.97 [0.54–2.32]	1.02 [0.38–3.11]	
Heterogeneity: I^2=0%; τ^2=0			
**Myocardial Infarction**			
	ICA	IVUS	OCT
ICA		1.49 [0.67–3.57]	1.06 [0.55–1.68]
IVUS	0.67 [0.28–1.48]		0.70 [0.25–1.62]
OCT	0.94 [0.60–1.82]	1.42 [0.62–4.05]	
Heterogeneity: I^2=0%; τ^2=0			
**Death**			
	ICA	IVUS	OCT
ICA		3.65 [0.38–646.00]	0.93 [0.20–17.02]
IVUS	0.27 [0.00–2.61]		0.28 [0.00–3.03]
OCT	0.93 [0.20–17.02]	3.63 [0.33–2092.00]	
Heterogeneity: I^2=0%; τ^2=0			
**Cardiac Death**			
	ICA	IVUS	OCT
ICA		3.34 [0.34–741.7]	1.28 [0.07–6.79]
IVUS	0.30 [0.00–2.98]		0.37 [0.00–4.47]
OCT	0.78 [0.15–14.44]	2.68 [0.22–2189.00]	
Heterogeneity: I^2=0%; τ^2=0			
**Target Vessel Myocardial Infarction**			
	ICA	IVUS	OCT
ICA		1.59 [0.64–4.18]	1.12 [0.53–1.91]
IVUS	0.63 [0.24–1.56]		0.70 [0.21–1.87]
OCT	0.89 [0.52–1.88]	1.42 [0.54–4.76]	
Heterogeneity: I^2=0%; τ^2=0			
**Ischemia-Driven Target Lesion Revascularization**			
	ICA	IVUS	OCT
ICA		1.03 [0.40–2.44]	1.03 [0.42–1.86]
IVUS	0.97 [0.41–2.52]		1.00 [0.32–2.73]
OCT	0.97 [0.54–2.38]	1.00 [0.37–3.17]	
Heterogeneity: I^2=0%; τ^2=0.018			
**Target Vessel Revascularization**			
	ICA	IVUS	OCT
ICA		1.35 [0.55–3.07]	0.98 [0.39–1.65]
IVUS	0.74 [0.33–3.07]		0.72 [0.22–1.78]
OCT	0.98 [0.39–1.65]	1.39 [0.56–4.52]	
Heterogeneity: I^2=0%; τ^2=0			
**Definite or Probable Stent Thrombosis**			
	ICA	IVUS	OCT
ICA		1.27 [0.06–19.85]	1.52 [0.14–8.45]
IVUS	0.79 [0.05–16.91]		1.19 [0.04–8.45]
OCT	0.66 [0.12–7.08]	0.84 [0.03–27.01]	
Heterogeneity: I^2=0%; τ^2=0			
**Major Adverse Cardiac Events**			
	ICA	IVUS	OCT

ICA		1.19 [0.59–2.15]	1.15 [0.64–1.74]
IVUS	0.84 [0.46–1.69]		0.96 [0.45–1.99]
OCT	0.87 [0.57–1.57]	1.05 [0.50–2.22]	
Heterogeneity: I^2=0%; τ^2=0			

ICA=Invasive Coronary Angiography; IVUS=Intravascular Ultrasound; OCT=Optical Coherence Tomography.

Values are Odds Ratio [95% CrIs].

Online Table 44. Frequentist Random-Effects Network Meta-Analysis in East Asian Trials.

**Target Lesion Revascularization**			
	ICA	IVUS	OCT
ICA		1.66 [1.27–2.17]	1.74 [1.01–3.00]
IVUS	0.60 [0.46–0.79]		1.05 [0.64–1.73]
OCT	0.57 [0.33–0.99]	0.95 [0.58–1.57]	
Heterogeneity: I^2=0%; τ^2=0			
**Myocardial Infarction**			
	ICA	IVUS	OCT
ICA		1.10 [0.81–1.49]	1.59 [0.74–3.43]
IVUS	0.91 [0.67–1.24]		1.45 [0.71–2.95]
OCT	0.63 [0.29–1.35]	0.69 [0.34–1.40]	
Heterogeneity: I^2=0%; τ^2=0			
**Death**			
	ICA	IVUS	OCT
ICA		1.30 [0.90–1.88]	1.47 [0.72–3.02]
IVUS	0.77 [0.53–1.12]		1.14 [0.59–2.18]
OCT	0.68 [0.33–1.39]	0.88 [0.46–1.69]	
Heterogeneity: I^2=0%; τ^2=0			
**Cardiac Death**			
	ICA	IVUS	OCT
ICA		1.84 [1.13–3.01]	2.22 [0.83–5.94]
IVUS	0.54 [0.33–0.89]		1.20 [0.46–3.12]
OCT	0.45 [0.17–1.20]	0.83 [0.32–2.15]	
Heterogeneity: I^2=0%; τ^2=0			
**Target Vessel Myocardial Infarction**			
	ICA	IVUS	OCT
ICA		1.10 [0.81–1.50]	1.59 [0.74–3.43]
IVUS	0.91 [0.67–1.24]		1.45 [0.71–2.95]
OCT	0.63 [0.29–1.35]	0.69 [0.34–1.40]	
Heterogeneity: I^2=0%; τ^2=0			
**Ischemia-Driven Target Lesion Revascularization**			
	ICA	IVUS	OCT
ICA		1.66 [1.27–2.17]	1.74 [1.01–3.00]
IVUS	0.60 [0.46–0.79]		1.05 [0.64–1.73]
OCT	0.57 [0.33–0.99]	0.95 [0.58–1.57]	
Heterogeneity: I^2=0%; τ^2=0.018			
**Target Vessel Revascularization**			
	ICA	IVUS	OCT
ICA		1.64 [1.27–2.12]	1.43 [0.89–2.30]
IVUS	0.61 [0.47–0.79]		0.87 [0.57–1.33]
OCT	0.70 [0.43–1.13]	1.15 [0.75–1.76]	
Heterogeneity: I^2=0%; τ^2=0			
**Definite or Probable Stent Thrombosis**			
	ICA	IVUS	OCT
ICA		1.96 [1.01–3.78]	4.80 [0.88–26.28]
IVUS	0.51 [0.26–0.99]		2.46 [0.48–12.61]
OCT	0.21 [0.04–1.14]	0.41 [0.08–2.09]	
Heterogeneity: I^2=0%; τ^2=0			
**Major Adverse Cardiac Events**			
	ICA	IVUS	OCT
ICA		1.61 [1.30–2.00]	1.64 [1.07–2.52]

IVUS	0.62 [0.50–0.77]		1.02 [0.68–1.52]
OCT	0.61 [0.40–0.94]	0.98 [0.66–1.47]	
Heterogeneity: I^2=0%; τ^2=0.013			

ICA=Invasive Coronary Angiography; IVUS=Intravascular Ultrasound; OCT=Optical Coherence Tomography.

Values are Odds Ratio [95% CIs].

Online Table 45. Bayesian Random-Effects Network Meta-Analysis in East Asian Trials.

**Target Lesion Revascularization**			
	ICA	IVUS	OCT
ICA		1.68 [1.23–2.34]	1.78 [0.96–3.27]
IVUS	0.60 [0.43–0.81]		0.95 [0.54–1.68]
OCT	0.56 [0.31–1.04]	1.06 [0.60–1.84]	
Heterogeneity: I^2=0%; τ^2=0			
**Myocardial Infarction**			
	ICA	IVUS	OCT
ICA		1.16 [0.78–1.94]	1.70 [0.67–4.77]
IVUS	0.86 [0.76–1.94]		1.47 [0.61–3.59]
OCT	0.59 [0.21–1.50]	0.68 [0.28–1.65]	
Heterogeneity: I^2=0%; τ^2=0			
**Death**			
	ICA	IVUS	OCT
ICA		1.37 [0.87–2.26]	1.45 [0.61–3.40]
IVUS	0.73 [0.44-1.14]		1.06 [0.47–2.25]
OCT	0.69 [0.29–1.63]	0.94 [0.44–2.14]	
Heterogeneity: I^2=0%; τ^2=0			
**Cardiac Death**			
	ICA	IVUS	OCT
ICA		2.01 [1.12–4.06]	2.45 [0.73–9.01]
IVUS	0.50 [0.25–0.90]		1.22 [0.36–4.05]
OCT	0.41 [0.11–1.37]	0.82 [0.25–2.79]	
Heterogeneity: I^2=0%; τ^2=0			
**Target Vessel Myocardial Infarction**			
	ICA	IVUS	OCT
ICA		1.17 [0.78–1.99]	1.71 [0.66–5.06]
IVUS	0.86 [0.50–1.28]		1.45 [0.59–3.75]
OCT	0.59 [0.20–1.53]	0.69 [0.27–1.68]	
Heterogeneity: I^2=0%; τ^2=0			
**Ischemia-Driven Target Lesion Revascularization**			
	ICA	IVUS	OCT
ICA		1.69 [1.23–2.33]	1.78 [0.98–3.27]
IVUS	0.59 [0.43–0.81]		1.06 [0.60–1.86]
OCT	0.56 [0.31–1.02]	0.95 [0.54–1.67]	
Heterogeneity: I^2=0%; τ^2=0			
**Target Vessel Revascularization**			
	ICA	IVUS	OCT
ICA		1.68 [1.25–2.30]	1.43 [0.82–2.49]
IVUS	0.59 [0.43–0.80]		0.85 [0.51–1.40]
OCT	0.70 [0.40–1.22]	1.18 [0.71–1.98]	
Heterogeneity: I^2=0%; τ^2=0			
**Definite or Probable Stent Thrombosis**			
	ICA	IVUS	OCT
ICA		2.61 [1.62–7.62]	19.34 [1.62–1000.00]
IVUS	0.38 [0.13–0.90]		7.26 [0.68–327.60]
OCT	0.05 [0.00–0.62]	0.14 [0.00–1.46]	
Heterogeneity: I^2=0%; τ^2=0			
**Major Adverse Cardiac Events**			
	ICA	IVUS	OCT
ICA		1.64 [1.27–2.17]	1.64 [0.99–2.72]

IVUS	0.61 [0.46–0.79]		1.00 [0.62–1.59]
OCT	0.61 [0.37–1.01]	1.00 [0.63–1.62]	
Heterogeneity: I^2=0%; τ^2=0.013			

ICA=Invasive Coronary Angiography; IVUS=Intravascular Ultrasound; OCT=Optical Coherence Tomography.

Values are Odds Ratio [95% CrIs].

Online Table 46. Frequentist and Bayesian Random-Effects Pairwise Meta-Analysis of Trials Comparing IVI- vs ICA-guided PCI by IRR.

Frequentist Random-Effects Model	IRR [95% CI]	P	I^2 (%)
Target Lesion Revascularization	0.66 [0.54–0.80]	<0.001	0
Myocardial Infarction	0.85 [0.72–1.01]	0.056	0
Death	0.74 [0.59–0.92]	0.007	0
Cardiac Death	0.55 [0.40–0.75]	<0.001	0
Target Vessel Myocardial Infarction	0.82 [0.68–0.99]	0.035	0
Ischemia-Driven Target Lesion Revascularization	0.66 [0.53–0.81]	<0.001	0
Target Vessel Revascularization	0.66 [0.54–0.80]	<0.001	0
Definite or Probable Stent Thrombosis	0.52 [0.34–0.80]	0.003	0
Major Adverse Cardiac Events	0.66 [0.55–0.80]	<0.001	0
**Bayesian Random-Effects Model**	**IRR [95% CrI]**		**I^2 (%)**
Target Lesion Revascularization	0.70 [0.58–0.85]		0
Myocardial Infarction	0.85 [0.69–1.04]		0
Death	0.73 [0.56–0.95]		0
Cardiac Death	0.55 [0.38–0.80]		0
Target Vessel Myocardial Infarction	0.82 [0.65–1.02]		0
Ischemia-Driven Target Lesion Revascularization	0.72 [0.58–0.89]		0
Target Vessel Revascularization	0.72 [0.58–0.88]		0
Definite or Probable Stent Thrombosis	0.47 [0.26–0.80]		0
Major Adverse Cardiac Events	0.71 [0.61–0.83]		3

CI=Confidence Interval; CrI=Credible Interval; OR=Odds Ratio.

Online Table 47. Frequentist and Bayesian Random-Effects Pairwise Meta-Analysis of Trials Comparing IVI- vs ICA-guided PCI by Definition.

Frequentist Random-Effects Model	OR [95% CI]	P	I^2 (%)
**Target Lesion Revascularization**	0.71 [0.58–0.88]	0.002	0
**Myocardial Infarction**	0.86 [0.70–1.05]	0.128	0
**Death**	0.77 [0.61–0.98]	0.035	0
**Cardiac Death**	0.54 [0.39–0.74]	<0.001	0
**Target Vessel Myocardial Infarction**	0.80 [0.64–0.99]	0.043	0
**Ischemia-Driven Target Lesion Revascularization**	0.74 [0.56–0.97]	0.028	20
**Target Vessel Revascularization**	0.72 [0.58–0.88]	0.002	0
**Definite or Probable Stent Thrombosis**	0.53 [0.34–0.84]	0.006	0
**Major Adverse Cardiac Events**	0.71 [0.58–0.88]	0.001	25
**Bayesian Random-Effects Model**	**OR [95% CrI]**		**I^2 (%)**
**Target Lesion Revascularization**	0.65 [0.51–0.85]		0
**Myocardial Infarction**	0.86 [0.67–1.08]		0
**Death**	0.77 [0.58–1.06]		0
**Cardiac Death**	0.52 [0.36–0.74]		0
**Target Vessel Myocardial Infarction**	0.79 [0.58–1.07]		0
**Ischemia-Driven Target Lesion Revascularization**	0.75 [0.53–1.11]		0
**Target Vessel Revascularization**	0.64 [0.49–0.82]		0
**Definite or Probable Stent Thrombosis**	0.46 [0.25–0.79]		0
**Major Adverse Cardiac Events**	0.72 [0.54–0.95]		15

CI=Confidence Interval; CrI=Credible Interval; OR=Odds Ratio.

Online Table 48. Frequentist and Bayesian Random-Effects Pairwise Meta-Analysis of Trials Comparing IVI- vs ICA-guided PCI at the Longest Available Follow-Up.

Frequentist Random-Effects Model	OR [95% CI]	P	I^2 (%)
**Target Lesion Revascularization**	0.70 [0.58–0.86]	0.001	0
**Myocardial Infarction**	0.83 [0.70–1.00]	0.045	0
**Death**	0.74 [0.59–0.92]	0.008	0
**Cardiac Death**	0.55 [0.41–0.74]	<0.001	0
**Target Vessel Myocardial Infarction**	0.80 [0.66–0.97]	0.023	0
**Ischemia-Driven Target Lesion Revascularization**	0.70 [0.57–0.86]	0.001	0
**Target Vessel Revascularization**	0.70 [0.58–0.84]	<0.001	0
**Definite or Probable Stent Thrombosis**	0.52 [0.34–0.80]	0.003	0
**Major Adverse Cardiac Events**	0.68 [0.59–0.79]	<0.001	11
**Bayesian Random-Effects Model**	**OR [95% CrI]**		**I^2 (%)**
**Target Lesion Revascularization**	0.64 [0.51–0.81]		0
**Myocardial Infarction**	0.82 [0.65–1.02]		0
**Death**	0.72 [0.55–0.94]		0
**Cardiac Death**	0.53 [0.37–0.74]		0
**Target Vessel Myocardial Infarction**	0.79 [0.62–0.99]		0
**Ischemia-Driven Target Lesion Revascularization**	0.64 [0.51–0.81]		0
**Target Vessel Revascularization**	0.64 [0.52–0.80]		0
**Definite or Probable Stent Thrombosis**	0.45 [0.25–0.75]		0
**Major Adverse Cardiac Events**	0.68 [0.58–0.81]		8

CI=Confidence Interval; CrI=Credible Interval; OR=Odds Ratio.

Online Table 49. Frequentist and Bayesian Random-Effects Pairwise Meta-Analysis of Trials Comparing IVI- vs ICA-guided PCI After Excluding Trials with Higher Risk of Bias.

Frequentist Random-Effects Model	OR [95% CI]	P	I^2 (%)
Target Lesion Revascularization	0.73 [0.59–0.91]	0.043	0
Myocardial Infarction	0.86 [0.72–1.03]	0.104	0
Death	0.74 [0.58–0.93]	0.010	0
Cardiac Death	0.53 [0.38–0.74]	<0.001	0
Target Vessel Myocardial Infarction	0.83 [0.68–1.01]	0.062	0
Ischemia-Driven Target Lesion Revascularization	0.72 [0.58–0.90]	0.043	0
Target Vessel Revascularization	0.73 [0.60–0.90]	0.025	0
Definite or Probable Stent Thrombosis	0.49 [0.30–0.79]	0.004	0
Major Adverse Cardiac Events	0.70 [0.60–0.82]	<0.001	17
**Bayesian Random-Effects Model**	**OR [95% CrI]**		**I^2 (%)**
Target Lesion Revascularization	0.73 [0.57–0.94]		0
Myocardial Infarction	0.86 [0.68–1.08]		0
Death	0.73 [0.55–0.96]		0
Cardiac Death	0.52 [0.35–0.75]		0
Target Vessel Myocardial Infarction	0.83 [0.64–1.06]		0
Ischemia-Driven Target Lesion Revascularization	0.72 [0.56–0.94]		0
Target Vessel Revascularization	0.73 [0.58–0.93]		0
Definite or Probable Stent Thrombosis	0.40 [0.18–0.75]		0
Major Adverse Cardiac Events	0.70 [0.58–0.85]		12

CI=Confidence Interval; CrI=Credible Interval; OR=Odds Ratio.

Online Table 50. Frequentist and Bayesian Random-Effects Pairwise Meta-Analysis of Trials Comparing IVI- vs ICA-guided PCI After Excluding Trials Without Primary Clinical Endpoints to be Assessed at Mid- or Long-Term Follow-Up.

Frequentist Random-Effects Model	OR [95% CI]	P	I^2 (%)
Target Lesion Revascularization	0.72 [0.59–0.89]	0.001	0
Myocardial Infarction	0.82 [0.67–1.02]	0.069	0
Death	0.74 [0.58–0.94]	0.012	0
Cardiac Death	0.54 [0.37–0.79]	<0.001	0
Target Vessel Myocardial Infarction	0.79 [0.63–0.99]	0.044	0
Ischemia-Driven Target Lesion Revascularization	0.70 [0.55–0.88]	0.003	0
Target Vessel Revascularization	0.71 [0.57–0.88]	0.002	3
Definite or Probable Stent Thrombosis	0.53 [0.32–0.89]	0.002	0
Major Adverse Cardiac Events	0.67 [0.57–0.79]	<0.001	18
**Bayesian Random-Effects Model**	**OR [95% CrI]**		**I^2 (%)**
Target Lesion Revascularization	0.70 [0.52–0.91]		1
Myocardial Infarction	0.81 [0.61–1.04]		15
Death	0.73 [0.54–0.99]		0
Cardiac Death	0.53 [0.35–0.80]		0
Target Vessel Myocardial Infarction	0.78 [0.58–1.01]		14
Ischemia-Driven Target Lesion Revascularization	0.69 [0.52–0.91]		2
Target Vessel Revascularization	0.70 [0.53–0.90]		5
Definite or Probable Stent Thrombosis	0.48 [0.26–0.82]		0
Major Adverse Cardiac Events	0.67 [0.54–0.81]		9

CI=Confidence Interval; CrI=Credible Interval; OR=Odds Ratio.

Online Table 51. Meta-Regression Analysis of Trials Comparing IVI- vs ICA-guided PCI.

Diabetes	OR [95% CrI]	Regressor Estimate [95% CrI]	I^2 (%)
Target Lesion Revascularization	0.69 [0.57–0.83]	1.04 [0.95–1.13]	0
Myocardial Infarction	0.85 [0.68–1.05]	1.03 [0.93–1.05]	0
Death	0.71 [0.54–0.95]	0.97 [0.87–1.05]	0
Cardiac Death	0.53 [0.37–0.76]	0.99 [0.90–1.11]	0
Target Vessel Myocardial Infarction	0.83 [0.65–1.04]	1.03 [0.95–1.11]	0
Ischemia-Driven Target Lesion Revascularization	0.71 [0.57–0.90]	1.00 [0.92–1.07]	0
Target Vessel Revascularization	0.71 [0.57–0.88]	0.99 [0.92–1.06]	0
Definite or Probable Stent Thrombosis	0.44 [0.25–0.77]	0.97 [0.83–1.14]	0
Major Adverse Cardiac Events	0.69 [0.58–0.82]	1.00 [0.95–1.05]	0
**Acute Coronary Syndrome**	**OR [95% CrI]**	**Regressor Estimate [95% CrI]**	**I^2 (%)**
Target Lesion Revascularization	0.70 [0.55–0.89]	1.04 [0.91–1.16]	0
Myocardial Infarction	0.82 [0.65–1.04]	1.02 [0.92–1.13]	0
Death	0.71 [0.54–0.96]	1.01 [0.89–1.15]	0
Cardiac Death	0.52 [0.35–0.75]	1.04 [0.88–1.23]	0
Target Vessel Myocardial Infarction	0.80 [0.61–1.03]	1.03 [0.91–1.17]	0
Ischemia-Driven Target Lesion Revascularization	0.68 [0.54–0.88]	1.05 [0.94–1.16]	0
Target Vessel Revascularization	0.70 [0.55–0.87]	1.02 [0.91–1.13]	0
Definite or Probable Stent Thrombosis	0.46 [0.24–0.81]	0.96 [0.73–1.30]	0
Major Adverse Cardiac Events	0.68 [0.57–0.80]	1.04 [0.96–1.13]	0
**Stent Length**	**OR [95% CrI]**	**Regressor Estimate [95% CrI]**	**I^2 (%)**
Target Lesion Revascularization	0.64 [0.49–0.84]	1.01 [0.95–1.19]	0
Myocardial Infarction	0.81 [0.61–1.11]	1.01 [0.90–1.28]	0
Death	0.75 [0.50–1.11]	0.97 [0.83–1.14]	0
Cardiac Death	0.50 [0.31–0.80]	1.02 [0.84–1.22]	0
Target Vessel Myocardial Infarction	0.81 [0.58–1.11]	0.99 [0.88–1.11]	0
Ischemia-Driven Target Lesion Revascularization	0.64 [0.48–0.84]	1.07 [0.95–1.20]	0
Target Vessel Revascularization	0.70 [0.55–0.87]	1.07 [0.96–1.21]	0
Definite or Probable Stent Thrombosis	0.36 [0.16–0.76]	1.11 [0.82–1.51]	0
Major Adverse Cardiac Events	0.63 [0.52–0.78]	1.04 [0.96–1.14]	0
**Chronic Total Occlusion**	**OR [95% CrI]**	**Regressor Estimate [95% CrI]**	**I^2 (%)**
Target Lesion Revascularization	0.64 [0.45–0.86]	0.98 [0.84–1.13]	0
Myocardial Infarction	0.91 [0.66–1.30]	1.09 [0.93–1.31]	0
Death	0.69 [0.42–1.11]	1.02 [0.80–1.36]	0
Cardiac Death	0.50 [0.25–0.91]	1.07 [0.75–1.51]	0
Target Vessel Myocardial Infarction	0.87 [0.62–1.25]	1.07 [0.91–1.28]	0
Ischemia-Driven Target Lesion Revascularization	0.64 [0.46–0.85]	0.98 [0.84–1.14]	0
Target Vessel Revascularization	0.66 [0.47–0.87]	1.01 [0.87–1.17]	0
Definite or Probable Stent Thrombosis	0.39 [0.18–0.80]	1.11 [0.80–1.55]	0
Major Adverse Cardiac Events	0.65 [0.51–0.81]	1.02 [0.90–1.14]	0
**Chronic Total Occlusion**	**OR [95% CrI]**	**Regressor Estimate [95% CrI]**	**I^2 (%)**
Target Lesion Revascularization	0.66 [0.51–0.83]	1.05 [0.93–1.16]	0
Myocardial Infarction	0.84 [0.66–1.06]	0.99 [0.85–1.16]	0
Death	0.66 [0.46–0.90]	1.13 [0.87–1.23]	0
Cardiac Death	0.45 [0.29–0.69]	1.12 [0.80–1.60]	0
Target Vessel Myocardial Infarction	0.81 [0.64–1.03]	0.99 [0.85–1.16]	0
Ischemia-Driven Target Lesion Revascularization	0.67 [0.52–0.85]	0.95 [0.81–1.13]	0

**Target Vessel Revascularization**	0.67 [0.52–0.84]	0.99 [0.87–1.15]	0
**Definite or Probable Stent Thrombosis**	0.43 [0.21–0.81]	0.89 [0.47–1.57]	0
**Major Adverse Cardiac Events**	0.66 [0.54–0.78]	0.99 [0.88–1.11]	0

CrI=Credible Interval; OR=Odds Ratio.

Online Table 52. Frequentist and Bayesian Random-Effects Network Meta-Analysis of Trials comparing IVI- vs ICA-guided PCI in nonEast Asian Trials.

Frequentist Random-Effects Model	OR [95% CI]	P	I^2 (%)
Target Lesion Revascularization	0.85 [0.64–1.13]	0.268	0
Myocardial Infarction	0.83 [0.67–1.03]	0.084	0
Death	0.72 [0.54–0.96]	0.027	0
Cardiac Death	0.56 [0.37–0.85]	0.006	0
Target Vessel Myocardial Infarction	0.77 [0.60–0.99]	0.038	0
Ischemia-Driven Target Lesion Revascularization	0.85 [0.64–1.13]	0.267	0
Target Vessel Revascularization	0.83 [0.64–1.07]	0.151	0
Definite or Probable Stent Thrombosis	0.54 [0.31–0.97]	0.040	0
Major Adverse Cardiac Events	0.76 [0.63–0.91]	0.003	0
**Bayesian Random-Effects Model**	**OR [95% CrI]**		**I^2 (%)**
Target Lesion Revascularization	0.87 [0.60–1.42]		0
Myocardial Infarction	0.83 [0.59–1.18]		0
Death	0.72 [0.42–1.34]		0
Cardiac Death	0.57 [0.29–1.28]		0
Target Vessel Myocardial Infarction	0.77 [0.54–1.15]		0
Ischemia-Driven Target Lesion Revascularization	0.87 [0.59–1.41]		0
Target Vessel Revascularization	0.85 [0.59–1.35]		0
Definite or Probable Stent Thrombosis	0.54 [0.19–1.92]		0
Major Adverse Cardiac Events	0.79 [0.60–1.16]		12

CI=Confidence Interval; CrI=Credible Interval; OR=Odds Ratio.

Online Table 53. Frequentist and Bayesian Random-Effects Network Meta-Analysis of Trials comparing IVI- vs ICA-guided PCI in East Asian Trials.

Frequentist Random-Effects Model	OR [95% CI]	P	I^2 (%)
Target Lesion Revascularization	0.72 [0.59–0.88]	0.001	0
Myocardial Infarction	0.91 [0.67–1.23]	0.534	0
Death	0.76 [0.53–1.11]	0.154	0
Cardiac Death	0.53 [0.33–0.86]	0.010	0
Target Vessel Myocardial Infarction	0.91 [0.67–1.23]	0.532	0
Ischemia-Driven Target Lesion Revascularization	0.60 [0.46–0.78]	<0.001	0
Target Vessel Revascularization	0.61 [0.47–0.79]	<0.001	0
Definite or Probable Stent Thrombosis	0.49 [0.26–0.95]	0.034	0
Major Adverse Cardiac Events	0.62 [0.50–0.89]	<0.001	4
**Bayesian Random-Effects Model**	**OR [95% CrI]**		**I^2 (%)**
Target Lesion Revascularization	0.59 [0.42–0.82]		0
Myocardial Infarction	0.85 [0.45–1.29]		4
Death	0.73 [0.44–1.15]		0
Cardiac Death	0.49 [0.25–0.89]		0
Target Vessel Myocardial Infarction	0.85 [0.46–1.29]		4
Ischemia-Driven Target Lesion Revascularization	0.59 [0.43–0.81]		0
Target Vessel Revascularization	0.60 [0.43–0.82]		0
Definite or Probable Stent Thrombosis	0.37 [0.12–0.86]		0
Major Adverse Cardiac Events	0.61 [0.46–0.79]		0

CI=Confidence Interval; CrI=Credible Interval; OR=Odds Ratio.

Online Table 54. Assessment of the Results According to GRADE.

Qualitative Assessment						
**Trials**	**Study Design**	**Risk of Bias**	**Inconsistency**	**Indirectness**	**Imprecision**	**Overall Quality**
**Target Lesion Revascularization (IVUS vs ICA)**						
14	Randomized	Moderate	Not serious	Not serious	Not serious	⊕⊕⊕ Moderate
**Target Lesion Revascularization (OCT vs ICA)**						
9	Randomized	Not serious	Serious	Not serious	Not serious	⊕⊕ Serious
**Target Lesion Revascularization (OCT vs IVUS)**						
5	Randomized	Not serious	Moderate	Not serious	Not serious	⊕⊕⊕ Moderate
**Myocardial Infarction (IVUS vs ICA)**						
14	Randomized	Moderate	Not serious	Not serious	Not serious	⊕⊕⊕ Moderate
**Myocardial Infarction (OCT vs ICA)**						
9	Randomized	Not serious	Not serious	Not serious	Not serious	⊕⊕⊕⊕ High
**Myocardial Infarction (OCT vs IVUS)**						
5	Randomized	Not serious	Not serious	Not serious	Not serious	⊕⊕⊕⊕ High
**Death (IVUS vs ICA)**						
14	Randomized	Moderate	Not serious	Not serious	Not serious	⊕⊕⊕ Moderate
**Death (OCT vs ICA)**						
9	Randomized	Not serious	Not serious	Not serious	Not serious	⊕⊕⊕⊕ High
**Death (OCT vs IVUS)**						
5	Randomized	Not serious	Not serious	Not serious	Not serious	⊕⊕⊕⊕ High
**Cardiac Death (IVUS vs ICA)**						
14	Randomized	Moderate	Not serious	Not serious	Not serious	⊕⊕⊕ Moderate
**Cardiac Death (OCT vs ICA)**						
9	Randomized	Not serious	Not serious	Not serious	Not serious	⊕⊕⊕⊕ High
**Cardiac Death (OCT vs IVUS)**						
5	Randomized	Not serious	Not serious	Not serious	Not serious	⊕⊕⊕⊕ High
**Target-Vessel Myocardial Infarction (IVUS vs ICA)**						
14	Randomized	Moderate	Not serious	Not serious	Moderate	⊕⊕ Serious
**Target-Vessel Myocardial Infarction (OCT vs ICA)**						
9	Randomized	Not serious	Not serious	Not serious	Not serious	⊕⊕⊕⊕ High
**Target-Vessel Myocardial Infarction (OCT vs IVUS)**						
5	Randomized	Not serious	Not serious	Not serious	Not serious	⊕⊕⊕⊕ High
**Ischemia-Driven Target Lesion Revascularization (IVUS vs ICA)**						

14	Randomized	Moderate	Not serious	Not serious	Moderate	⊕⊕<br>Serious
**Ischemia-Driven Target Lesion Revascularization (OCT vs ICA)**						
9	Randomized	Not serious	Serious	Not serious	Not serious	⊕⊕<br>Serious
**Ischemia-Driven Target Lesion Revascularization (OCT vs IVUS)**						
5	Randomized	Not serious	Moderate	Not serious	Not serious	⊕⊕⊕<br>Moderate
**Target-Vessel Revascularization (IVUS vs ICA)**						
14	Randomized	Moderate	Not serious	Not serious	Moderate	⊕⊕<br>Serious
**Target-Vessel Revascularization (OCT vs ICA)**						
9	Randomized	Not serious	Not serious	Not serious	Moderate	⊕⊕⊕<br>Moderate
**Target-Vessel Revascularization (OCT vs IVUS)**						
5	Randomized	Not serious	Not serious	Not serious	Not serious	⊕⊕⊕⊕<br>High
**Stent Thrombosis (IVUS vs ICA)**						
12	Randomized	Moderate	Not serious	Not serious	Not serious	⊕⊕⊕<br>Moderate
**Stent Thrombosis (OCT vs ICA)**						
9	Randomized	Not serious	Not serious	Not serious	Not serious	⊕⊕⊕⊕<br>High
**Stent Thrombosis (OCT vs IVUS)**						
5	Randomized	Not serious	Not serious	Not serious	Not serious	⊕⊕⊕⊕<br>High
**Major Adverse Cardiac Events (IVUS vs ICA)**						
14	Randomized	Moderate	Not serious	Not serious	Moderate	⊕⊕<br>Serious
**Major Adverse Cardiac Events (OCT vs ICA)**						
9	Randomized	Not serious	Serious	Not serious	Moderate	⊕⊕<br>Serious
**Major Adverse Cardiac Events (OCT vs IVUS)**						
5	Randomized	Not serious	Moderate	Not serious	Moderate	⊕⊕<br>Serious

ICA=Invasive Coronary Angiography; IVUS=Intravascular Ultrasound; OCT=Optimal Coherence Tomography.

SUPPLEMENTARY FIGURES

Online Figure 1. Flow Diagram.

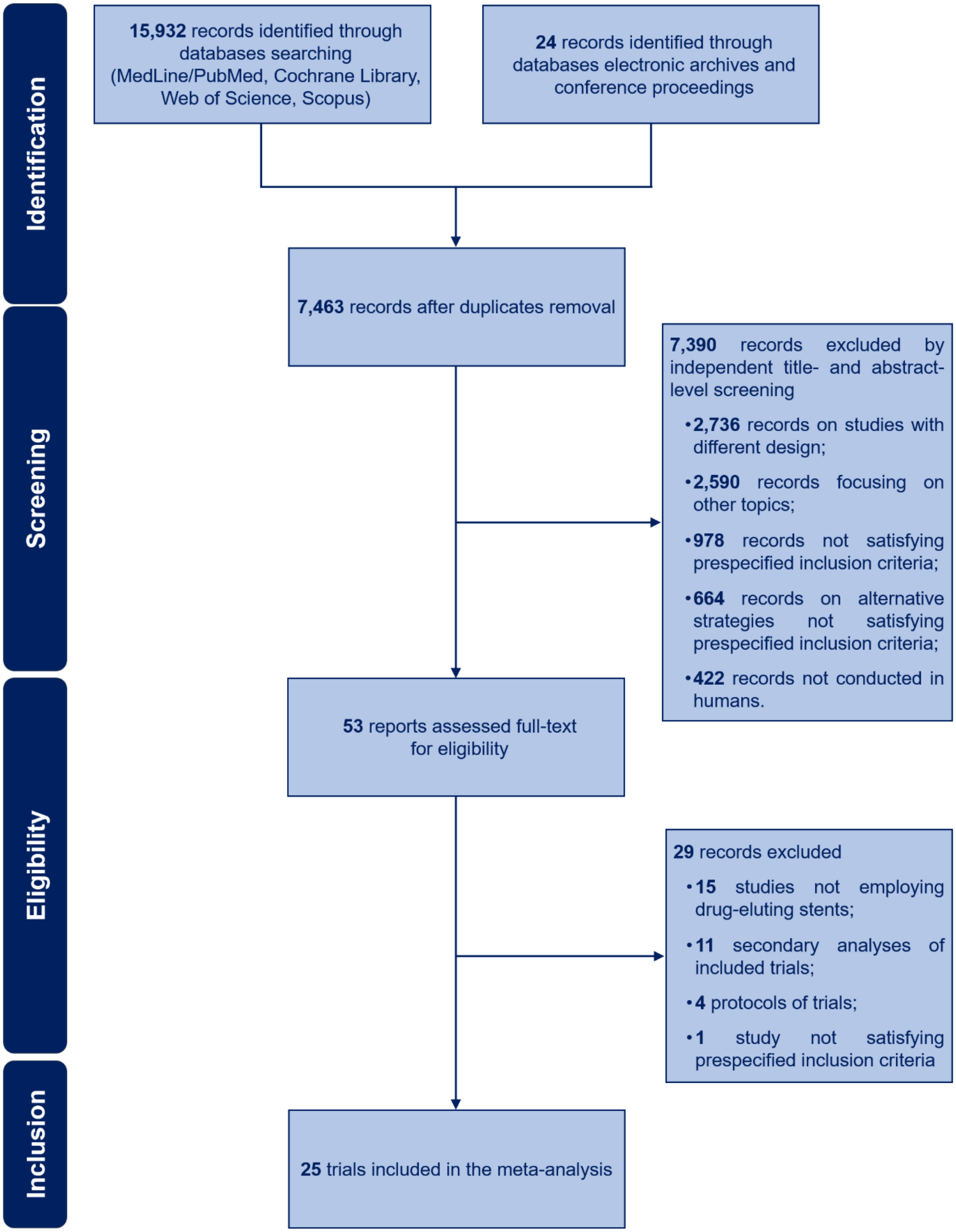

Online Figure 2. Risk of Bias by Individual Trials.

	D1	D2	D3	D4	D5	Overall
AIR-CTO	+	+	+	+	+	+
AVIO	-	+	+	+	+	-
CTO-IVUS	+	+	+	+	+	+
DOCTORS	+	?	+	+	+	?
EROSION III	+	+	+	+	+	+
GUIDE-DES	+	+	+	+	+	+
HOME DES IVUS	-	?	+	+	-	-
ILUMIEN III	+	+	+	+	+	+
ILUMIEN IV	+	+	+	+	+	+
iSIGHT	+	+	+	+	+	+
IVUS-XPL	+	+	+	+	+	+
Kala et al.	+	+	+	+	+	+
Kim et al.	+	?	+	+	+	?
Li et al.	?	?	+	+	?	?
Liu et al.	+	+	+	+	?	?
MISTIC-1	+	+	+	+	+	+
OCTACS	+	?	+	+	+	?
OCTIVUS	+	+	+	+	+	+
OCTOBER	+	+	+	+	+	+
OPINION	+	+	+	+	+	?
RENOVATE-COMPLEX-PCI	+	+	+	+	+	+
RESET	+	+	+	+	+	+
Tan et al.	-	-	+	+	?	-
ULTIMATE	+	+	+	+	+	+
Wang et al.	-	?	+	?	?	-

+ Low risk
? Some concerns
- High risk

D1 Randomization process
D2 Deviations from intervention
D3 Missing outcome data
D4 Measurment of the outcome
D5 Selection of reported results

D1-D5 are the domains of the RoB 2 tool.

Online Figure 3. Risk of Bias Across Trials.

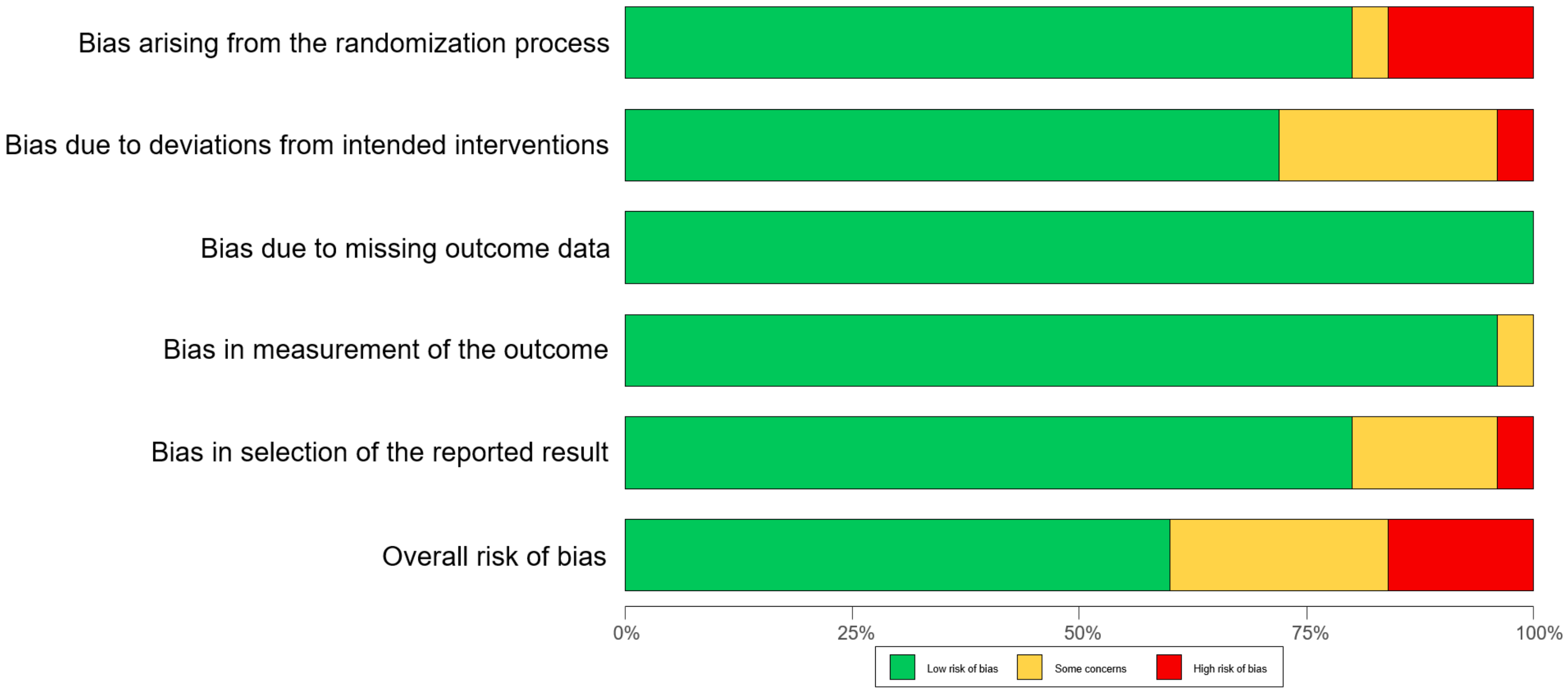

Online Figure 4. Chronologic Cumulative Meta-Analyses Across Direct Comparisons for Target Lesion Revascularization and Myocardial Infarction.

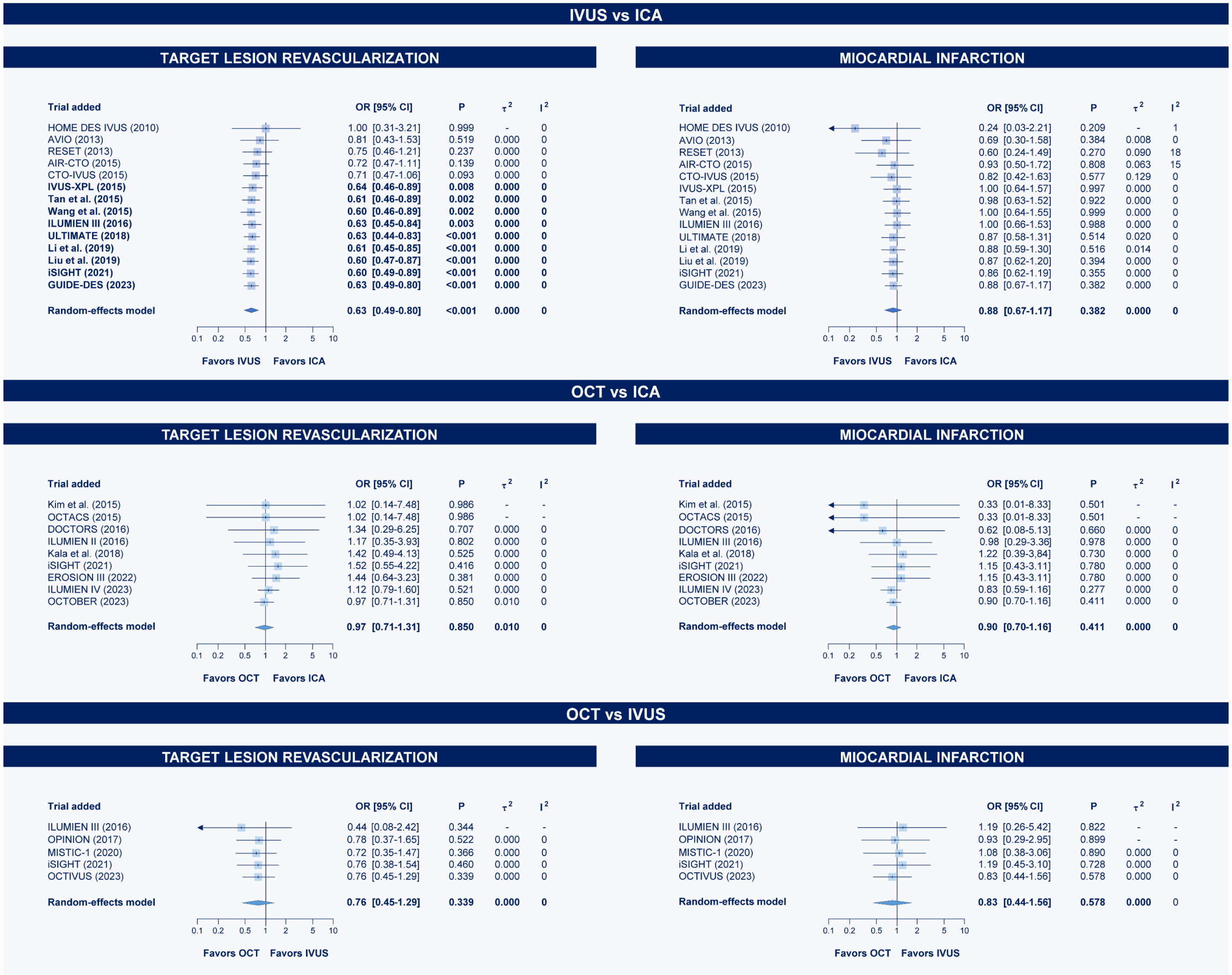

CI=Confidence Interval; ICA=Invasive Coronary Angiography; IVUS=Intravascular Ultrasound; OCT=Optical Coherence Tomography; OR=Odds Ratio.

Online Figure 5. Pairwise Direct Comparisons for Cardiac Death.

Trial	IVUS Events	IVUS Patients	ICA Events	ICA Patients	OR [95% CI]	W_F	W_R
AIR-CTO	3	115	5	115	0.59 [0.14-2.53]	5.3	5.3
AVIO	0	142	2	142	0.20 [0.01-4.14]	1.2	1.2
CTO-IVUS	0	201	2	201	0.20 [0.01-4.15]	1.2	1.2
GUIDE-DES	2	765	2	763	1.00 [0.14-7.10]	2.9	2.9
HOME DES IVUS	3	105	2	105	1.51 [0.25-9.26]	3.4	3.4
ILUMIEN III	0	136	0	142		0	0
iSIGHT	0	50	1	49	0.32 [0.01-8.07]	1.1	1.1
IVUS-XPL	3	700	5	700	0.60 [0.14-2.51]	5.5	5.5
Li et al.	0	120	1	108	0.32 [0.01-7.49]	1.1	1.1
Liu et al.	3	167	10	169	0.29 [0.08-1.08]	6.6	6.6
RESET	0	269	1	274	0.33 [0.01-8.32]	1.1	1.1
Tan et al.	2	61	3	62	0.67 [0.11-4.14]	3.4	3.4
ULTIMATE	9	724	16	724	0.56 [0.24-1.27]	16.6	16.6
Wang et al.	0	38	0	42		0	0
	**25**	**3593**	**50**	**3596**			
**Frequentist fixed-effect model**					**0.53 [0.33-0.86]**	**P=0.001**	
**Frequentist random-effects model**					**0.53 [0.33-0.86]**	**P=0.001**	
**Frequentist random-effects model with 95% CI adjustement**					**0.53 [0.31-0.91]**	**P=0.026**	
**Bayesian random-effects model**					**0.48 [0.26-0.81]***		
**Prediction interval**					**[0.31-0.92]**		

Heterogeneity: I^2=0%, τ^2=0, P=0.978

Trial	OCT Events	OCT Patients	ICA Events	ICA Patients	OR [95% CI]	W_F	W_R
DOCTORS	1	120	0	120	3.03 [0.12-75.00]	1.1	1.1
EROSION III	3	116	4	119	0.76 [0.17-3.49]	4.9	4.9
ILUMIEN III	0	153	0	142		0	0
ILUMIEN IV	9	1233	16	1254	0.57 [0.25-1.29]	16.7	16.7
iSIGHT	1	51	1	49	0.96 [0.06-15.79]	1.4	1.4
Kala et al.	1	105	0	96	2.94 [0.11-76.82]	1.1	1.1
Kim et al.	0	58	0	59		0	0
OCTACS	0	50	1	50	0.33 [0.01- 8.21]	1.1	1.1
OCTOBER	8	600	15	601	0.53 [0.22-1.25]	15.0	15.0
	**23**	**2486**	**37**	**2490**			
**Frequentist fixed-effect model**					**0.63 [0.37-1.06]**	**P=0.080**	
**Frequentist random-effects model**					**0.63 [0.37-1.06]**	**P=0.080**	
**Frequentist random-effects model with 95% CI adjustement**					**0.63 [0.33-1.20]**	**P=0.130**	
**Bayesian random-effects model**					**0.64 [0.33-1.36]***		
**Prediction interval**					**[0.32-1.24]**		

Heterogeneity: I^2=0%, τ^2=0, P=0.890

Trial	OCT Events	OCT Patients	IVUS Events	IVUS Patients	OR [95% CI]	W_F	W_R
ILUMIEN III	0	153	0	136		0	0
iSIGHT	1	51	0	50	3.04 [0.12-77.21]	1.1	1.1
MISTIC-1	1	54	0	55	3.07 [0.12-76.42]	1.1	1.1
OCTIVUS	3	1005	6	1003	0.50 [0.12-1.99]	5.8	5.8
OPINION	0	412	1	405	0.33 [0.01-8.07]	1.1	1.1
	**5**	**1675**	**7**	**1649**			
**Frequentist fixed-effect model**					**0.73 [0.24-2.22]**	**P=0.578**	
**Frequentist random-effects model**					**0.73 [0.24-2.22]**	**P=0.578**	
**Frequentist random-effects model with 95% CI adjustement**					**0.73 [0.12-4.43]**	**P=0.617**	
**Bayesian random-effects model**					**0.71 [0.18-8.37]***		
**Prediction interval**					**[0.06-7.30]**		

Heterogeneity: I^2=0%, τ^2=0, P=0.563

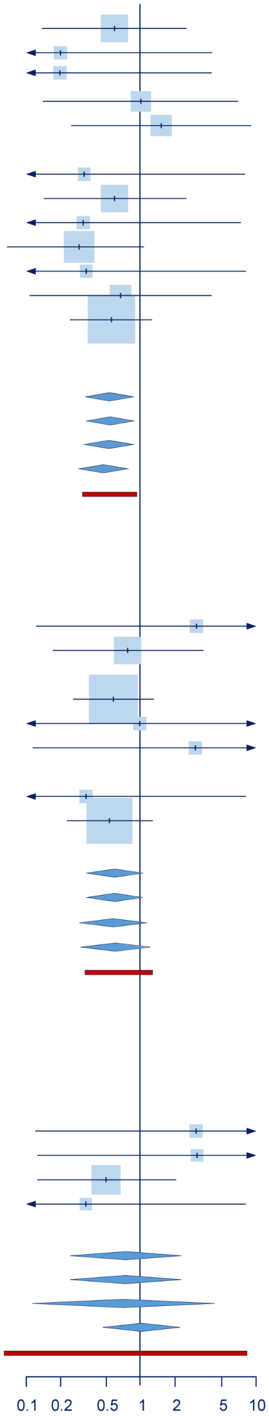

Test for differences across direct comparisons: χ^2=0.362, P=0.834

CI=Confidence Interval; ICA=Invasive Coronary Angiography; IVUS=Intravascular Ultrasound; OCT=Optical Coherence Tomography; OR=Odds Ratio.

* Credible Interval.

Online Figure 6. Pairwise Direct Comparisons for Stent Thrombosis.

Trial	IVUS Events	IVUS Patients	ICA Events	ICA Patients	OR [95% CI]	W_F	W_R
AIR-CTO	1	115	7	115	0.14 [0.02-1.12]	7.5	7.5
AVIO	1	142	0	142	3.02 [0.12-74.79]	3.2	3.2
CTO-IVUS	0	201	3	201	0.14 [0.01-2.74]	3.8	3.8
GUIDE-DES	5	765	4	763	1.25 [0.33-4.67]	19.2	19.2
HOME DES IVUS	4	105	6	105	0.65 [0.18-2.39]	19.9	19.9
ILUMIEN III	0	136	0	142	1.04 [0.02-53.00]	2.2	2.2
iSIGHT	0	50	0	49		0	0
IVUS-XPL	2	700	2	700	1.00 [0.14-7.12]	8.7	8.7
Li et al.	0	120	0	108		0	0
Liu et al.	2	167	5	169	0.40 [0.08-2.08]	12.2	12.2
RESET	1	269	1	274	1.02 [0.06-16.37]	4.3	4.3
Tan et al.	1	61	2	62	0.50 [0.04-5.66]	5.7	5.7
ULTIMATE	1	724	7	724	0.14 [0.02-1.15]	7.6	7.6
Wang et al.	1	38	2	42	0.54 [0.05-6.21]	5.6	5.6
	**19**	**3593**	**39**	**3596**			
**Frequentist fixed-effect model**					**0.57 [0.32-1.01]**	**P=0.056**	
**Frequentist random-effects model**					**0.57 [0.32-1.01]**	**P=0.056**	
**Frequentist random-effects model with 95% CI adjustement**					**0.57 [0.30-1.09]**	**P=0.082**	
**Bayesian random-effects model**					**0.47 [0.22-0.93]***		
**Prediction interval**					**[0.29-1.10]**		

Heterogeneity: Q=7.532, I^2=0%, τ^2=0, P=0.978

Trial	OCT Events	OCT Patients	ICA Events	ICA Patients	OR [95% CI]	W_F	W_R
DOCTORS	0	120	0	120		0	0
EROSION III	0	116	0	119		0	0
ILUMIEN III	1	153	0	142	1.86 [0.06-55.93]	3.9	3.9
ILUMIEN IV	6	1233	17	1254	0.36 [0.14-0.91]	52.1	52.1
iSIGHT	0	51	0	49		0	0
Kala et al.	1	105	1	96	0.91 [0.06-14.81]	5.9	5.9
Kim et al.	0	58	1	59	0.33 [0.01-8.21]	4.4	4.4
OCTACS	0	50	1	50	0.33 [0.01-8.21]	4.4	4.4
OCTOBER	5	600	5	601	1.00 [0.29-3.48]	29.4	29.4
	**13**	**2486**	**25**	**2490**			
**Frequentist fixed-effect model**					**0.55 [0.28-1.06]**	**P=0.073**	
**Frequentist random-effects model**					**0.55 [0.27-1.11]**	**P=0.094**	
**Frequentist random-effects model with 95% CI adjustement**					**0.55 [0.22-1.38]**	**P=0.155**	
**Bayesian random-effects model**					**0.54 [0.19-1.51]***		
**Prediction interval**					**[0.18-1.67]**		

Heterogeneity: Q=2.541, I^2=0%, τ^2=0.031, P=0.771

Trial	OCT Events	OCT Patients	IVUS Events	IVUS Patients	OR [95% CI]	W_F	W_R
ILUMIEN III	1	153	0	136	1.78 [0.06-53.56]	23.5	23.5
iSIGHT	0	51	0	50		0	0
MISTIC-1	0	54	0	55		0	0
OCTIVUS	0	1005	2	1003	0.20 [0.01-4.15]	29.5	29.5
OPINION	1	412	2	405	0.49 [0.04-5.43]	47.0	47.0
	**2**	**1675**	**4**	**1649**			
**Frequentist fixed-effect model**					**0.51 [0.10-2.63]**	**P=0.422**	
**Frequentist random-effects model**					**0.51 [0.10-2.63]**	**P=0.422**	
**Frequentist random-effects model with 95% CI adjustement**					**0.51 [0.01-19.01]**	**P=0.507**	
**Bayesian random-effects model**					**0.44 [0.04-3.24]***		
**Prediction interval**					**—**		

Heterogeneity: Q=0.888, I^2=0%, τ^2=0, P=0.642

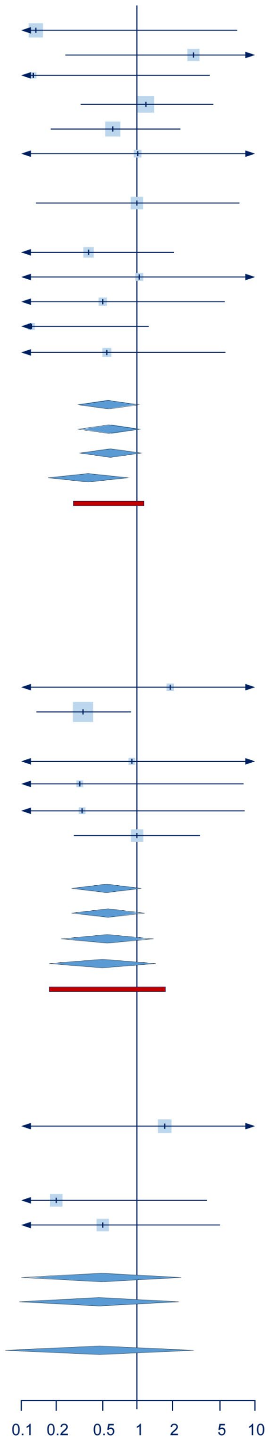

Test for differences across direct comparisons: χ^2=2.077, P=0.354

CI=Confidence Interval; ICA=Invasive Coronary Angiography; IVUS=Intravascular Ultrasound; OCT=Optical Coherence Tomography; OR=Odds Ratio.

* Credible Interval.

# Online Figure 7. Chronologic Cumulative Meta-Analyses Across Direct Comparisons for Ischemia-Driven Target Lesion Revascularization and Target Vessel Myocardial Infarction.

IVUS vs ICA

ISCHEMIA-DRIVEN TARGET LESION REVASCULARIZATION

Trial added	OR [95% CI]	P	τ^2	I^2
HOME DES IVUS (2010)	1.00 [0.31-3.21]	0.999	-	0
AVIO (2013)	0.81 [0.43-1.53]	0.519	0.000	0
RESET (2013)	0.75 [0.46-1.21]	0.237	0.000	0
AIR-CTO (2015)	0.72 [0.47-1.11]	0.139	0.000	0
CTO-IVUS (2015)	0.71 [0.47-1.06]	0.093	0.000	0
**IVUS-XPL (2015)**	**0.64 [0.46-0.89]**	**0.008**	**0.000**	**0**
**Tan et al. (2015)**	**0.61 [0.44-0.89]**	**0.002**	**0.000**	**0**
**Wang et al. (2015)**	**0.60 [0.44-0.83]**	**0.002**	**0.000**	**0**
**ILUMIEN III (2016)**	**0.63 [0.46-0.86]**	**0.003**	**0.000**	**0**
**ULTIMATE. 2018**	**0.63 [0.48-0.82]**	**<0.001**	**0.000**	**0**
**Li et al. (2019)**	**0.61 [0.47-0.79]**	**<0.001**	**0.000**	**0**
**Liu et al. (2019)**	**0.60 [0.47-0.78]**	**<0.001**	**0.000**	**0**
**iSIGHT (2021)**	**0.60 [0.47-0.78]**	**<0.001**	**0.000**	**0**
**GUIDE-DES (2023)**	**0.63 [0.49-0.80]**	**<0.001**	**0.000**	**0**
**Random-effects model**	**0.63 [0.49-0.80]**	**<0.001**	**0.000**	**0**

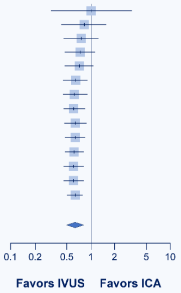

TARGET VESSEL MIOCARDIAL INFARCTION

Trial added	OR [95% CI]	P	τ^2	I^2
HOME DES IVUS (2010)	0.24 [0.03-2.21]	0.209	-	1
AVIO (2013)	0.69 [0.30-1.58]	0.384	0.008	0
RESET (2013)	0.60 [0.24-1.49]	0.270	0.090	18
AIR-CTO (2015)	0.93 [0.50-1.72]	0.808	0.063	15
CTO-IVUS (2015)	0.82 [0.42-1.63]	0.577	0.129	0
IVUS-XPL (2015)	1.00 [0.64-1.57]	0.997	0.000	0
Tan et al. (2015)	0.98 [0.63-1.52]	0.922	0.000	0
Wang et al. (2015)	1.00 [0.64-1.55]	0.999	0.000	0
ILUMIEN III (2016)	1.00 [0.65-1.55]	0.995	0.000	0
ULTIMATE (2018)	0.86 [0.56-1.31]	0.488	0.034	0
Li et al. (2019)	0.87 [0.57-1.31]	0.492	0.026	0
Liu et al. (2019)	0.86 [0.62-1.20]	0.383	0.000	0
iSIGHT (2021)	0.85 [0.61-1.19]	0.345	0.000	0
GUIDE-DES (2023)	0.88 [0.66-1.17]	0.371	0.000	0
**Random-effects model**	**0.88 [0.66-1.17]**	**0.371**	**0.000**	**0**

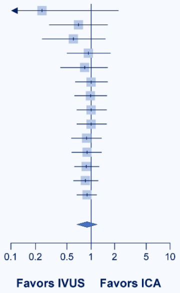

OCT vs ICA

ISCHEMIA-DRIVEN TARGET LESION REVASCULARIZATION

Trial added	OR [95% CI]	P	τ^2	I^2
Kim et al. (2015)	1.02 [0.14-7.48]	0.986	-	-
OCTACS (2015)	1.02 [0.14-7.48]	0.986	-	-
DOCTORS (2016)	1.34 [0.29-6.25]	0.707	0.000	0
ILUMIEN III (2016)	1.17 [0.35-3.93]	0.802	0.000	0
Kala et al. (2018)	1.42 [0.49-4.13]	0.525	0.000	0
iSIGHT (2021)	1.52 [0.55-4.22]	0.416	0.000	0
EROSION III (2022)	1.44 [0.64-3.23]	0.381	0.000	0
ILUMIEN IV (2023)	1.12 [0.79-1.60]	0.521	0.000	0
OCTOBER (2023)	0.96 [0.68-1.36]	0.851	0.010	0
**Random-effects model**	**0.96 [0.68-1.36]**	**0.851**	**0.010**	**0**

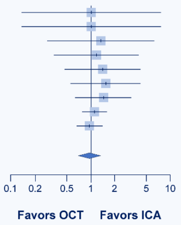

TARGET VESSEL MIOCARDIAL INFARCTION

Trial added	OR [95% CI]	P	τ^2	I^2
Kim et al. (2015)	0.33 [0.01-8.33]	0.501	-	-
OCTACS (2015)	0.33 [0.01-8.33]	0.501	-	-
DOCTORS (2016)	0.62 [0.08-5.13]	0.660	0.000	0
ILUMIEN III (2016)	0.72 [0.13-3.87]	0.702	0.000	0
Kala et al. (2018)	1.11 [0.25-4.88]	0.887	0.000	0
iSIGHT (2021)	1.06 [0.32-3.47]	0.928	0.000	0
EROSION III (2022)	1.06 [0.32-3.47]	0.928	0.000	0
ILUMIEN IV (2023)	0.80 [0.51-1.24]	0.314	0.000	0
OCTOBER (2023)	0.85 [0.63-1.15]	0.284	0.000	0
**Random-effects model**	**0.85 [0.63-1.15]**	**0.284**	**0.000**	**0**

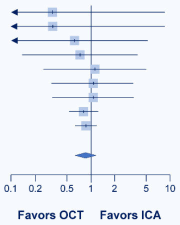

OCT vs IVUS

ISCHEMIA-DRIVEN TARGET LESION REVASCULARIZATION

Trial added	OR [95% CI]	P	τ^2	I^2
ILUMIEN III (2016)	2.29 [0.41-12.69]	0.344	-	-
OPINION (2017)	1.28 [0.60-2.69]	0.522	0.000	0
MISTIC-1 (2020)	1.39 [0.68-2.82]	0.366	0.000	0
iSIGHT (2021)	1.30 [0.65-2.60]	0.460	0.000	0
OCTIVUS (2023)	1.29 [0.77-2.18]	0.339	0.000	0
**Random-effects model**	**1.29 [0.77-2.18]**	**0.339**	**0.000**	**0**

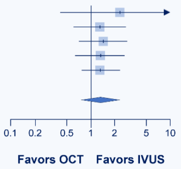

TARGET VESSEL MIOCARDIAL INFARCTION

Trial added	OR [95% CI]	P	τ^2	I^2
ILUMIEN III (2016)	0.89 [0.06-14.34]	0.933	-	-
OPINION (2017)	0.72 [0.16-3.23]	0.663	-	-
MISTIC-1 (2020)	0.96 [0.27-3.46]	0.950	0.000	0
iSIGHT (2021)	1.13 [0.36-3.50]	0.835	0.000	0
OCTIVUS (2023)	0.78 [0.40-1.54]	0.475	0.000	0
**Random-effects model**	**0.78 [0.40-1.54]**	**0.475**	**0.000**	**0**

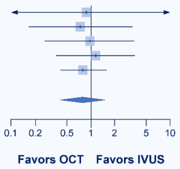

CI=Confidence Interval; ICA=Invasive Coronary Angiography; IVUS=Intravascular Ultrasound; OCT=Optical Coherence Tomography; OR=Odds Ratio.

Online Figure 8. Chronologic Cumulative Meta-Analyses Between IVI and ICA for Target Lesion Revascularization and Myocardial Infarction.

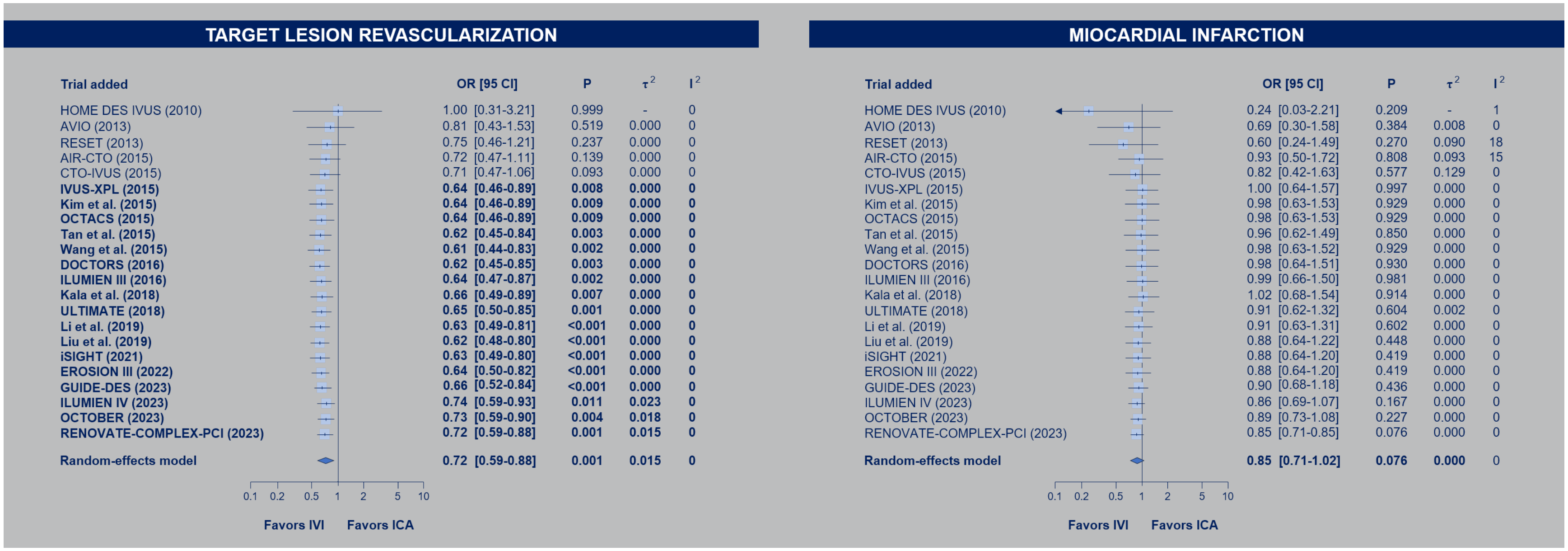

TARGET LESION REVASCULARIZATION

Trial added	OR [95 CI]	P	τ^2	I^2
HOME DES IVUS (2010)	1.00 [0.31-3.21]	0.999	-	0
AVIO (2013)	0.81 [0.43-1.53]	0.519	0.000	0
RESET (2013)	0.75 [0.46-1.21]	0.237	0.000	0
AIR-CTO (2015)	0.72 [0.47-1.11]	0.139	0.000	0
CTO-IVUS (2015)	0.71 [0.47-1.06]	0.093	0.000	0
**IVUS-XPL (2015)**	**0.64 [0.46-0.89]**	**0.008**	**0.000**	**0**
**Kim et al. (2015)**	**0.64 [0.46-0.89]**	**0.009**	**0.000**	**0**
**OCTACS (2015)**	**0.64 [0.46-0.89]**	**0.009**	**0.000**	**0**
**Tan et al. (2015)**	**0.62 [0.45-0.84]**	**0.003**	**0.000**	**0**
**Wang et al. (2015)**	**0.61 [0.44-0.83]**	**0.002**	**0.000**	**0**
**DOCTORS (2016)**	**0.62 [0.45-0.85]**	**0.003**	**0.000**	**0**
**ILUMIEN III (2016)**	**0.64 [0.47-0.87]**	**0.002**	**0.000**	**0**
**Kala et al. (2018)**	**0.66 [0.49-0.89]**	**0.007**	**0.000**	**0**
**ULTIMATE (2018)**	**0.65 [0.50-0.85]**	**0.001**	**0.000**	**0**
**Li et al. (2019)**	**0.63 [0.49-0.81]**	**<0.001**	**0.000**	**0**
**Liu et al. (2019)**	**0.62 [0.48-0.80]**	**<0.001**	**0.000**	**0**
**iSIGHT (2021)**	**0.63 [0.49-0.80]**	**<0.001**	**0.000**	**0**
**EROSION III (2022)**	**0.64 [0.50-0.82]**	**<0.001**	**0.000**	**0**
**GUIDE-DES (2023)**	**0.66 [0.52-0.84]**	**<0.001**	**0.000**	**0**
**ILUMIEN IV (2023)**	**0.74 [0.59-0.93]**	**0.011**	**0.023**	**0**
**OCTOBER (2023)**	**0.73 [0.59-0.90]**	**0.004**	**0.018**	**0**
**RENOVATE-COMPLEX-PCI (2023)**	**0.72 [0.59-0.88]**	**0.001**	**0.015**	**0**
**Random-effects model**	**0.72 [0.59-0.88]**	**0.001**	**0.015**	**0**

MIOCARDIAL INFARCTION

Trial added	OR [95 CI]	P	τ^2	I^2
HOME DES IVUS (2010)	0.24 [0.03-2.21]	0.209	-	1
AVIO (2013)	0.69 [0.30-1.58]	0.384	0.008	0
RESET (2013)	0.60 [0.24-1.49]	0.270	0.090	18
AIR-CTO (2015)	0.93 [0.50-1.72]	0.808	0.093	15
CTO-IVUS (2015)	0.82 [0.42-1.63]	0.577	0.129	0
IVUS-XPL (2015)	1.00 [0.64-1.57]	0.997	0.000	0
Kim et al. (2015)	0.98 [0.63-1.53]	0.929	0.000	0
OCTACS (2015)	0.98 [0.63-1.53]	0.929	0.000	0
Tan et al. (2015)	0.96 [0.62-1.49]	0.850	0.000	0
Wang et al. (2015)	0.98 [0.63-1.52]	0.929	0.000	0
DOCTORS (2016)	0.98 [0.64-1.51]	0.930	0.000	0
ILUMIEN III (2016)	0.99 [0.66-1.50]	0.981	0.000	0
Kala et al. (2018)	1.02 [0.68-1.54]	0.914	0.000	0
ULTIMATE (2018)	0.91 [0.62-1.32]	0.604	0.002	0
Li et al. (2019)	0.91 [0.63-1.31]	0.602	0.000	0
Liu et al. (2019)	0.88 [0.64-1.22]	0.448	0.000	0
iSIGHT (2021)	0.88 [0.64-1.20]	0.419	0.000	0
EROSION III (2022)	0.88 [0.64-1.20]	0.419	0.000	0
GUIDE-DES (2023)	0.90 [0.68-1.18]	0.436	0.000	0
ILUMIEN IV (2023)	0.86 [0.69-1.07]	0.167	0.000	0
OCTOBER (2023)	0.89 [0.73-1.08]	0.227	0.000	0
RENOVATE-COMPLEX-PCI (2023)	0.85 [0.71-0.85]	0.076	0.000	0
**Random-effects model**	**0.85 [0.71-1.02]**	**0.076**	**0.000**	0

CI=Confidence Interval; ICA=Invasive Coronary Angiography; IVI=Intravascular Imaging; OR=Odds Ratio.

Online Figure 9. Chronologic Cumulative Meta-Analyses Between IVI and ICA for Ischemia-Driven Target Lesion Revascularization and Target Vessel Myocardial Infarction.

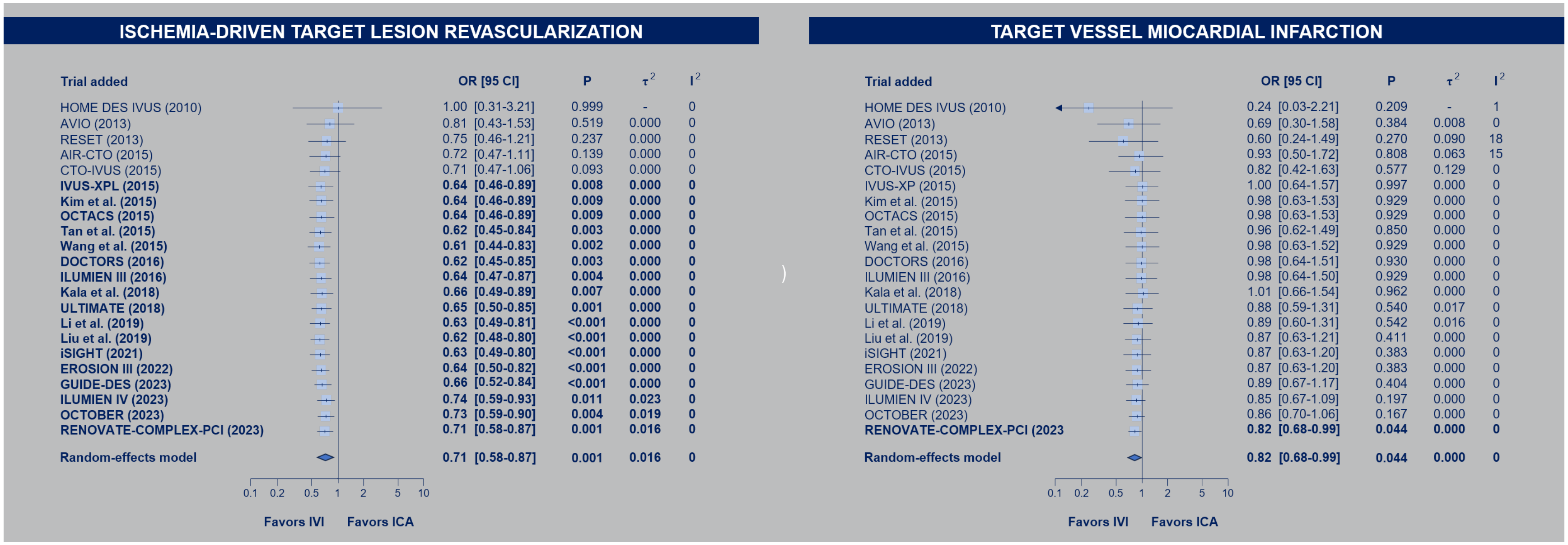

CI=Confidence Interval; ICA=Invasive Coronary Angiography; IVI=Intravascular Imaging; OR=Odds Ratio.

Online Figure 10. Comparison-Adjusted Funnel Plots – Network Meta-Analyses.

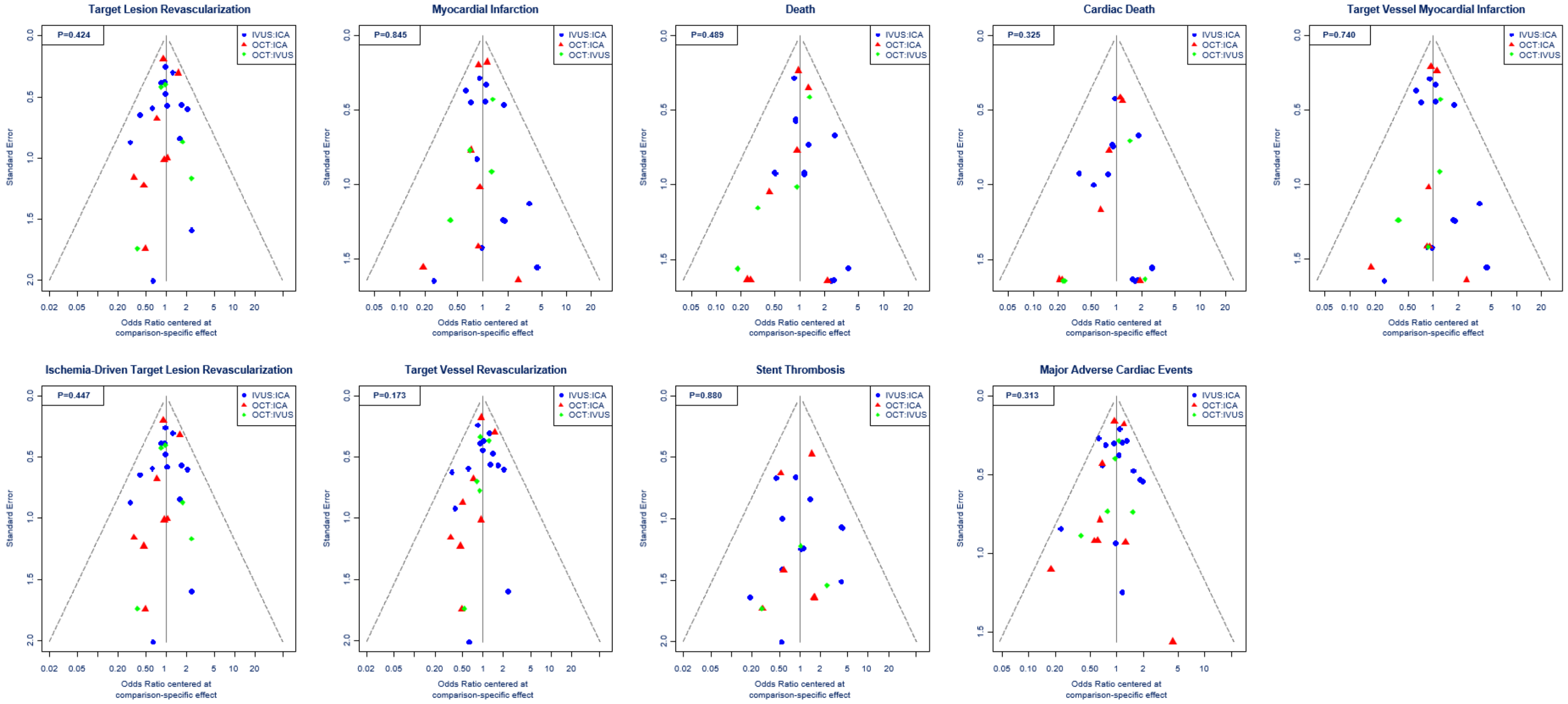

ICA=Invasive Coronary Angiography; IVUS=Intravascular Ultrasound; OCT=Optical Coherence Tomography.

The P value refers to the results of the Egger's test.

Online Figure 11. Contour-Enhanced Funnel Plots – Pairwise Meta-Analyses IVI- vs ICA-guided PCI.

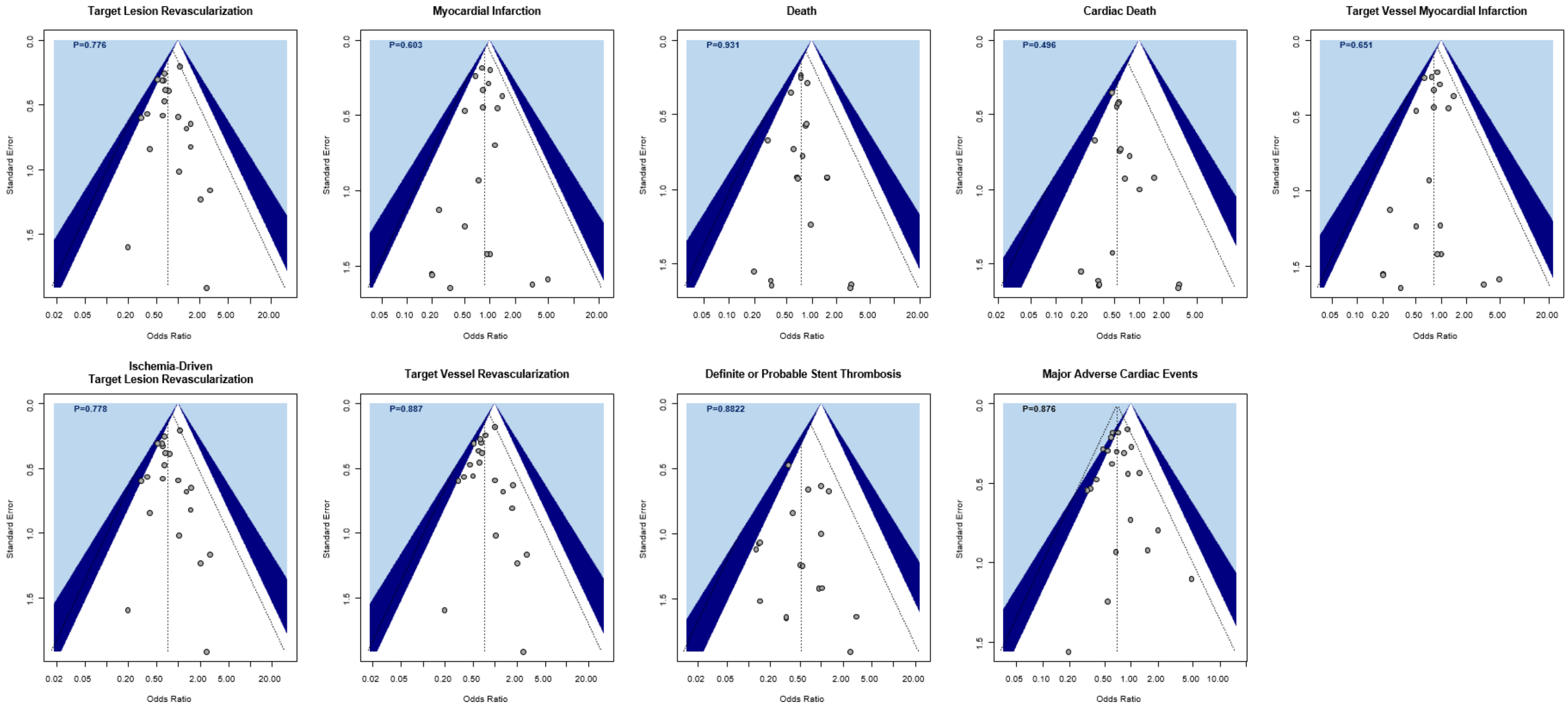

ICA=Invasive Coronary Angiography; IVUS=Intravascular Ultrasound; OCT=Optical Coherence Tomography.

The light blue area refers to highly significant effects (P<0.01), the dark blue area refers the significant effects (P between <0.01 and 0.05), and the white refers to nonsignificant effects (P>0.05).

The P value reported in the figures refers to the results of the Egger's test.
